# Sustainable Access to Acridin-9-(10*H*)ones with an Embedded *m*-Terphenyl Moiety Based on a Three-Component Reaction

**DOI:** 10.3390/molecules25235565

**Published:** 2020-11-27

**Authors:** Damiano Rocchi, Jorge Gómez-Carpintero, Juan F. González, Jose Carlos Menéndez

**Affiliations:** Unidad de Química Orgánica y Farmacéutica, Departamento de Química en Ciencias Farmacéuticas, Facultad de Farmacia, Universidad Complutense, 28040 Madrid, Spain; rocchid83@gmail.com (D.R.); jgomez01@ucm.es (J.G.-C.)

**Keywords:** multicomponent reactions, dihydroarenes, acridines, terphenyls

## Abstract

A Ce(IV)-catalyzed three-component reaction between chalcones, anilines and β-ketoesters followed by a microwave-assisted thermal cyclization afforded 1,3-diaryl-1,2-dihydroacridin-9(10*H*)-ones. Their microwave irradiation in nitrobenzene, acting both as solvent and oxidant, afforded fully unsaturated 1,3-diarylacridin-9(10*H*)-ones, which combine acridin-9-(10*H*)one and *m*-terphenyl moieties. Overall, the route generates three C-C and one C-N bond and has the advantage of requiring a single chromatographic separation.

## 1. Introduction

**Figure 1:**
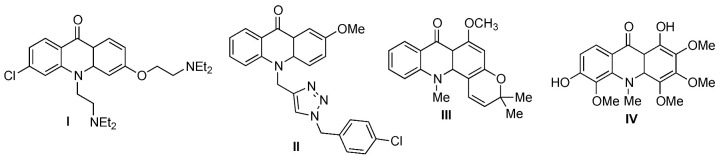
Selected bioactive 9-acridones.

The 9-acridone heterocyclic system is present in diverse alkaloid structures such as melicopicine, melicopidine and eroxantine [1], which have been isolated from *Melicopoe fareana*, *Sarcomelicope follicularis* and *Evodia xanthoxyloids*, respectively. Furthermore, the 9-acridone framework can be considered a privileged structures in the field of drug discovery as many derivates of this scaffold have shown a great variety of biological activities, such as antimalarial [2], antibacterial [3], antileishmanial [4], antiviral [5], anti-inflammatory and anti-neurodegenerative [6]. Additionally, it is well known that the planarity of these compounds allows them to act as insert in DNA and RNA, making them good candidates for their use as antitumor agents [7,8]. In Figure 1 we summarize some acridone structures that have shown interesting biological activities. For example, **I** has shown a good antimalarial activity, **II** displayed cholinesterase inhibition activity, which has great relevance in Alzheimer’s disease, acronicyne **III** and compounds **IV** have antineoplastic properties. Furthermore, due to their high fluorescence quantum yields, these molecules are attracting great attention in several technological fields, such as the development of luminescent probes and photoluminescent materials [9,10,11,12].

**Scheme 1:**
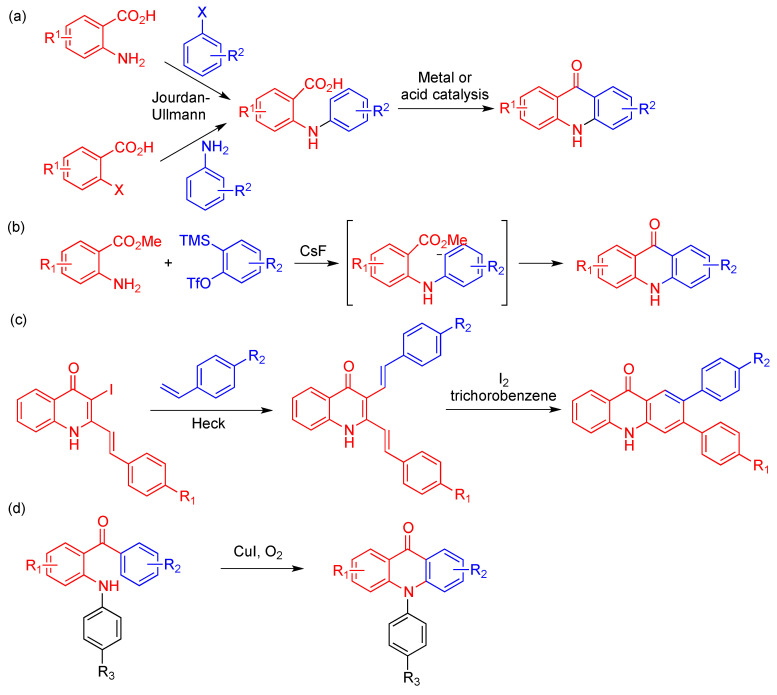
Main reported synthetic approaches to acridin-9-ones. Methods based on: (**a**) Jourdan-Ullmann coupling; (**b**) reactions of anthranilic esters with arynes; (**c**) iodine-promoted oxidative cyclizations; (**d**) oxidative cyclizations of *o*-arylamino benzophenones.

The most common synthetic access to 9-acridones involves the formation of the nitrogen ring from *N*-phenylanthranilic acid derivatives obtained via Jourdan–Ullmann couplings and heterocycle formation by the use of strong acids or catalyzed by metals [13,14,15] (Scheme 1a). An alternative approach reported by Larock and coworkers is based on the nucleophilic coupling of anthranilate with benzyne, which is formed in situ from a trimethylsilylphenyl triflate and cesium fluoride (Scheme 1b) [16]. Silva et al. described a new synthetic approach for the synthesis of 2,3-diarylacridin-9-ones, with a Heck coupling reaction between a substituted styryl and quinolone moiety as key synthetic step, followed of oxidative cyclization promoted by iodine (Scheme 1c) [17]. Deng research group described a synthetic alternative, in which the merged system is generated in the last reaction step, by oxidative cyclization of *o*-arylamino benzophenones (Scheme 1d) [18].

**Scheme 2:**
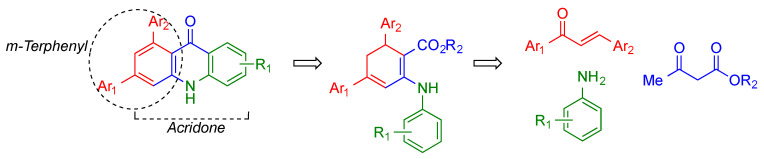
Our planned route to 1,3-diarylacridin-9-ones.

In spite of significant progress in the chemistry of this heterocyclic framework [19,20], some structural types of 9-acridones of potential interest in fields such as medicinal chemistry and materials chemistry have received little attention owing to limitations in the existing synthetic methodology. In particular, 1,3-diphenylacridin-9-ones are unknown in the literature despite the fact that they combine the acridone framework with an additional attractive structural fragment, namely *m*-terphenyl, which is important in materials science due to its high fluorescence [21] and also shows a variety of pharmacological activities [22,23,24,25]. In this article we describe our work towards addressing this synthetic challenge according to the strategy summarized in Scheme 2, which combines a multicomponent reaction with a 6π thermal electrocyclic reaction and a dehydrogenation step.

## 2. Results and Discussion

**Scheme 3:**
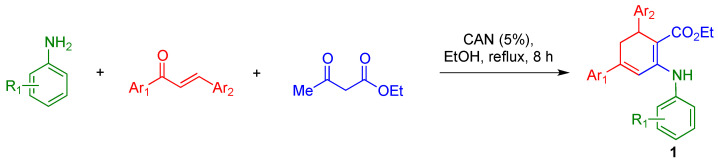
Three-component synthesis of dihydroterphenyl derivatives **1** from chalcones, anilines and β-ketoesters.

The route started with the synthesis of functionalized dihydroterphenyl derivatives from chalcones, anilines and β-ketoesters (Scheme 3), using a Ce(IV) ammonium nitrate (CAN)-catalyzed three-component protocol previously described by our group [26]. These reactions proceeded generally in good yields (Table 1) and allowed the introduction of sterically and electronically diverse substituents at both phenyl radicals, as well as some heteroaryls (compounds **1j** and **1k**). The presence of the N-aryl side branch, which could contain either electron-releasing or electron-withdrawing groups, was the basis for the subsequent electrocyclic cyclization step.

**Scheme 4:**
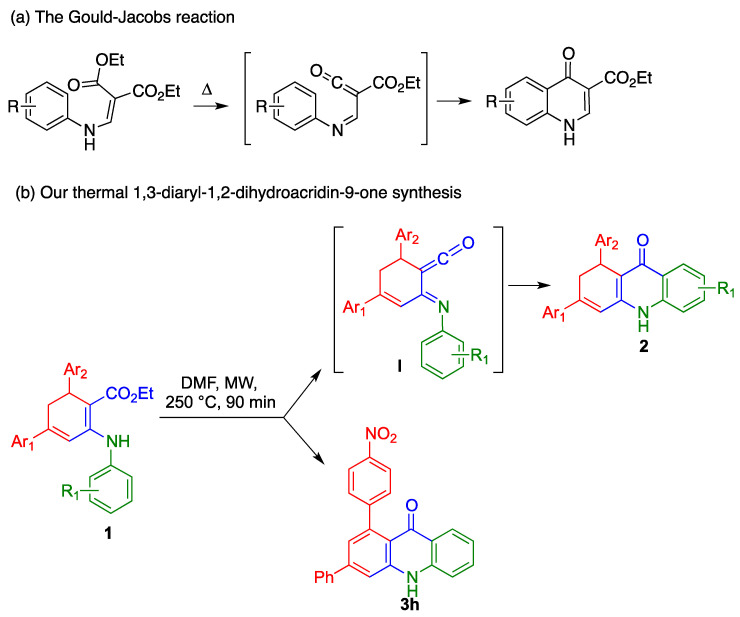
Microwave-assisted cyclization of *m*-terphenyl-derived aminoesters **1** to 1,3-diaryl-1,2-dihydroacridin-9(10*H*)-ones **2.**

The next step was to establish the reaction conditions for the cyclization of compounds **1** to the dihydroacridone derivatives **2**. These cyclizations are mainly described in the literature from carboxylic acids or aldehydes [27,28], which would require an additional step in our synthetic sequence. Based on the hypothesis that under thermal conditions the unsaturated β-aminoester moiety would provide an α-iminoketene intermediate **I** with loss of methanol, partially supported by the work of Wentrup et al. on the synthesis of 1-azafulven-6-one from pyrrole 2-carboxylic acid under flash vacuum pyrolysis [29,30], we decided to explore the ring-closing reaction of compounds **1** via a 6π electrocyclization reaction (Scheme 4). Based on our previous experience on microwave-enhanced cyclization reactions [30,31], we investigated the reactivity of the model compound **1a** under microwave conditions, which have not been previously reported for the synthesis of acridones, although they have been used in the classical Gould-Jacobs synthesis of 4-quinolones from anilines and ethyl ethoxymethylenemalonate [32,33,34], as shown in Scheme 4a. After a brief study of the parameter set, we found the optimal reaction conditions, which involved heating up to 250 °C for 90 min, using dimethylformamide as a solvent and an irradiation power of 200 W. The reaction was then concentrated and the residue was crushed with diethyl ether to furnish compound **2a** in 94% yield. In view of this excellent result, we extended the scope of this reaction to the full dihydroacridine library, with no clear-cut substituent effects being observed (Scheme 4b and Table 2). Compound **2h**, containing a nitro group at para position of the Ar_2_ ring, could not be isolated due its aromatization to **3h** under the cyclization reaction conditions. This can be due to the ability of the aromatic nitro group to participate as an intermediate in single electron-transfer processes, which facilitate molecular oxygen-promoted dehydrogenation reactions [35].

**Scheme 5:**
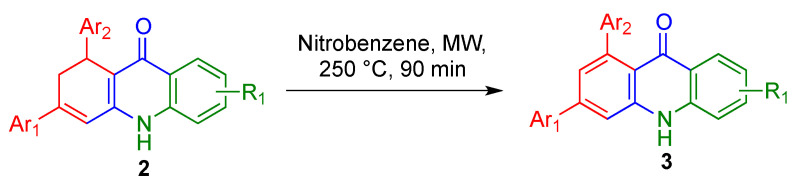
Aromatization of compounds **2**, with nitrobenzene having the dual role of solvent and oxidizing reagent.

Finally, we investigated the aromatization of compounds **2** to the corresponding 1,3-diarylacridin-9-ones **3** (Scheme 5). The optimization of the reaction conditions was carried out on compound **2a** as substrate and several dehydrogenating agents were tested. Palladium supported on carbon, manganese oxide in toluene at reflux conditions were tried without success. N-bromosuccinimide was also used tried as an aromatizing agent via halogenation-elimination [31] but these conditions also failed. DDQ in toluene (at room temperature, 120 min) and nitrobenzene (microwave, 250 °C, 90 min) were successful dehydrogenating reagents but the latter gave a higher yield and allowed a simpler purification process, as the reaction mixture could be purified by concentration in vacuo followed by trituration of the residue with diethyl ether to give the purified product by simple filtration. These conditions were applied to the whole compound library, with the results shown in Table 3.

**Scheme 6:**
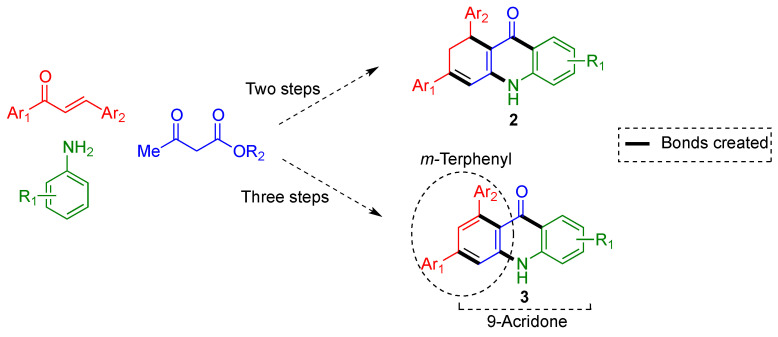
A summary of bond formation in our synthetic pathway.

To summarize, we have developed a method that affords 9-acridone derivatives containing an embedded *m*-terphenyl substructure with the generation of two rings, three carbon-carbon and one carbon-nitrogen bonds (Scheme 6). This process provides a very efficient access in two steps to 1,2-dihydroacridin-9-one derivatives, which are almost unknown in the literature [36] and not at all with the 1,3-diaryl substitution found in compounds **2**. Our method also allows the efficient synthesis of the fully unsaturated compounds **3** by adding a simple dehydrogenation step to the sequence.

One important aspect of our method is its relevance in terms of sustainability. On one hand, atom economy is high (e.g., 81% for **2a** and 80% for **3a**); on the other, the use of organic solvents is minimized by the fact that the second and third steps of the route yield products with sufficient purity to allow purification by simple precipitation, thus avoiding the waste generation associated to chromatographic processes.

## 3. Materials and Methods

### 3.1. General Experimental Information

All reagents and solvents were of commercial quality and were used as received. Reactions were monitored by TLC analysis, on Merck silica gel-G aluminum plates with fluorescent indicator. Melting points were measured in open capillary tubes and are uncorrected. A CEM Discover microwave synthesizer with microwave power maximum level of 300 W and microwave frequency of 2455 MHz was employed for the microwave-assisted reactions. The ^1^H-NMR, ^13^C-NMR and CH-correlation spectra were recorded on a Bruker (Avance) 250 MHz or 500 MHz NMR instrument maintained by the CAI de Resonancia Magnética Nuclear, Universidad Complutense, using CDCl_3_, d_6_-DMSO or CD_3_OD as solvents and residual non-deuterated solvents as internal standards. Topspin (Bruker) or Mestrenova (Mestrelab) software packages were used throughout for data processing; chemical shifts are given in parts per million (δ-scale) and coupling constants are given in Hertz. Subjective ^13^C-NMR assignments are based on 2d_NMR experiments for representative compounds, summarized in the Supporting Information. Combustion microanalyses were performed by the CAI de Microanálisis Elemental, Universidad Complutense, on a Leco 932 CHNS analyzer. IR spectra were recorded on a Perkin Elmer Paragon 1000 FT-IR instrument using thin films placed on a KBr disk, which were obtained by evaporation of organic solvent solution of the compounds.

### 3.2. General Procedure for the Synthesis of 2,4-Diaryl-2,3-dihydroanthranilates ***1***

To a stirred solution of ethyl acetoacetate (311.0 to 974.8 mg, 2.39 to 7.49 mmol) and aniline (281.3 to 906.8 mg, 3.02 to 9.74 mmol, 1.3 eq) in ethanol (5 mL) was added CAN (65.5 mg, 0.12 mmol, 5 mol%). Stirring was continued for 30 min at room temperature. The appropriate chalcone (730 mg to 2.0 g, 2.63 to 8.24 mmol, 1.1 eq) was then added to the stirred solution and the mixture was heated under reflux for 8 h. After completion of the reaction, as indicated by TLC, the mixture was dissolved in ether (30 mL), washed with water, brine, dried (anhydrous Na_2_SO_4_) and the solvent was evaporated under reduced pressure. The final products were purified by flash silica column chromatography eluting with a petroleum ether-ethyl acetate mixture (9/1, *v*/*v*). Compounds **1a**–**k** were known in the literature [26]. Characterization data for new compounds are given below (see Supplementary Materials). Compound numbering used in the assignment of ^13^C-NMR signals is also given.


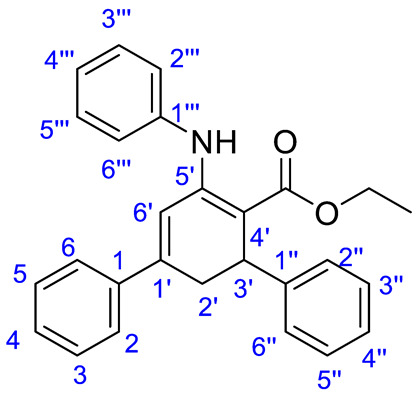


*Ethyl 4″-methoxy-5′-(phenylamino)-2′,3′-dihydro-[1,1′:3′,1″-terphenyl]-4″-carboxylate* (**1l**). Prepared from 1.78 g (7.45 mmol) of the corresponding chalcone. Yield: 2.7 g (6.26 mmol, 84%) as a yellow solid. Mp 105 °C. IR (cm^−1^): 3237, 3053, 2984, 2108, 1736, 1639. ^1^H-NMR (250 MHz, CDCl_3_) δ 10.64 (s, 1H), 7.27–7.15 (m, 6H), 7.12–7.07 (m, 2H), 7.06–6.99 (m, 3H), 6.70–6.62 (m, 2H), 6.56 (d, *J* = 2.8 Hz, 2H), 4.21 (dd, *J* = 8.5, 1.7 Hz, 1H), 4.03 (m, 2H), 3.64 (s, 3H), 3.11 (ddd, *J* = 16.6, 8.4, 2.9 Hz, 1H), 2.87 (dd, *J* = 16.6, 1.7 Hz, 1H), 1.10 (t, *J* = 7.1 Hz, 3H). ^13^C-NMR (63 MHz, CDCl_3_) δ 170.3 (CO), 156.0 (C-4″), 151.0 (C-5′), 144.1 (C-1″′), 140.0 (C-1′), 140.0 (C-1), 137.1 (C1″), 129.2 (C-5″′ and 3″′), 128.6 (C-3 and 5), 128.5 (C-2″ and C-6″), 128.3 (C-4), 125.9 (C-6 and C-2), 123.8 (C-4″′), 123.2, (C-6″′ and 2″′)118.0 (C6′), 113.5 (C-3″and C-5″), 95.7 (C-4′), 59.5(CH_3_-**CH_2_**O), 55.2 (MeO), 36.0 (C-3′), 34.8 (C-2″), 14.5 (**CH_3_**-CH_2_O). Anal. Calc. for C_28_H_27_O_3_N: C, 79.03; H, 6.40; N, 3.29. Found C, 78.83; H, 6.18; N, 3.09.

*Ethyl 4″-chloro-5′-(phenylamino)-2′,3′-dihydro-[1,1′:3′,1″-terphenyl]-4′-carboxylate* (**1m**). Prepared from 2.0 g (8.24 mmol) of the corresponding chalcone. Yield: 2.7 g (6.26 mmol, 76%) as a yellow solid. Mp: 120 °C. IR (cm^−1^): 3224, 3038, 2974, 2098, 1734, 1640. ^1^H-NMR (250 MHz, CDCl_3_) δ 10.76 (s, 1H), 7.38–7.06 (m, 14H), 6.64 (d, *J* = 2.8 Hz, 1H), 4.35–4.26 (m, 1H), 4.22–3.98 (m, 2H), 3.21 (ddd, *J* = 16.7, 8.6, 2.9 Hz, 1H), 2.92 (dd, *J* = 16.7, 1.7 Hz, 1H), 1.16 (t, *J* = 7.1 Hz, 3H). ^13^C-NMR (63 MHz, CDCl_3_) δ 170.2 (CO), 151.5 (C-5′), 144.0 (C-1″′), 143.7 (C-1″), 139.8 (C-1′), 131.8 (C-1), 129.3 (C-4″), 128.9 (C-5″′ and 3″′), 128.8 (C-2″ and C-6″), 128.8 (C-3″ and C-5″), 128.4 (C-3 and 5), 126.0 (C-2, C-4 and C-6), 124.1 (C-4″′), 123.4 (C-2″′ and 6″′)), 118.1 (C-6′), 94.7 (C4′), 59.6 (CH_3_-**CH_2_**O), 36.5 (C-3′), 34.6 (C-2′), 14.6 (**CH_3_**-CH_2_O). Anal. Calc. for C_27_H_24_O_2_NCl C, 75.43; H, 5.63; N, 3.26. Found C, 75.39; H, 5.54; N, 3.26.

*Ethyl 2″,4,4″-trimethoxy-5′-(phenylamino)-2′,3′-dihydro-[1,1′:3′,1″-terphenyl]-4′-carboxylate* (**1n**)**.** Prepared from 1.0 g (3.35 mmol) of the corresponding chalcone. Yield: 1.1 g (2.31 mmol, 69%) as a pale yellow solid (Mp: 110 °C. IR (cm^−1^): 3260, 2952, 2838, 2115, 1737, 1640.^1^H-NMR (250 MHz, CDCl_3_) δ 10.82 (s, 1H), 7.35 (dd, *J* = 8.4, 7.2 Hz, 2H), 7.28–7.16 (m, 4H), 7.11 (d, *J* = 7.3 Hz, 1H), 6.92 (dd, *J* = 8.0, 0.7 Hz, 1H), 6.83–6.77 (m, 2H), 6.58 (d, *J* = 2.9 Hz, 1H), 6.39 (d, *J* = 8.2 Hz, 2H), 4.25 (dt, *J* = 8.0, 1.2 Hz, 1H), 4.22–4.04 (m, 2H), 3.79 (s, 6H), 3.70 (s, 3H), 3.21 (ddd, *J* = 16.6, 8.3, 2.9 Hz, 1H), 2.87 (dd, *J* = 16.7, 1.7 Hz, 1H), 1.22 (t, *J* = 7.1 Hz, 3H). ^13^C-NMR (63 MHz, CDCl_3_) δ 170.7 (CO), 161.2 (C-4), 158.7 (C-4″), 158.1 (C-6″), 151.8 (C5′), 143.7 (C-1″′), 140.4 (C-1′), 137.8, 130.0 (C1, C1′, C1″ or C1″′), 129.3 (C-6, 2, 5″′ and 2″′), 128.8 (C-1 and C-2″), 123.7 (C-4″′), 123.6 (C-2″′ and 6″′), 123.3 (C-1″), 119.9 (C-6′), 113.6 (C-3 and C-5), 104.6 (C-3″), 99.2 (C-5″), 95.3 (C4′), 59.6 (CH_3_-**CH_2_**O), 55.8 (MeO), 55.7 (MeO), 55.6 (MeO), 36.9 (C-3′), 36.5 (C-2′**)** 14.5 (**CH_3_**-CH_2_O). Anal. Calc. for C_30_H_31_O_5_N C, 74.21; H, 6.44; N, 2.88. Found C, 73.89; H, 6.24; N, 2.90.

*Ethyl 4,4″-dichloro-5′-(phenylamino)-2′,3′-dihydro-[1,1′:3′,1″-terphenyl]-4′-carboxylate* (**1o**). Prepared from 730 mg (2.63 mmol) of the corresponding chalcone. Yield: 720 mg (1.55 mmol, 59%) as a yellow solid. Mp: 142 °C. IR (cm^−1^): 3219, 3056, 2976, 1898, 2099, 1639. ^1^H-NMR (250 MHz, CDCl_3_) δ 10.83 (s, 1H), 7.49–7.15 (m, 13H), 6.70 (d, *J* = 2.8 Hz, 1H), 4.38 (dd, *J* = 8.5, 1.7 Hz, 1H), 4.30–4.10 (m, 2H), 3.28 (ddd, *J* = 16.6, 8.5, 2.9 Hz, 1H), 2.95 (dd, *J* = 16.6, 1.8 Hz, 1H), 1.26 (t, *J* = 7.1 Hz, 3H). ^13^C-NMR (63 MHz, CDCl_3_) δ 170.3 (CO), 151.4 (C-5′), 143.7 (C-1″′), 142.9 (C-1″), 139.9 (C-1′), 138.4 (C-1), 134.9 (C-4), 132.2 (C-4″), 129.6 (C-3″′ and C-5″′), 129.2 (C-2″ and 6″), 129.0 (C-3 and 5), 128.6 (C-3″ and 5″), 127.4 (C-2 and C-6), 124.5 (C-4″′), 123.6 (C-2″′ and 6″′), 118.7 (C-6′), 95.1 (C-4′), 59.9 (CH_3_-**CH_2_**O), 36.7 (C-2′), 34.8 (C-3′), 14.8 (**CH_3_**-CH_2_O). Anal. Calc. for C_27_H_23_O_2_NCl_2_ C, 69.83; H, 4.99; N, 3.02. Found C, 69.51; H, 4.86; N, 3.02.

### 3.3. General Procedure for the Synthesis of 1,3-Diaryl-1,2-dihydroacridin-9(10H)-ones ***2***

A microwave tube containing a solution of the suitable compound **1** (150 to 474 mg, 0.3 to 1.0 mmol) in dimethylformamide (3 mL), was closed and placed in the cavity of a CEM Discover focused microwave oven. The reaction mixture was heated with a maximum power of 200 W and a temperature gradient was programmed to achieve 250 °C starting from room temperature over 5 min. Then temperature was kept constant at 250 °C, by microwave irradiated for 90 min. The reaction mixture was cooled to room temperature and the solvent was removed under reduced pressure. The crude mixture was washed with cool chloroform (3 mL) and the precipitate obtained was filtered to obtain the desired product. Compound **2h** was not obtained and its dehydrogenation derivative **3h** was isolated instead. Compound numbering used in the assignment of ^13^C-NMR signals is given below.


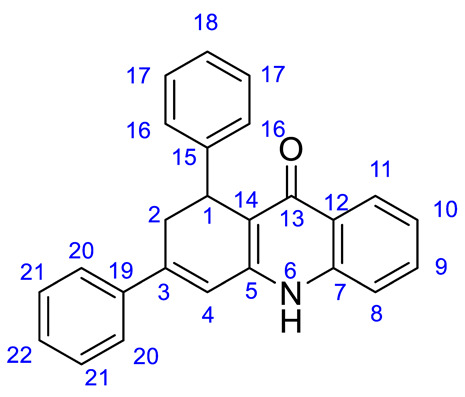


*1,3-Diphenyl-1,2-dihydroacridin-9(10H)-one* (***2a***). Prepared from compound **1a** (396 mg, 1.0 mmol). Yield: 328 mg (0.94 mmol, 94%); pale yellow solid. Mp: 287–288 °C. IR ν_max_ (film): 3064, 3027, 2874, 1623, 1572, 1541 cm^−1^. ^1^H-NMR (250 MHz, DMSO-*d*_6_) δ 11.83 (s, 1H), 8.06 (dd, *J* = 8.1, 1.1 Hz, 1H), 7.68–7.60 (m, 1H), 7.59–7.52 (m, 3H), 7.48–7.36 (m, 3H), 7.33–7.23 (m, 2H), 7.23–7.14 (m, 3H), 7.14–7.07 (m, 1H), 6.89 (d, *J* = 2.6 Hz, 1H), 4.61 (d, *J* = 7.7 Hz, 1H), 3.25 (ddd, *J* = 17.7, 7.7, 2.6 Hz, 1H), 3.08 (dd, *J* = 17.4, 1.3 Hz, 1H). ^13^C-NMR (63 MHz, DMSO-*d*_6_) δ 174.3 (CO), 145.3 (C-15), 144.5 (C-19), 143.6 (C-3), 139.3(C-12), 138.8 (C-19), 131.4 (C-9), 129.1 (C-22), 128.9 (C-21), 128.6 (C-17), 127.1 (C-16), 126.1 (C-18), 125.5 (C-20), 125.1 (C-7), 125.0 (C-11), 122.8 (C-10), 118.0 (C-8), 117.2 (C-4), 113.7 (C-14), 34.0 (C-1), 33.8 (C-2). Anal. Calc. for C_25_H_19_NO (M = 349.42): C, 85.93; H, 5.48; N, 4.01; found: C, 85.96; H, 5.52; N, 4.07.

*7-(Dimethylamino)-1,3-diphenyl-1,2-dihydroacridin-9(10H)-one* (**2b**). Prepared from compound **1b** (439 mg, 1.0 mmol). Yield: 377 mg, (0.96 mmol, 96%); yellow solid. Mp: 299–300 °C. IR ν_max_ (film): 2863, 2788, 1612, 1566, 1473 cm^−1^. ^1^H-NMR (500 MHz, DMSO-*d*_6_) δ 11.67 (s, 1H), 7.55 (d, *J* = 7.2 Hz, 2H), 7.46 (d, *J* = 9.1 Hz, 1H), 7.42 (t, *J* = 7.4 Hz, 2H), 7.40–7.34 (m, 1H), 7.27 (dd, *J* = 9.1, 2.9 Hz, 1H), 7.22 (d, *J* = 7.3 Hz, 3H), 7.14 (t, *J* = 7.5 Hz, 2H), 7.11–7.05 (m, 1H), 6.86 (d, *J* = 2.7 Hz, 1H), 4.63 (d, *J* = 8.2 Hz, 1H), 3.25 (ddd, *J* = 17.1, 8.4, 2.7 Hz, 1H), 3.07 (d, *J* = 16.3 Hz, 1H), 2.94 (s, 6H). ^13^C-NMR (126 MHz, DMSO-*d*_6_) δ 174.2 (CO), 147.7 (C-7), 145.8 (C-15), 145.0 (C-19), 142.8 C10, 139.8 (C-14), 132.2 (C-3), 129.7 (C-22), 129.7 (C-21), 128.8 (C-17), 127.9 (C-16), 127.1 (C-18), 126.7 (C-20), 126.2 (C-7), 120.2 (C-9), 119.8 (C-8), 118.3 (C-4), 113.0(C-12), 105.4 (C-11), 41.4 (Me_2_N), 34.8(C-1), 34.8 (C-2). Anal. Calc. for C_27_H_24_N_2_O (M = 392.49): C, 82.62; H, 6.16; N, 7.14; found: C, 82.59; H, 6.13; N, 7.17.

*7-Fluoro-1,3-diphenyl-1,2-dihydroacridin-9(10H)-one* (**2c**). Prepared from compound **1c** (414 mg, 1.0 mmol). Yield: 316 mg (0.86 mmol, 86%); pale yellow solid. Mp: 294–295 °C. IR ν_max_ (film): 2939, 2856, 2647, 1628, 1581 cm^−1^. ^1^H-NMR (250 MHz, DMSO-*d*_6_) δ 11.99 (s, 1H), 7.72 (dd, *J* = 9.5, 2.8 Hz, 1H), 7.68–7.51 (m, 5H), 7.49–7.37 (m, 3H), 7.26–7.16 (m, 2H), 7.15–7.08 (m, 2H), 6.90 (d, *J* = 2.6 Hz, 1H), 4.62 (d, *J* = 7.5 Hz, 1H), 3.33–3.23 (m, 1H), 3.10 (dd, *J* = 17.4, 1.3 Hz, 1H). ^13^C-NMR (63 MHz, DMSO-*d*_6_) δ 173.5 (CO), 158.2 (d, *J* = 484.0 Hz) (C-10), 145.7 (C-5), 144.3 (C-7), 143.8 (C-15), 138.7 (C-19), 136.0 (C-3), 129.2 (C-12), 128.9 (C-21), 128.1(C-22), 127.0 (C-17), 126.2 (C-20), 126.1 (C-16), 125.5 (C-18), 120.6 (d, *J* = 51.6 Hz) (C-9), 120.0 (C-8), 117.0 (C-4), 113.1 (C-14), 109.0 (d, *J* = 50.4 Hz) (C-11), 33.9(C-1), 33.8 (C-2). Anal. Calc. for C_25_H_18_FNO (M = 367.41): C, 81.72; H, 4.94; N, 3.81; found: C, 81.68; H, 4.93; N, 3.76.

*7-Chloro-1,3-diphenyl-1,2-dihydroacridin-9(10H)-one* (**2d**). Prepared from compound **1d** (430 mg, 1.0 mmol). Yield: 288 mg, (0.75 mmol, 75%); pale yellow solid. Mp: 275–276 °C. IR ν_max_ (film): 3060, 2923, 2887, 1620, 1542 cm^−1^. ^1^H-NMR (250 MHz, DMSO-*d*_6_) δ 12.01 (s, 1H), 7.99 (d, *J* = 2.3 Hz, 1H), 7.67 (dd, *J* = 8.8, 2.4 Hz, 1H), 7.62–7.54 (m, 3H), 7.49–7.35 (m, 4H), 7.20 (dd, *J* = 10.5, 7.4 Hz, 4H), 7.14–7.08 (m, 1H), 6.88 (d, *J* = 2.5 Hz, 1H), 4.58 (d, *J* = 7.5 Hz, 1H), 3.27 (d, *J* = 8.3 Hz, 1H), 3.09 (d, *J* = 16.5 Hz, 1H). ^13^C-NMR (63 MHz, DMSO-*d*_6_) δ 173.1 (CO), 146.0 (C-5), 144.2 (C-7), 138.7 (C-15), 137.9 (C-19), 131.5 (C-17), 129.2 (C-12), 129.0 (C-3), 128.1 (C-9), 127.4 (C-22), 127.0 (C-21), 126.1 (C-17), 126.0 (C-16), 125.6 (C-18), 124.0 (C-20), 122.8 (C-11), 120.5 (C-8), 117.0 (C4), 114.1 (C14), 33.9 (C1), 33.8 (C2). Anal. Calc. for C_25_H_18_ClNO (M = 383.87): C, 78.22; H, 4.73; N, 3.65; found: C, 78.18; H, 4.75; N, 3.61.

*7-Bromo-1,3-diphenyl-1,2-dihydroacridin-9(10H)-one* (**2e**). Prepared from compound **1e** (474 mg, 1.0 mmol). Yield: 330 mg, (0.77 mmol, 77%); pale yellow solid. Mp: 269–270 °C. IR ν_max_ (film): 3060, 2899, 2803, 1619, 1540 cm^−1^. ^1^H-NMR (250 MHz, DMSO-*d*_6_) δ 12.02 (s, 1H), 8.15 (d, *J* = 2.3 Hz, 1H), 7.79 (dd, *J* = 8.8, 2.3 Hz, 1H), 7.56 (t, *J* = 8.9 Hz, 3H), 7.48–7.35 (m, 3H), 7.21 (t, *J* = 8.8 Hz, 2H), 7.13 (dd, *J* = 6.5, 4.7 Hz, 2H), 6.89 (d, *J* = 2.2 Hz, 1H), 4.61 (d, *J* = 7.8 Hz, 1H), 3.27 (d, *J* = 8.4 Hz, 1H), 3.10 (d, *J* = 17.4 Hz, 1H). ^13^C-NMR (63 MHz, DMSO-*d*_6_) δ 173.0 (CO), 146.0 (C-5), 144.2 (C-7), 138.7 (C-15), 138.2 (C-19), 134.1 (C-9), 131.6 (C-12), 129.2 (C-3), 129.0 (C-11), 128.1 (C-21), 127.2 (C-17 and C-22), 127.0 (C-20), 126.4 (C-16), 126.2 (C-18), 125.6 (C-8), 120.7 (C-10), 120.6 (C-8), 115.4 (C4), 114.2 (C14), 33.9 (C1), 33.8 (C2). Anal. Calc. for C_25_H_18_BrNO (M = 428.32): C, 70.10; H, 4.24; N, 3.27; found: C, 70.06; H, 4.28; N, 3.21.

*6,8-Dichloro-1,3-diphenyl-1,2-dihydroacridin-9(10H)-one* (**2f**). Prepared from compound **1f** (464 mg, 1.0 mmol). Yield: 347 mg, (0.83 mmol, 83%); yellow solid. Mp: 199–200 °C. IR ν_max_ (film): 3255, 3058, 2896, 1628, 1579 cm^−1^. ^1^H-NMR (250 MHz, CDCl_3_-*d*_6_) δ 8.38 (s, 1H), 7.46–7.32 (m, 5H), 7.26 (s, 1H), 7.22–7.05 (m, 4H), 6.49 (d, *J* = 2.7 Hz, 1H), 6.41 (d, *J* = 1.7 Hz, 1H), 6.18 (d, *J* = 1.8 Hz, 1H), 4.70 (d, *J* = 7.8 Hz, 1H), 3.30 (ddd, *J* = 17.3, 8.7, 2.7 Hz, 1H), 3.12 (dd, *J* = 17.4, 1.5 Hz, 1H). ^13^C-NMR (63 MHz, CDCl_3_-*d*_6_) δ 179.0 (CO), 152.7 (C-7), 146.4 (C-5), 143.6(C-11), 143.4(C-9), 142.2 (C-15), 139.0 (C-19), 129.0 (C-3), 128.7(C-10), 128.3 (C-17 and 22), 127.1 (C-21), 126.5(C-12), 125.7 (C-20 and C-18), 116.6 (C-4), 114.9 (C14), 110.2 (C-14, 102.0 (C-8), 101.1 (C-14), 35.0 (C1), 34.6 (C2). Anal. Calc. for C_25_H_17_Cl_2_NO (M = 418.31): C, 71.78; H, 4.10; N, 3.35; found: C, 71.82; H, 4.16; N, 3.31.

*6,8-Dimethyl-1,3-diphenyl-1,2-dihydroacridin-9(10H)-one* (**2g**). Prepared from compound **1g** (424 mg, 1.0 mmol). Yield: 310 mg, (0.82 mmol, 82%); pale yellow solid. Mp: 275–276 °C. IR ν_max_ (film): 3238, 3079, 2955, 2918, 1621, 1585 cm^−1^. ^1^H-NMR (250 MHz, DMSO-*d*_6_) δ 11.46 (s, 1H), 7.57–7.49 (m, 2H), 7.39 (td, *J* = 8.1, 2.4 Hz, 3H), 7.20 (dt, *J* = 5.6, 2.9 Hz, 3H), 7.17–7.06 (m, 4H), 6.84 (d, *J* = 2.6 Hz, 1H), 6.79 (s, 1H), 4.52 (d, *J* = 10 Hz), 3.31–3.16 (m, 1H), 3.02 (d, *J* = 17.3 Hz, 1H), 2.75 (s, 3H), 2.35 (s, *J* = 5.7 Hz, 3H). ^13^C-NMR (63 MHz, DMSO-*d*_6_) δ 176.8 (CO), 144.7 (C-7), 144.4 (C-9), 142.2 (C-5), 141.1 (C-15), 140.3 (C-12), 139.0 (C-3), 138.9 (C-19), 128.9 (C-17), 128.0 (C-21), 127.1 (C-22), 126.9 (C-16), 125.9 (C-20), 125.4 (C-18), 121.4 (C-10), 117.1 (C-12), 115.5 (C-4), 114.6 (C-14), 34.4 (C-1), 33.9 (C-2), 23.2 (Me), 21.1 (Me). Anal. Calc. for C_27_H_23_NO (M = 377.48): C, 85.91; H, 6.14; N, 3.71; found: C, 85.86; H, 6.16; N, 3.67.

*3-(4-Bromophenyl)-1-phenyl-1,2-dihydroacridin-9(10H)-one* (**2i**). Prepared from compound **1i** (474 mg, 1.0 mmol). Yield: 407 mg, (0.95 mmol, 95%); yellow solid. Mp: 317–318 °C. IR ν_max_ (film): 2774, 1631, 1578, 1487 cm^−1^. ^1^H-NMR (250 MHz, DMSO-*d*_6_) δ 11.85 (s, 1H), 8.06 (d, *J* = 7.3 Hz, 1H), 7.68–7.59 (m, 3H), 7.53 (t, *J* = 8.2 Hz, 3H), 7.36–7.05 (m, 6H), 6.91 (d, *J* = 2.5 Hz, 1H), 4.60 (d, *J* = 7.9 Hz, 1H), 3.31–3.20 (m, 1H), 3.03 (d, *J* = 16.8 Hz, 1H). ^13^C-NMR (126 MHz, DMSO-*d*_6_) δ 174.1 (CO), 144.2 (C-5), 143.8 (C-15), 143.0 (C-7), 139.1 (C-3), 137.9 (C-19), 131.6 (C-21), 131.0 (C-9), 127.7 (C-17), 127.3 (C-16), 126.7 (C-20), 125.7 (C-12), 124.9 (C-18), 124.8 (C-11), 122.4 (C-10), 121.9 (C-22), 117.7 (C-8), 117.7 (C4), 113.7 (C14), 33.6 (C1), 33.6 (C2). Anal. Calc. for C_25_H_18_BrNO (M = 428.32): C, 70.10; H, 4.24; N, 3.27; found: C, 70.07; H, 4.26; N, 3.23.

*1-Phenyl-3-(thiophen-2-yl)-1,2-dihydroacridin-9(10H)-one* (**2j**). Prepared from compound **1j** (402 mg, 1.0 mmol). Yield: 334 mg, (0.94 mmol, 94%); orange solid. Mp: 304–305 °C. IR ν_max_ (film): 3068, 2923, 1619, 1572 cm^−1^. ^1^H-NMR (250 MHz, DMSO-*d*_6_) δ 11.82 (s, 1H), 8.06 (d, *J* = 8.0 Hz, 1H), 7.64 (dd, *J* = 11.2, 5.9 Hz, 2H), 7.53 (d, *J* = 8.2 Hz, 1H), 7.46 (d, *J* = 3.4 Hz, 1H), 7.35–7.06 (m, 7H), 6.86 (s, 1H), 4.61 (br s, 1H), 3.21 (br s, 2H). ^13^C-NMR (63 MHz, DMSO-*d*_6_) δ 174.4 (CO), 162.7 (C-5), 144.7 (C-15), 143.7(C-3), 143.1 (C-7), 139.6 (C-19), 139.5 (C-9), 131.7 (C-22), 128.9 (C-17), 128.4 (C-21), 128.4 (C-12), 127.4 (C-20), 127.1 (C-16), 126.4 (C-11), 125.4 (C-18), 123.1 (C-10), 118.3 (C-4), 115.0 (C-8), 114.1 (C14), 34.1 (C1), 34.0 (C2). Anal. Calc. for C_23_H_17_NOS (M = 355.45): C, 77.72; H, 4.82; N, 3.94; found: C, 77.67; H, 4.84; N, 3.91.

*1,3-Di(furan-2-yl)-1,2-dihydroacridin-9(10H)-one* (***2k***). Prepared from compound **1k** (375 mg, 1.0 mmol). Yield: 303 mg (0.92 mmol, 92%); pale yellow solid. Mp: 282–283 °C. IR ν_max_ (film): 3066, 2911, 1621, 1562 cm^−1^. ^1^H-NMR (250 MHz, DMSO-*d*_6_) δ 11.84 (s, 1H), 8.08 (d, *J* = 8.1 Hz, 1H), 7.84 (d, *J* = 1.6 Hz, 1H), 7.63 (t, *J* = 7.6 Hz, 1H), 7.52 (d, *J* = 7.8 Hz, 1H), 7.43 (s, *J* = 0.9 Hz, 1H), 7.29 (t, *J* = 8.0 Hz, 1H), 6.95 (d, *J* = 3.4 Hz, 1H), 6.84 (d, *J* = 2.3 Hz, 1H), 6.62 (dd, *J* = 3.4, 1.8 Hz, 1H), 6.19 (dd, *J* = 3.1, 1.8 Hz, 1H), 5.76 (d, *J* = 3.2 Hz, 1H), 4.62 (d, *J* = 7.4 Hz, 1H), 3.16 (dd, *J* = 17.1, 1.1 Hz, 1H), 2.91 (ddd, *J* = 17.1, 8.0, 2.5 Hz, 1H). ^13^C-NMR (63 MHz, DMSO-*d*_6_) δ 173.8 (CO), 156.3 (C-15), 152.2 (C-5), 145.1 (C-19), 143.5 (C-22), 141.5 (C-18), 139.3 (C-7), 134.1 (C-3), 131.4 (C-9), 125.1 (C-12), 125.0 (C-11), 122.8 (C-10), 118.0 (C4), 112.5(C-8), 112.5 (C-21), 111.4 (C-20), 111.4 (C-14), 110.2 (C-17), 105.1 (C-16), 28.7 (C1), 28.2 (C2). Anal. Calc. for C_21_H_15_NO_3_ (M = 329.35): C, 76.58; H, 4.59; N, 4.25; found: C, 76.53; H, 4.62; N, 4.28.

*3-(4-Methoxyphenyl)-1-phenyl-1,2-dihydroacridin-9(10H)-one* (**2l**). Prepared from compound **1l** (200 mg, 0.47 mmol); yield: 76 mg (0.20 mmol, 43% yield). yellow solid Mp: 242 °C. IR ν_max_ (film): 3391, 2990, 2770, 2106, 1607. ^1^H-NMR (250 MHz, DMSO-*d*_6_) δ 11.87 (s, 1H), 8.08 (dd, *J* = 8.1, 1.4 Hz, 1H), 7.68–7.53 (m, 4H), 7.48–7.37 (m, 3H), 7.29 (ddd, *J* = 8.1, 6.7, 1.3 Hz, 1H), 7.17–7.08 (m, 2H), 6.90 (d, *J* = 2.5 Hz, 1H), 6.78–6.65 (m, 2H), 4.57 (d, *J* = 7.8 Hz, 1H), 3.63 (s, 3H), 3.32–3.19 (ddd, *J* = 17.0, 8.3, 2.5 Hz, 1H), 3.10–3.01 (d, *J* = 17.0 Hz, 1H). ^13^C-NMR (63 MHz, DMSO-*d*_6_) δ 174.7 (CO), 157.9(C-22), 145.7 (C-5), 143.8 (C-15), 139.6 (C-7), 139.1 (C-3), 136.7 (C-9), 131.7 (C-19), 129.4 (C-20), 129.3 (C-17), 128.3 (C-16 and C-18), 125.8 C-12), 125.4 (C-11), 123.1 (C-10), 118.3 (C-8), 117.4 (C4), 114.5 (C-21), 113.7 (C14), 55.2 (OMe), 34.4 (C1), 33.3 (C2). Anal. Calc. for C_26_H_21_O_2_N C, 82.30; H, 5.58; N, 3.69. Found C, 80.91; H, 5.47; N, 3.85.

*3-(4-Chlorophenyl)-1-phenyl-1,2-dihydroacridin-9(10H)-one* (**2m**). Prepared from compound **1m** (300 mg, 0.7 mmol); yield: 140 mg (0.4 mmol, 52% yield). Brown solid Mp: 278 °C. IR ν_max_ (film): 3252, 3056, 2874, 2765, 2107, 1700, 1620. ^1^H-NMR (250 MHz, DMSO-*d*_6_) δ 11.90 (s, 1H), 8.06 (dd, *J* = 8.1, 1.4 Hz, 1H), 7.69–7.51 (m, 5H), 7.50–7.37 (m, 3H), 7.29 (m, 2H), 7.20 (m, 2H), 6.90 (d, *J* = 2.5 Hz, 1H), 4.59 (d, *J* = 8.1 Hz, 1H), 3.24 (dd, *J* = 8.5, 2.3 Hz, 1H), 3.07 (dd, *J* = 17.6, 1.4 Hz, 1H). ^13^C-NMR (63 MHz, DMSO-*d*_6_) δ 174.6 (CO), 145.6 (C-5), 144.0 (C-15), 143.8 (C-7), 139.6 (C-3), 139.0 (C-9), 131.8(C-19), 131.0 C-22), 129.5 (C-21), 129.3 (C-17), 129.3 (C-20 y C-16), 128.3 (C-11), 125.9 (C-12), 125.4 (C-18), 123.2 (C-10), 118.4 (C-8), 117.5 (C4), 113.6 (C14), 34.0 (C1), 33.6 (C2). Anal. Calc. for C_25_H_18_ONCl C, 78.22; H, 4.73; N, 3.65. Found C, 76.03; H, 4.85; N, 3.97.

*3-(2,4-Dimethoxyphenyl)-1-(4-methoxyphenyl)-1,2-dihydroacridin-9(10H)-one* (**2n**). Prepared from compound **1n** (150 mg, 0.3 mmol); 58 mg (0.13 mmol, 44% yield). Yellow solid Mp: 168 °C. IR ν_max_ (film): 3214, 3066, 2932, 2829, 2118, 1603. ^1^H-NMR (250 MHz, DMSO-*d*_6_) δ 12.14 (s, 1H), 8.15–8.07 (m, 1H), 7.65 (dd, *J* = 6.2, 1.5 Hz, 2H), 7.37–7.28 (m, 1H), 7.24 (dd, *J* = 8.5, 1.8 Hz, 1H), 7.20–7.11 (m, 2H), 6.86 (d, *J* = 2.4 Hz, 1H), 6.75 (dd, *J* = 8.9, 2.5 Hz, 2H), 6.66–6.60 (m, 1H), 6.57 (d, *J* = 2.4 Hz, 1H), 4.52 (d, *J* = 7.7 Hz, 1H), 3.81 (s, 3H), 3.79 (s, 3H), 3.66 (s, 3H), 3.28–3.17 (m, 1H), 2.92 (d, *J* = 17.3 Hz, 1H). ^13^C-NMR (63 MHz, DMSO-*d*_6_) δ 161.4 (CO), 158.7 (C-22), 157.9 (C-18 and C-20), 145.6 (C-5), 144.7 (C-7), 139.5 (C-3), 136.6 (C-15), 131.7 (C-9), 129.6 (C-16), 128.4 (C-12), 125.2 (C-20), 125.0(C-11), 121.8 (C-8), 118.6 (C-17),118.4 (C4), 114.0 (C-21), 113.6 (C14), 105.6 (C-19), 99.3 (C-21′), 56.0 (OMe), 55.7 (OMe), 55.3 (OMe), 36.3 (C2), 33.5 (C1). Anal. Calc. for C_28_H_25_O_4_N C, 76.52; H, 5.73; N, 3.19. Found C, 75.31; H, 5.67; N, 3.38.

*1,3-Bis(4-chlorophenyl)-1,2-dihydroacridin-9(10H)-one* (**2o**). Prepared from compound **1o** (173 mg, 0.3 mmol); 34 mg (0.08 mmol, 27% yield). Yellow solid Mp: 287 °C. IR ν_max_ (film): 3252, 3059, 2749, 2681, 2113, 1624. ^1^H-NMR (300 MHz, DMSO-*d*_6_) δ 11.86 (s, 1H), 8.07 (dd, *J* = 8.1, 1.5 Hz, 1H), 7.68–7.56 (m, 3H), 7.53–7.47 (m, 2H), 7.30 (ddd, *J* = 8.1, 6.8, 1.1 Hz, 2H), 7.23 (s, 4H), 6.91 (d, *J* = 2.7 Hz, 1H), 4.60 (d, *J* = 8.3 Hz, 1H), 3.29–3.22 (m, 1H), 3.04 (dd, *J* = 17.6, 1.5 Hz, 1H). ^13^C-NMR (75 MHz, DMSO-*d*_6_) δ 174.7 (CO), 144.3 (C-5), 143.8 (C-15), 139.7 (C-7), 138.0 (C-3), 134.1 (C-19), 132.0 (C-18), 131.1 (C-9), 129.4 (C-22), 129.3 (C-16), 128.5 (C-17), 127.8 (C-21), 125.5 (C-20), 125.4 (C-12), 124.5 (C-11), 123.4 (C-10), 118.5 (C-8), 118.3 (C4), 113.8 (C14), 34.0 (C1), 33.7 (C2). Anal. Calc. for C_25_H_17_ONCl_2_ C, 71.78; H, 4.10; N, 3.35. Found C, 71.04; H, 4.20; N, 3.49.

### 3.4. General Procedure for the Synthesis of 1,3-diaryl-acridin-9(10H)-ones ***3***

A microwave tube containing a solution of the suiTable 1,3-diaryl-1,2-dihydroacridin-9(10*H*)-one derivatives **2** (30 to 441 mg, 0.07 to 1.0 mmol) in nitrobenzene (3 mL), was closed and placed in the cavity of a CEM Discover focused microwave oven. The reaction mixture was heated by microwave irradiation for 90 min., at 200 W and 250 °C. Then, the mixture was cooled to room temperature and the solvent was evaporated under reduce pressure. The crude mixture was washed with cool chloroform and the solid obtained was filtered to obtain compounds 3. In the cases of compounds **3k** and **3n**, purification required column chromatography on silica gel, eluting with petrol ether/EtOAc (7/3). Compound numbering used in the assignment of ^13^C-NMR signals is given below.


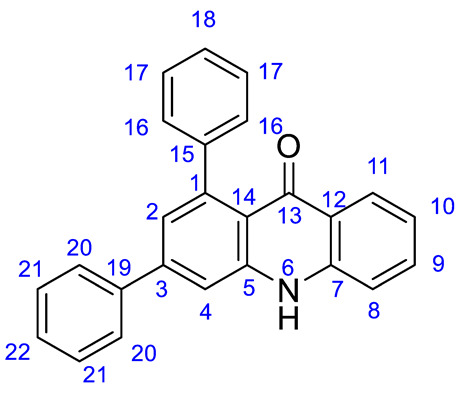


*1,3-Diphenylacridin-9(10H)-one* (**3a**). Prepared from compound **2a** (175 mg, 0.5 mmol). Yield: 142 mg, (0.41 mmol, 82%); brown solid. Mp: 333–334 °C. IR ν_max_ (film): 3062, 2972, 1624, 1594 cm^−1^. ^1^H-NMR (250 MHz, DMSO-*d*_6_) δ 8.03 (d, *J* = 8.1 Hz, 1H), 7.79 (d, *J* = 8.3 Hz, 3H), 7.71 (t, *J* = 6.9 Hz, 1H), 7.58–7.45 (m, 4H), 7.33 (bs, 5H), 7.24–7.14 (m, 2H). ^13^C-NMR (63 MHz, DMSO-*d*_6_) δ 176.5 (CO), 144.1 (C-5), 143.5 (C-3), 143.1 (C-7), 142.9 (C-19), 140.4 (C-15), 138.8 (C-1), 133.3 (C-9), 129.3 (C-21), 128.7 (C_17), 127.1 (C-20, C-16 and C-22) (3 overlapped signals), 126.3 (C-18), 126.2 (C-11), 123.2 (C-14), 121.9 (C-10), 121.0 (C-12), 116.8 (C-8), 116.6 (C-4), 114.5 (C-2). Anal. Calc. for C_25_H_17_NO (M = 347.41): C, 86.43; H, 4.93; N, 4.03; found: C, 85.94; H, 5.03; N, 4.06.

*7-(Dimethylamino)-1,3-diphenylacridin-9(10H)-one* (**3b**). Prepared from compound **2b** (196 mg, 0.5 mmol). Yield: 156 mg, (0.40 mmol, 80%); brown oil. IR ν_max_ (film): 2924, 2853, 1621, 1589 cm^−1^. ^1^H-NMR (250 MHz, CDCl_3_) δ 8.82 (s, 1H), 7.64 (dd, *J* = 7.9, 1.5 Hz, 2H), 7.50–7.39 (m, 8H), 7.39–7.32 (m, 2H), 7.24 (d, *J* = 1.7 Hz, 1H), 7.16 (d, *J* = 8.8 Hz, 1H), 7.01 (dd, *J* = 8.8, 2.7 Hz, 1H), 2.79 (s, 6H). ^13^C-NMR (63 MHz, CDCl_3_) δ 177.7 (CO), 144.9 (C-5), 144.6 (C-3), 144.0 (C-10), 143.8 (C-19), 141.8 (C-15), 139.6 (C-1), 133.2 (C-7), 129.1 (C-21), 128.5 (C-17), 128.4 (C-16), 127.5 (C-20), 127.4 (C-18), 126.6 (C-22), 124.0 (C-14), 123.9 (C_12), 122.8 (C-9), 117.5 (C-4), 116.7 (C-2), 114.3 (C-11), 105.2 (C-8), 31.3 (NMe). Anal. Calc. for C_27_H_22_N_2_O (M = 390.48): C, 83.05; H, 5.68; N, 7.17; found: C, 82.95; H, 6.03; N, 7.15.

*7-Fluoro-1,3-diphenylacridin-9(10H)-one* (**3c**). Prepared from compound **2c** (184 mg, 0.5 mmol). Yield: 155 mg, (0.42 mmol, 85%); pale yellow solid. Mp: 320–321 °C. IR ν_max_ (film): 3238, 3100, 2969, 1625, 1594, 1563 cm^−1^. ^1^H-NMR (250 MHz, DMSO-*d*_6_) δ 11.93 (bs, 1H), 7.84–7.73 (m, 3H), 7.69 (d, *J* = 8.0 Hz, 1H), 7.66–7.59 (m, 2H), 7.59–7.45 (m, 3H), 7.35 (bs, 5H), 7.19 (d, *J* = 0.8 Hz, 1H). ^13^C-NMR (63 MHz, DMSO-*d*_6_) δ 175.7 (CO), 157.0 (C_10) (d, *J* = 240 Hz), 143.9 (C-5), 143.6 (C_3), 142.9 (C-19), 142.8 (C-15), 138.7 (C-1), 137.2 (C-7), 129.3 C-17), 128.7 (C-21), 128.7 (C-16), 127.2 (C-20), 126.3 (C-18), 123.4 (C-22), 122.5 (C-14), 122.4 (C-12), 122.1 (C-9) (d, *J* = 25.2 Hz), 119.4 (C-8), 115.7 (C-2), 114.5 (C-4), 110.1 (d*, J* = 22.7 Hz) (C-11). Anal. Calc. for C_25_H_16_FNO (M = 365.40): C, 82.18; H, 4.41; N, 3.83; found: C, 82.13; H, 4.47; N, 3.81.

*7-Chloro-1,3-diphenylacridin-9(10H)-one* (**3d**). Prepared from compound **2d** (192 mg, 0.5 mmol). Yield: 145 mg, (0.38 mmol, 76%); yellow solid. Mp: 312–313 °C. IR ν_max_ (film): 3068, 2967, 1626, 1561 cm^−1^. ^1^H-NMR (250 MHz, DMSO-*d*_6_) δ 11.97 (s, 1H), 7.96 (d, *J* = 2.5 Hz, 1H), 7.81 (s, 1H), 7.79–7.74 (m, 3H), 7.72 (d, *J* = 2.5 Hz, 1H), 7.56 (dd, *J* = 7.9, 4.1 Hz, 3H), 7.49 (dd, *J* = 8.0, 3.7 Hz, 2H), 7.35 (s, 3H), 7.20 (d, *J* = 1.7 Hz, 1H). ^13^C-NMR (63 MHz, DMSO-*d*_6_) δ 175.4 (CO), 144.1 (C-5), 143.8 (C-3), 142.8 (C-19), 139.0 (C-15), 138.7 (C-7), 133.7 (C-1), 133.3 (C-9), 129.3 (C-11), 128.8 (C-17), 128.7 (C-21), 127.2 (C-16), 127.2 (C-20), 126.4 (C-18), 125.4 (C-22), 125.1 (C-14), 123.7 (C-12), 122.7 (C-8), 119.3 (C-10), 116.4 (C-4), 114.6 (C-2). Anal. Calc. for C_25_H_16_ClNO (M = 381.85): C, 78.63; H, 4.22; N, 3.67; found: C, 78.58; H, 4.26; N, 3.63.

*7-Bromo-1,3-diphenylacridin-9(10H)-one* (**3e**). Prepared from compound **2e** (214 mg, 0.5 mmol). Yield: 166 mg, (0.69 mmol, 78%); yellow solid. Mp: 276–277 °C. IR **ν_max_** (film): 3264, 3059, 2985, 1620, 1596 cm^−1^. ^1^H-NMR (250 MHz, DMSO-*d*_6_) δ 11.99 (s, 1H), 8.10 (d, *J* = 2.3 Hz, 1H), 7.83–7.79 (m, 2H), 7.76 (m, 3H), 7.58–7.52 (m, 2H), 7.52–7.46 (m, 3H), 7.38–7.33 (m, 3H), 7.20 (d, *J* = 1.6 Hz, 1H). ^13^C-NMR (63 MHz, DMSO-*d*_6_) δ 175.3 (CO), 144.1 (C-5), 143.8 (C-3), 142.8 (C-19), 139.3 (C-7), 138.7 (C-15), 135.8 (C-1), 129.3 (C-9), 128.8 (C-11), 128.7 (C-17), 128.3 (C-21), 127.2 (C-16), 127.2 (C-20), 127.1 (C-18), 126.4 (C-229, 123.7 (C-14), 123.2 (C-12), 119.5 (C-8), 116.5 (C-10), 114.6 (C-4), 113.1 (C-2). Anal. Calc. for C_25_H_16_BrNO (M = 426.30): C, 70.43; H, 3.78; N, 3.29; found: C, 70.39; H, 3.73; N, 3.34.

*6,8-Dichloro-1,3-diphenylacridin-9(10H)-one* (**3f**). Prepared from compound **2f** (209 mg, 0.5 mmol). Yield: 162 mg, (0.39 mmol, 78%); yellow solid. Mp: 281–282 °C. IR ν_max_ (film): 3279, 3060, 2923, 1620, 1595 cm^−1^. ^1^H-NMR (250 MHz, DMSO-*d*_6_) δ 11.61 (s, 1H), 9.68 (d, *J* = 5.0 Hz, 1H), 7.75 (d, *J* = 7.2 Hz, 2H), 7.62 (s, 1H), 7.57–7.43 (m, 3H), 7.39–7.22 (m, 4H), 7.09 (d, *J* = 0.8 Hz, 1H), 6.51 (d, *J* = 1.0 Hz, 1H), 6.10 (d, *J* = 1.0 Hz, 1H). ^13^C-NMR (63 MHz, DMSO-*d*_6_) δ 179.2 (CO), 153.3 (C-7), 143.7 (C-5), 143.5 (C-3), 143.4 (C-19), 143.4 (C-15), 141.9 (C-11), 139.3 (C-1), 138.7 (C-9), 129.2 (C-17), 128.7 (C-21), 128.4 (C-16), 127.2 (C-20), 127.1 (C-18), 126.2 (C-22), 123.8 (C-12), 117.4 (C-14), 113.7 (C-10), 106.0 (C-8), 99.9 (C-4), 98.9 (C-2). Anal. Calc. for C_25_H_15_Cl_2_NO (M = 416.30): C, 72.13; H, 3.63; N, 3.36; found: C, 72.09; H, 3.67; N, 3.31.

*6,8-Dimethyl-1,3-diphenylacridin-9(10H)-one* (**3g**). Prepared from compound **2g** (189 mg, 0.5 mmol). Yield: 150 mg, (0.40 mmol, 80%); brown solid. Mp: 286–287 °C. IR ν_max_ (film): 3021, 2962, 1593, 1534 cm^−1^. ^1^H-NMR (250 MHz, DMSO-*d*_6_) δ 11.42 (s, 1H), 7.76 (d, *J* = 7.2 Hz, 2H), 7.66 (s, 1H), 7.58–7.42 (m, 3H), 7.34 (m, 5H), 7.10 (s, 2H), 6.75 (s, 1H), 2.60 (s, 3H), 2.37 (s, 3H). ^13^C-NMR (63 MHz, DMSO-*d*_6_) δ 178.7 (CO), 143.8 (C-5), 143.3 (C-7), 143.0 (C-9), 142.3 (C-3), 142.1 (C-19), 142.1 (C-11), 139.9 (C-15), 139.0 (C-1), 129.2 (C.12), 128.5 (C-17), 128.5 (C-21), 127.3 (C-16), 127.1 (C-20), 126.1 (C-18), 125.4 (C-22), 123.0 (C-14), 118.6 (C-10), 118.2 (C-4), 114.3 (C-2), 113.7 (C-8), 23.2 (C9-**Me**), 21.3 (C11-**Me**). Anal. Calc. for C_27_H_21_NO (M = 375.46): C, 86.37; H, 5.64; N, 3.73; found: C, 86.32; H, 5.68; N, 3.72.

*1-(4-Nitrophenyl)-3-phenylacridin-9(10H)-one* (**3h**). Prepared from compound **1h** (441 mg, 1.0 mmol). Yield: 338 mg, (0.86 mmol, 86 % overall) without the need for a separate oxidation step; pink solid. Mp: 174–175 °C. IR ν_max_ (film): 3401, 3325, 3260, 1587, 1493 cm^−1^. ^1^H-NMR (250 MHz, DMSO-*d*_6_) δ 8.30 (s, 1H), 7.66 (d, *J* = 7.0 Hz, 2H), 7.46 (t, *J* = 7.3 Hz, 1H), 7.38 (d, *J* = 8.6 Hz, 3H), 7.31–7.23 (m, 1H), 7.22–7.12 (m, 5H), 6.84 (t, *J* = 7.2 Hz, 1H), 6.65 (d, *J* = 8.5 Hz, 2H). ^13^C-NMR (63 MHz, DMSO-*d*_6_) δ 182.3 (CO), 148.6 (C-18), 144.3 (C-15), 143.4 (C-5), 142.5 (C-3), 141.8 (C-7), 140.9 (C-19), 129.3 (C21), 128.9 (C-1), 127.7 (C-9), 127.5 (C-16), 127.4 (C-20 and 22), 126.8 (C-11 and C14), 119.9 (C-17), 117.1 (C-10), 116.0 (C-12), 114.2 (C-8), 112.9 (C-4), 112.5 (C_2). Anal. Calc. for C_25_H_16_N_2_O_3_ (M = 392.41): C, 76.52; H, 4.11; N, 7.14; found: C, 76.48; H, 4.05; N, 7.18.

*3-(4-Bromophenyl)-1-phenylacridin-9(10H)-one* (**3i**). Prepared from compound **2i** (214 mg, 0.5 mmol). Yield: 179 mg, (0.42 mmol, 84%); yellow solid. Mp: 326–327 °C. IR ν_max_ (film): 3090, 2984, 1624, 1570, 1533 cm^−1^. ^1^H-NMR (250 MHz, DMSO-*d*_6_) δ 11.80 (s, 1H), 8.03 (d, *J* = 7.1 Hz, 1H), 7.81–7.63 (m, 6H), 7.52 (d, *J* = 8.2 Hz, 1H), 7.41–7.29 (m, 5H), 7.20 (t, *J* = 7.7 Hz, 1H), 7.16 (d, *J* = 1.6 Hz, 1H). ^13^C-NMR (63 MHz, DMSO-*d*_6_) δ 176.8 (CO), 144.5 (C-5), 143.3 (C-3), 143.2 (C-7), 142.5 (C-15), 140.7 (C-19), 138.3 (C-1), 133.7 (C-9), 132.5 C-20), 129.6 (C-17), 129.0 (C-16), 127.5 (C-18), 126.6 (C-11), 126.5 (C-14), 123.3 (C-22), 122.6 (C-21), 122.3 (C-10), 121.4 (C-12), 117.2 (C-8), 117.1 (C-4), 114.8 (C-2). Anal. Calc. for C_25_H_16_BrNO (M = 426.30): C, 70.43; H, 3.78; N, 3.29; found: C, 70.39; H, 3.82; N, 3.34.

*1-Phenyl-3-(thiophen-2-yl)acridin-9(10H)-one* (**3j**). Prepared from compound **2j** (178 mg, 0.5 mmol). Yield: 140 mg, (0.39 mmol, 79%); orange solid. Mp: 301–302 °C IR ν_max_ (film): 3057, 3007, 2922, 1673, 1592 cm^−1^. ^1^H-NMR (250 MHz, DMSO-*d*_6_) δ 11.76 (s, 1H), 8.01 (dd, *J* = 8.1, 1.1 Hz, 1H), 7.78–7.64 (m, 4H), 7.49 (d, *J* = 8.1 Hz, 1H), 7.40–7.27 (m, 5H), 7.25–7.14 (m, 3H). ^13^C-NMR (63 MHz, DMSO-*d*_6_) δ 176.2 (CO), 144.3 (C-5), 142.9 (C-7), 142.9 (C-19), 141.7 (C-15), 140.4 (C-3), 136.8 (C-1), 133.3 (C-9), 129.0 (C-17), 128.5 (C-22), 127.8 (C-21), 127.2 (C-16), 126.3 (C-20), 126.2 (C-18), 125.9 (C-14), 122.0 (C-11), 121.5 (C-10), 121.1 (C-12), 116.8 (C-8), 116.6 (C-4), 112.5 (C-2). Anal. Calc. for C_23_H_15_NOS (M = 353.44): C, 78.16; H, 4.28; N, 3.96; found: C, 78.12; H, 4.24; N, 4.04.

*1,3-Di(furan-2-yl)acridin-9(10H)-one* (**3k**). Prepared from compound **2k** (165 mg, 0.5 mmol). Yield: 126 mg, (0.38 mmol, 77%); brown solid. Mp: 195–196 °C. IR ν_max_ (film): 3104, 2922, 1621, 1601 cm^−1^. ^1^H-NMR (250 MHz, CDCl_3_) δ 10.10 (s, 1H), 8.33 (d, *J* = 8.0 Hz, 1H), 7.63 (d, *J* = 1.4 Hz, 1H), 7.56–7.45 (m, 3H), 7.39 (dd, *J* = 8.0, 1.4 Hz, 2H), 7.11 (t, *J* = 7.5 Hz, 1H), 6.69 (d, *J* = 3.3 Hz, 1H), 6.57 (d, *J* = 3.0 Hz, 1H), 6.47–6.38 (m, 2H). ^13^C-NMR (63 MHz, CDCl_3_) δ 177.7 (CO), 154.5 (C-15), 152.2 (C-19), 143.6 (C-5), 142.7 (C-18), 142.2 (C-22), 140.2 (C-7), 133.9 (C-3), 133.3 (C-9), 132.5 (C-1), 127.1 (C-11), 122.7 (C-14), 121.8 (C-10), 121.1 (C-12), 117.6 (C-8), 116.7 (C-4), 112.3 (C-2), 112.1 (C-17), 111.1 (C-21), 108.4 (C-16), 108.2 (C-20). Anal. Calc. for C_21_H_13_NO_3_ (M= 327.33): C, 77.05; H, 4.00; N, 4.28; found: C, 77.11; H, 4.05; N, 4.32.

*3-(4-Methoxyphenyl)-1-phenylacridin-9(10H)-one* (**3l**)**.** Prepared from compound **2l** (50 mg, 0.13 mmol); Yield: 38 mg (0.10 mmol, 74% yield); yellow solid Mp: 296 °C. IR ν_max_ (film): 3264, 3107, 2929, 2106, 1889, 1617.^.1^H-NMR (300 MHz, CDCl_3_) δ 9.18 (s, 1H), 8.36 (dd, *J* = 8.2, 1.5 Hz, 1H), 7.69–7.59 (m, 3H), 7.55 (d, *J* = 1.8 Hz, 1H), 7.50–7.41 (m, 4H), 7.41–7.33 (m, 3H), 7.22 (ddd, *J* = 8.1, 7.0, 1.0 Hz, 1H), 6.99–6.92 (m, 2H), 3.83 (s, 3H). ^13^C-NMR (75 MHz, CDCl_3_) δ 177.1 (CO), 158.8 (C-22), 145.1 (C-5), 144.9 (C-3), 144.1 (C-7), 140.2 (C-15), 139.2 (C-1), 134.8 (C-19), 133.5 (C-9), 129.7 (C-20), 129.0 (C-17), 128.6 (C-16), 127.4 (C-18), 127.1 (C-11), 125.0 (C-14), 122.1 (C-10), 122.0 (C-12), 117.0 (C-21), 116.4 (C-8), 114.2 (C-4), 113.2(C-2), 55.2 (OMe). Anal. Calc. for C_26_H_19_O_2_N C, 82.74; H, 5.07; N, 3.71. Found C, 79.83; H, 5.18; N, 3.69.

*3-(4-Chlorophenyl)-1-phenylacridin-9(10H)-one* (**3m**). Prepared from compound **2m** (30 mg, 0.08 mmol); Yield: 22 mg (0.06 mmol, 70% yield). Yellow solid Mp: 366 °C. IR ν_max_ (film):3262, 3072, 2930, 2109, 1892, 1623. ^1^H-NMR (300 MHz, DMSO-*d*_6_) δ 11.87 (s, 1H), 8.10 (dd, *J* = 8.1, 1.5 Hz, 1H), 7.86 (dd, *J* = 7.2, 1.8 Hz, 3H), 7.78 (ddd, *J* = 8.5, 6.9, 1.6 Hz, 1H), 7.61 (t, *J* = 7.2 Hz, 3H), 7.56–7.51 (m, 1H), 7.51–7.39 (m, 4H), 7.31–7.23 (m, 2H). ^13^C-NMR (75 MHz, DMSO-*d*_6_) δ 176.9 (CO), 144.0 (C-5), 143.3 (C-3), 143.1 (C-7), 142.4 (C-15), 140.9 (C-19), 139.2 (C-1), 133.8 (C-9), 131.6 (C-22), 130.9 (C-21), 129.7 (C-20), 129.2 (C-17), 127.6 (C-16), 127.5 (C-18), 126.6 (C-11), 123.5 (C-14), 122.3 (C-10), 121.6 (C-12), 117.3 (C-6), 116.9 (C.4), 115.2 (C-2). Anal. Calc. for C_25_H_16_ONCl C, 78.64; H, 4.22; N, 3.67. Found C, 76.90; H, 4.26; N, 3.67.

*3-(2,4-Dimethoxyphenyl)-1-(4-methoxyphenyl)-acridin-9(10H)-one (***3n**). Prepared from compound **2n** (30 mg, 0.07 mmol); Yield: 24 mg (0.05 mmol, 77% yield). Orange solid Mp: 281 °C. IR ν_max_ (film): 3263, 3105, 2954, 2831, 2110, 1887, 1611. ^1^H-NMR (300 MHz, CDCl_3_) δ 9.37 (s, 1H), 8.24 (dd, *J* = 8.2, 1.5 Hz, 1H), 7.57 (d, *J* = 1.7 Hz, 1H), 7.52 (ddd, *J* = 8.4, 6.9, 1.5 Hz, 1H), 7.36 (d, *J* = 8.3 Hz, 1H), 7.31–7.22 (m, 3H), 7.16–7.08 (m, 2H), 6.93–6.84 (m, 2H), 6.51–6.44 (m, 2H), 3.78 (s, 3H), 3.77 (s, 3H), 3.74 (s, 3H). ^13^C-NMR (75 MHz, CDCl_3_) δ 176.8 (CO), 161.3 (C-20), 158.8 (C-22), 157.8 (C-18), 142.9 (C-5), 142.6 (C-3), 141.9 (C-7), 140.0 (C-1), 134.9 (C-9), 133.3 (C-15), 131.5 (C-16), 129.9 (C-20), 127.5 (C-11), 127.0 (C-14 and C-19), 121.9 (C-10), 121.8 (C-12), 121.4 (C-17), 116.7 (C-8), 116.3 (C-4), 113.2 (C-2), 105.0 (C-21), 99.1 (C21′), 55.7 (OMe), 55.5 (OMe), 55.2 (OMe). Anal. Calc. for C_28_H_23_O_4_N C, 76.87; H, 5.30; N, 3.20. Found C, 74.15; H, 5.41; N, 3.15.

*1,3-Bis(4-chlorophenyl)acridin-9(10H)-one* (**3o**). Prepared from compound **2o** (30 mg, 0.07 mol); Yield: 13 mg (0.03 mmol, 43% yield). Orange solid Mp 298 °C. IR ν_max_ (film): 3264, 3114, 2922, 2105, 1619. ^1^H-NMR (300 MHz, DMSO-*d*_6_) δ 11.84 (s, 1H), 8.04 (dd, *J* = 8.2, 1.6 Hz, 1H), 7.87–7.81 (m, 2H), 7.78 (d, *J* = 1.8 Hz, 1H), 7.72 (ddd, *J* = 8.5, 6.9, 1.6 Hz, 1H), 7.63–7.58 (m, 2H), 7.54 (d, *J* = 8.3 Hz, 1H), 7.45–7.34 (m, 4H), 7.26–7.18 (m, 2H). ^13^C-NMR (75 MHz, DMSO-*d*_6_) δ 176.8 (CO), 143.3 (C-5), 142.7 (C-3), 142.4 (C-7), 140.9 (C-19), 137.9 (C-15), 134.1 (C-1), 133.9 (C-9), 131.7 (C-18), 131.6 (C-22), 130.9 (C-17), 130.1 (C-21), 129.7 (C-16), 129.4 (C-20), 127.5 (C-11), 126.7 (C-14), 123.3 (C-10), 122.4 (C-12), 117.4 (C-8), 117.0 (C-4), 115.3 (C-2). Anal. Calc. for C_25_H_15_ONCl_2_ C, 72.13; H, 3.63; N, 3.36. Found C, 71.23; H, 3.76; N, 3.53.

## 4. Conclusions

The atom-economic method described here achieves the transformation of very simple reagents and catalysts into derivatives of the 1,3-diaryl-9-acridone framework. The method allows the two-step synthesis of dihydroacridone derivatives **2** having some potential significance as fluorescent probes for oxidant species, including reactive oxygen species (ROS) and reactive nitrogen species (NOS), since the absence of the C1-C2 double bond prevents the full conjugation of their two potential fluorescent chromophores, namely the *m*-terphenyl and acridone fragments, which would be restored upon dehydrogenation. This is an aspect of the chemistry of our compounds that will be studied in the near future. On the other hand, exposure of compounds **2** to nitrobenzene under microwave irradiation allowed their dehydrogenation to the fully aromatic derivatives **3**, which have a high potential biological significance. By forming two rings, one carbon-nitrogen and three carbon-carbon bonds over two steps, our work demonstrates the high significance of multicomponent reactions followed by suitable postcondensation modifications in terms of the generation of structural diversity.

## Figures and Tables

**Table 1:** Results of the multicomponent reaction leading to dihydroterphenyl derivatives **1**.

Compound	Ar_1_	Ar_2_	R ^1^	Yield, % ^1^
**1a**	Ph	Ph	H	83
**1b**	Ph	Ph	4-NMe_2_	90
**1c**	Ph	Ph	4-F	77
**1d**	Ph	Ph	4-Cl	75
**1e**	Ph	Ph	4-Br	72
**1f**	Ph	Ph	3,5-Cl_2_	54
**1g**	Ph	Ph	3,5-Me_2_	90
**1h**	Ph	4-NO_2_C_6_H_4_	H	82
**1i**	4-BrC_6_H_4_	Ph	H	80
**1j**	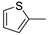	Ph	H	85
**1k**	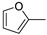	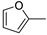	H	81
**1l**	4-MeOC_6_H_4_	Ph	H	84
**1m**	4-ClC_6_H_4_	Ph	H	76
**1n**	2,4-(MeO)_2_C_6_H_4_	4-MeOC_6_H_4_	H	69
**1o**	4-ClC_6_H_4_	4-ClC_6_H_4_	H	59

^1^ Compounds **1a**-**1k** have been previously described in reference 26.

**Table 2:** Results of the synthesis of 1,3-diaryl-1,2-dihydroacridin-9(10*H*)-one derivatives **2**.

Compound	Ar_1_	Ar_2_	R	Yield, % ^1^
**2a**	Ph	Ph	H	94
**2b**	Ph	Ph	7-NMe_2_	96
**2c**	Ph	Ph	7-F	86
**2d**	Ph	Ph	7-Cl	75
**2e**	Ph	Ph	7-Br	77
**2f**	Ph	Ph	6,8-Cl_2_	83
**2g**	Ph	Ph	6,8-Me_2_	82
**2h**	Ph	4-NO_2_C_6_H_4_	H	0 ^1^
**2i**	4-BrC_6_H_4_	Ph	H	95
**2j**	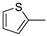	Ph	H	94
**2k**	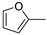	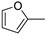	H	92
**2l**	4-MeOC_6_H_4_	Ph	H	77
**2m**	4-ClC_6_H_4_	Ph	H	86
**2n**	2,4-(MeO)_2_C_6_H_3_	4-MeOC_6_H_4_	H	85
**2o**	4-ClC_6_H_4_	4-ClC_6_H_4_	H	42

^1^ Compound **2h** was aromatized to **3h** under the reaction conditions in 86% overall yield.

**Table 3:** Results of the aromatization of compounds **2**.

Compound	Ar_1_	Ar_2_	R	Oxidant	Yield, %
**3a**	Ph	Ph	H	Pd/C	0
				MnO	0
				NBS	0
				DDQ	56
				C_6_H_5_NO_2_	82
**3b**	Ph	Ph	7-NMe_2_	C_6_H_5_NO_2_	80
**3c**	Ph	Ph	7-F	C_6_H_5_NO_2_	85
**3d**	Ph	Ph	7-Cl	C_6_H_5_NO_2_	78
**3e**	Ph	Ph	7-Br	C_6_H_5_NO_2_	80
**3f**	Ph	Ph	6,8-Cl_2_	C_6_H_5_NO_2_	78
**3g**	Ph	Ph	6,8-Me_2_	C_6_H_5_NO_2_	76
**3h**	Ph	4-NO_2_C_6_H_4_	H	C_6_H_5_NO_2_	86 ^1^
**3i**	4-BrC_6_H_4_	Ph	H	C_6_H_5_NO_2_	79
**3j**	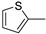	Ph	H	C_6_H_5_NO_2_	84
**3k**	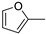	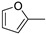	H	C_6_H_5_NO_2_	77
**3l**	4-MeOC_6_H_4_	Ph	H	C_6_H_5_NO_2_	74
**3m**	4-ClC_6_H_4_	Ph	H	C_6_H_5_NO_2_	70
**3n**	2,4-(MeO)_2_C_6_H_3_	4-MeOC_6_H_4_	H	C_6_H_5_NO_2_	77
**3o**	4-ClC_6_H_4_	4-ClC_6_H_4_	H	C_6_H_5_NO_2_	43

^1^ Compound **3h** was obtained directly from **1h**, without isolation of **2h**.

## Supplementary Materials

The following are available online, copies of NMR spectra of new compounds.
