# Supplementary material for: Sustainable Access to Acridin-9-(10H)ones with an Embedded m-Terphenyl Moiety Based on a Three-Component Reaction
Source: Molecules. 2020 Nov 27;25(23):5565. doi: 10.3390/molecules25235565 (PMC7731126; doi:10.3390/molecules25235565)
Supplement: Supplementary file 1 [file molecules-25-05565-s001.pdf]

Supporting Information

Sustainable access to acridin-9-(10*H*)-ones with an embedded mterphenyl moiety based on a three- component reaction

Damiano Rocchi, Jorge Gómez-Carpintero, Juan F. González* and J. Carlos Menéndez*

Unidad de Química Orgánica y Farmacéutica, Departamento de Química en Ciencias Farmacéuticas, Facultad de Farmacia, Universidad Complutense, 28040 Madrid, Spain

Content:

1. Copies of NMR spectra of new compounds

2. Structural study of compound 2a and 3a

Ethyl 4-methoxy-5'-(phenylamino)-2',3'-dihydro-[1,1':3',1''-terphenyl]-4'-carboxylate (1l)

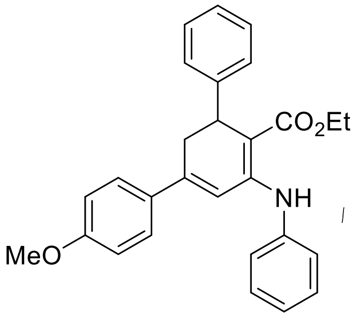

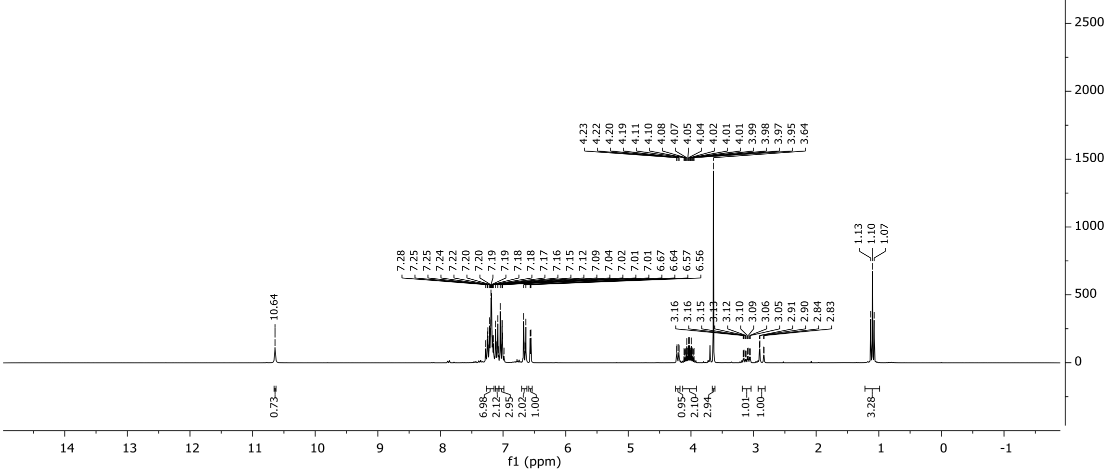

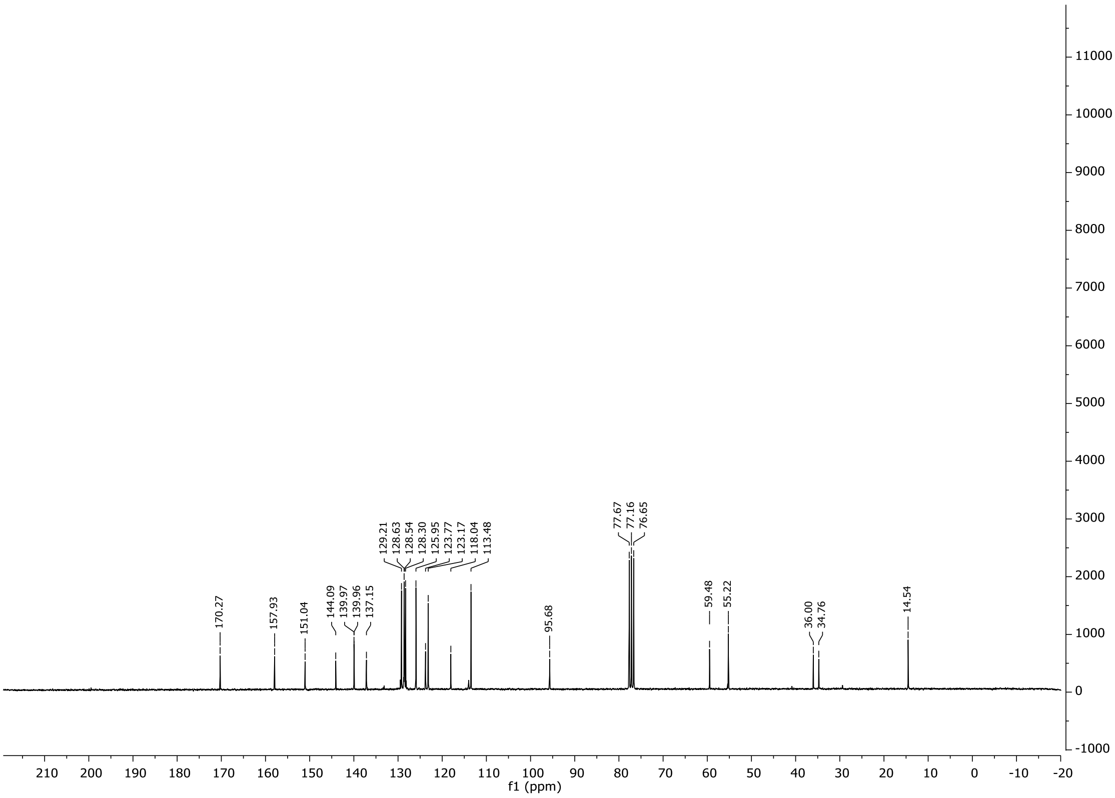

Ethyl 4-chloro-5'-(phenylamino)-2',3'-dihydro-[1,1':3',1''-terphenyl]-4'-carboxylate (1m)

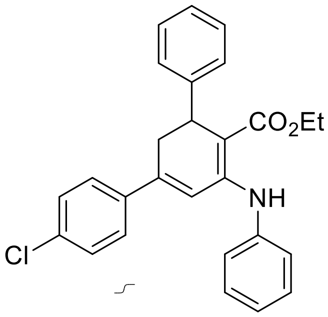

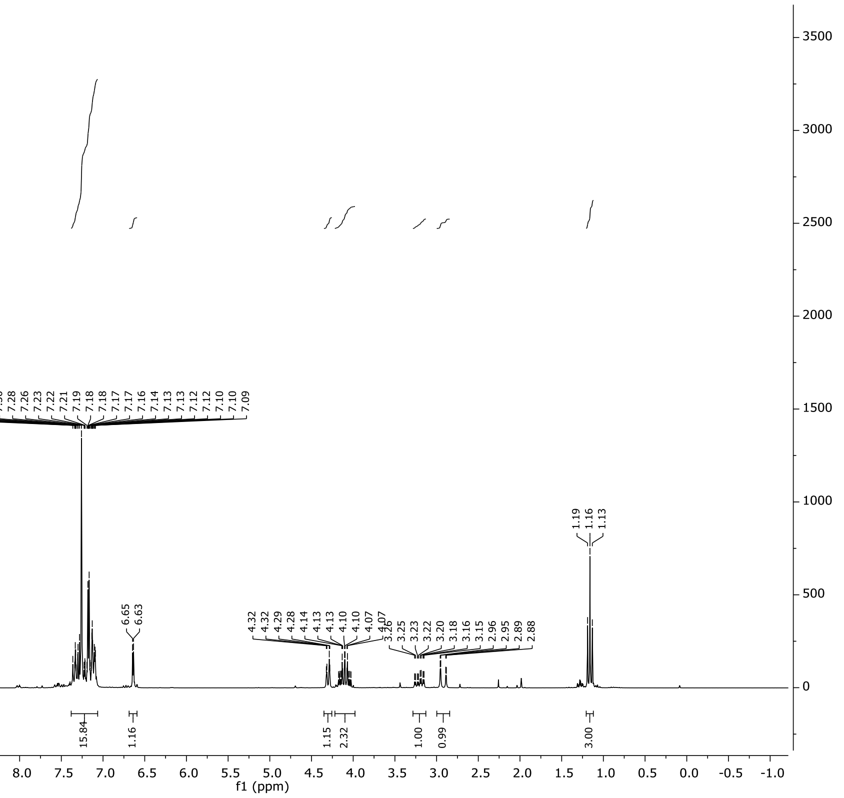

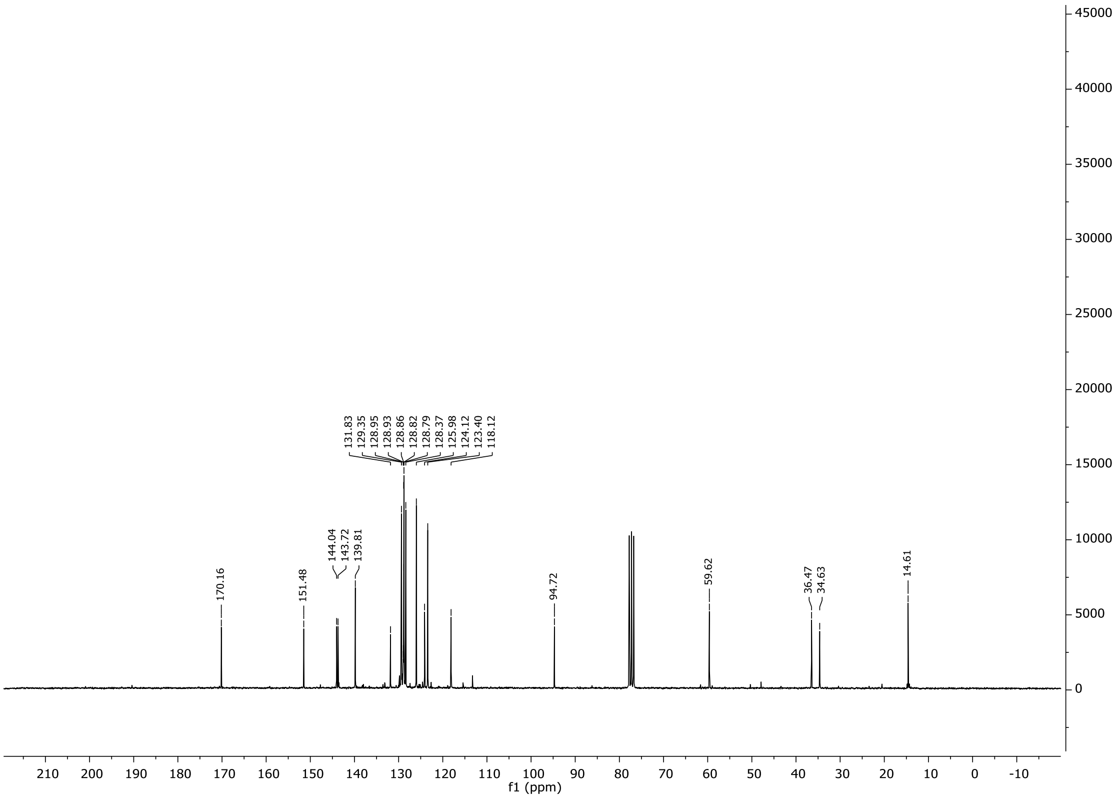

Ethyl 2,4,4''-trimethoxy-5'-(phenylamino)-2',3'-dihydro-[1,1':3',1''-terphenyl]-4'-carboxylate (1n)

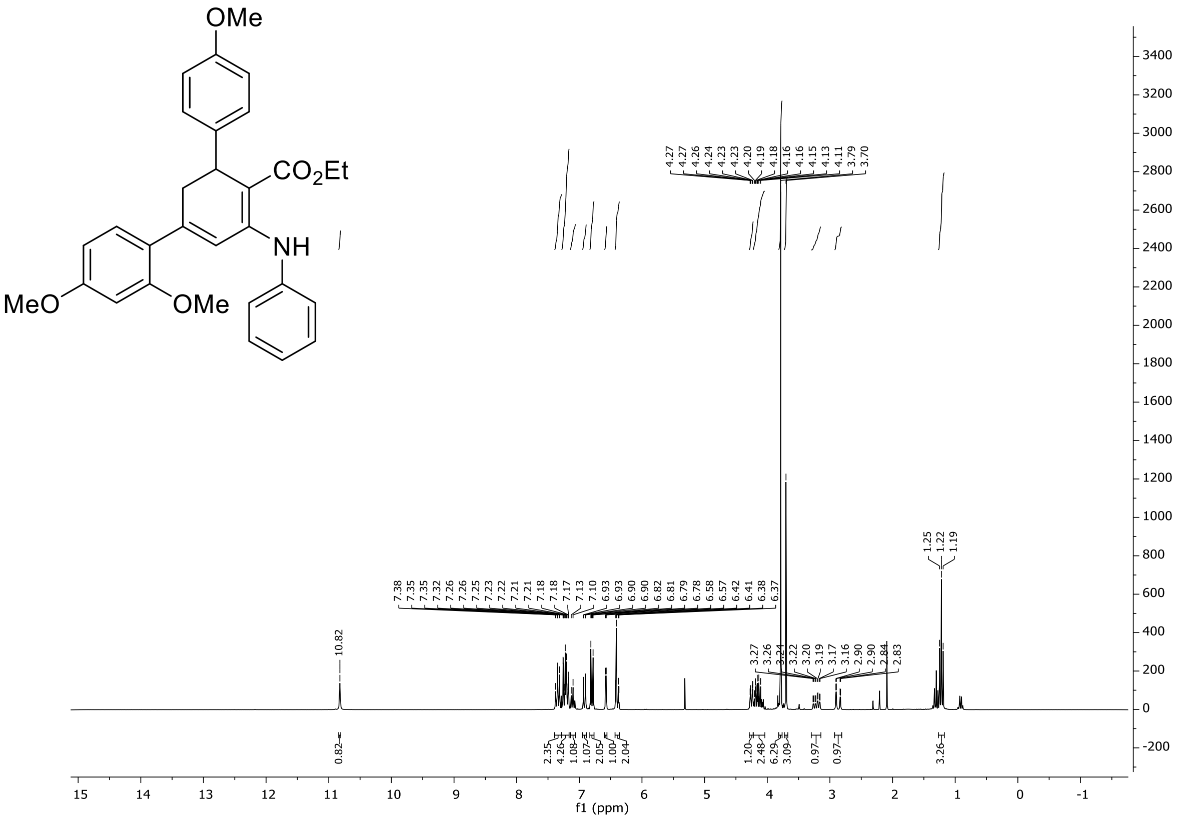

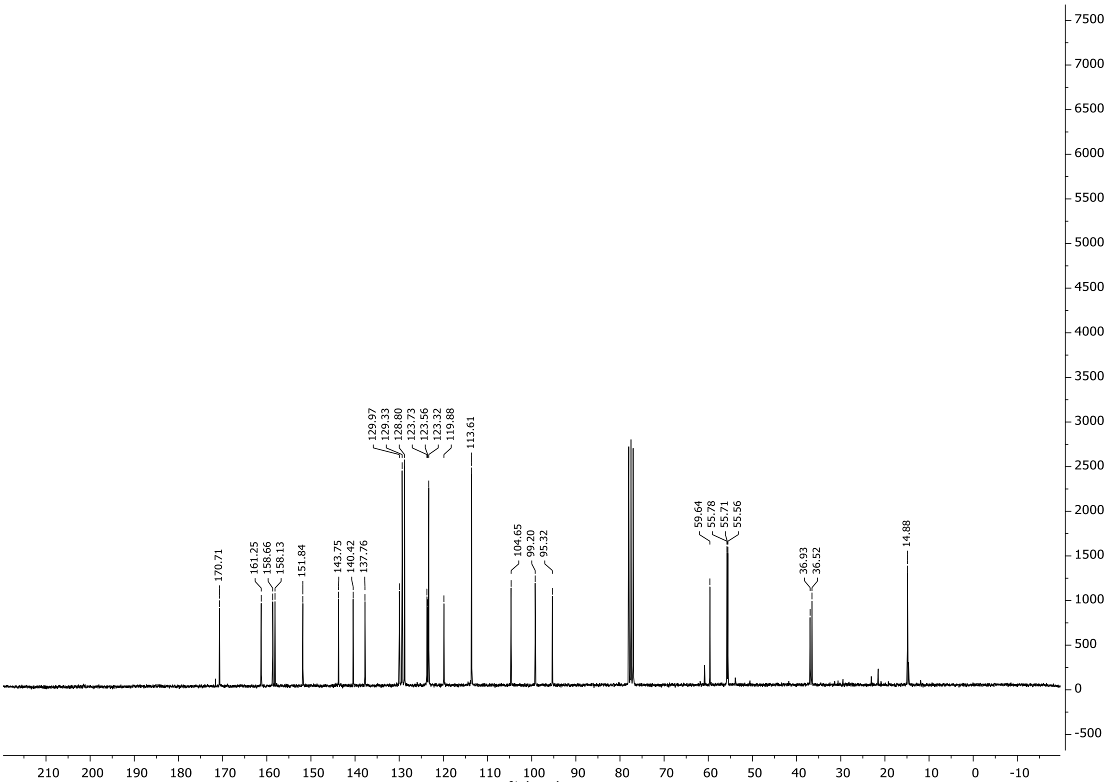

Ethyl 4,4''-dichloro-5'-(phenylamino)-2',3'-dihydro-[1,1':3',1''-terphenyl]-4'-carboxylate (1o)

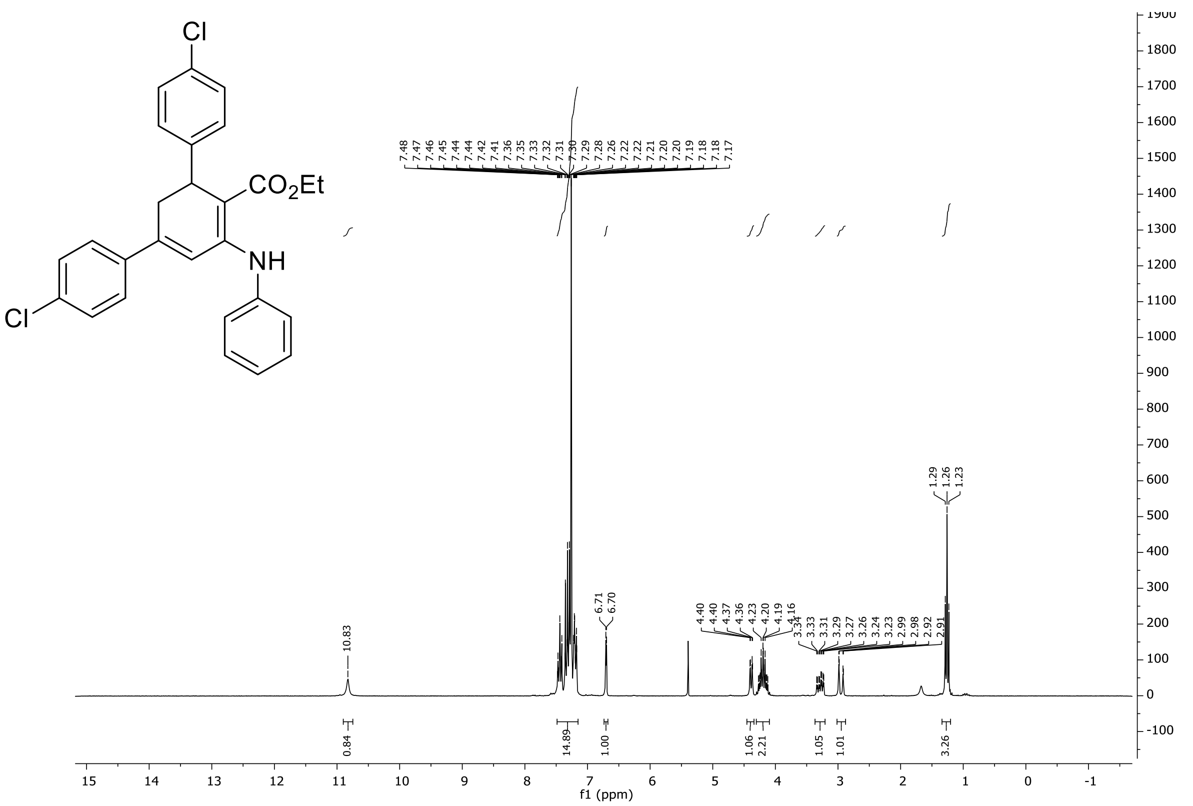

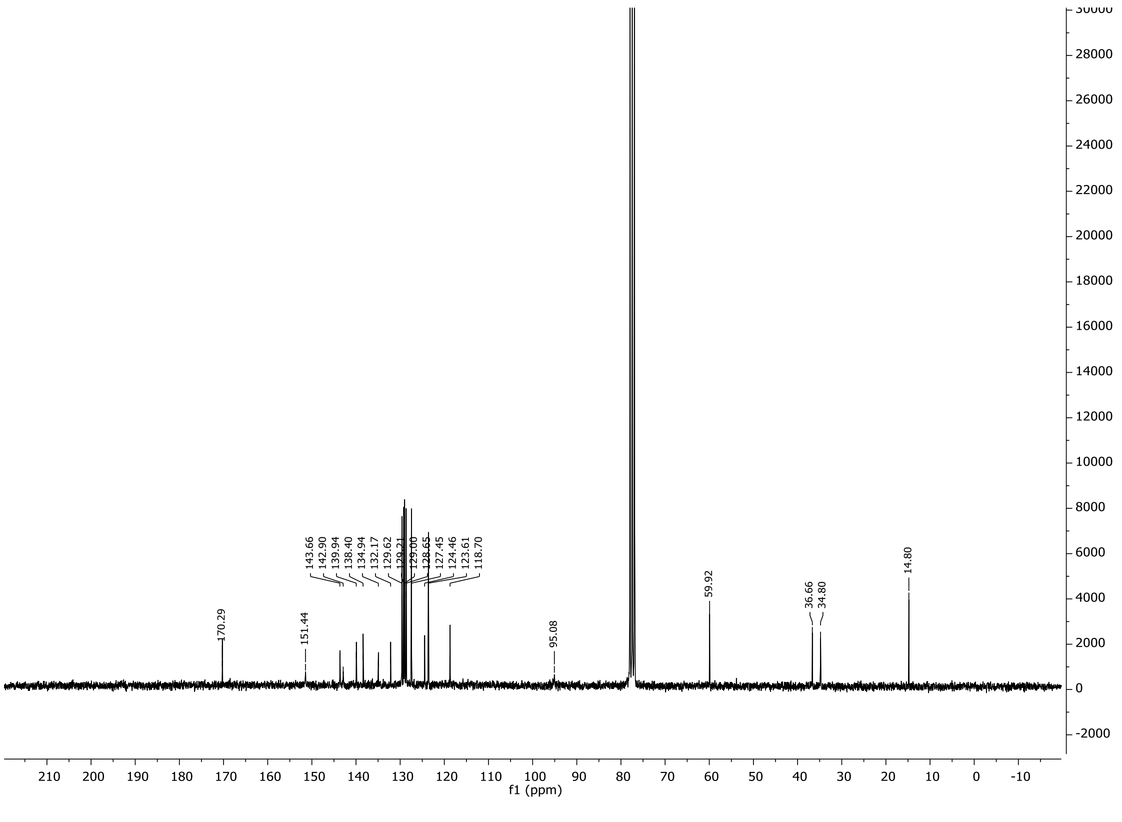

1,3-diphenyl-1,2-dihydroacridin-9(10*H*)-one (2a)

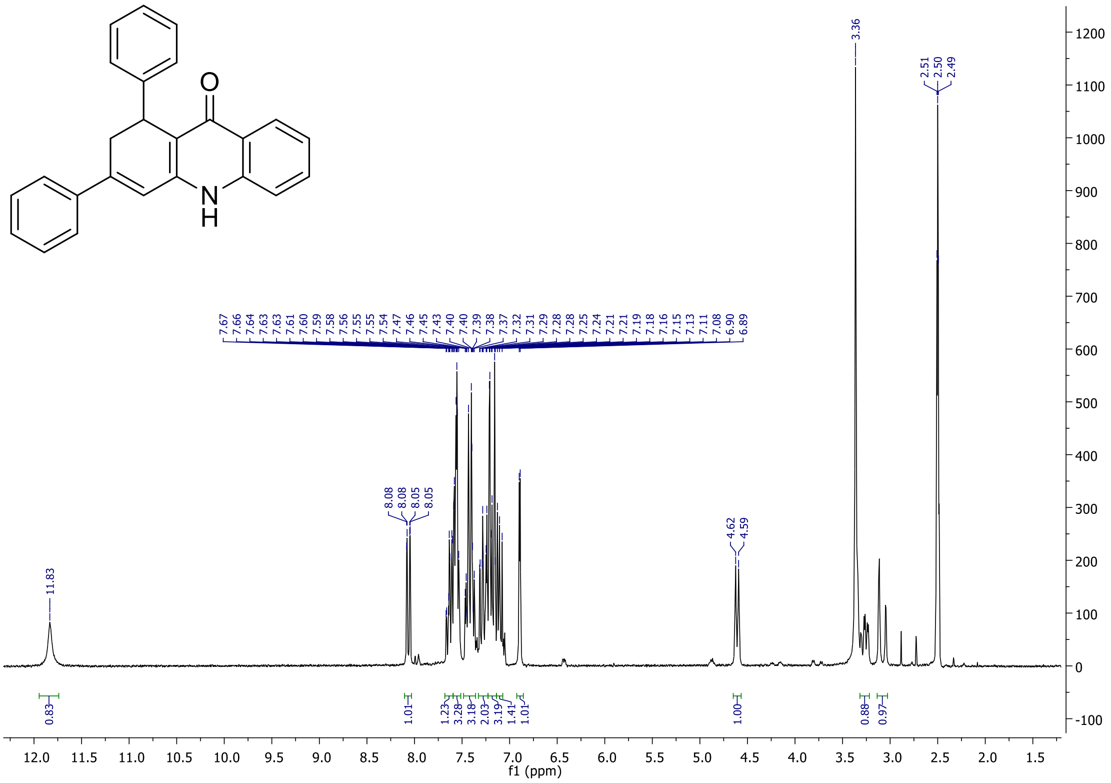

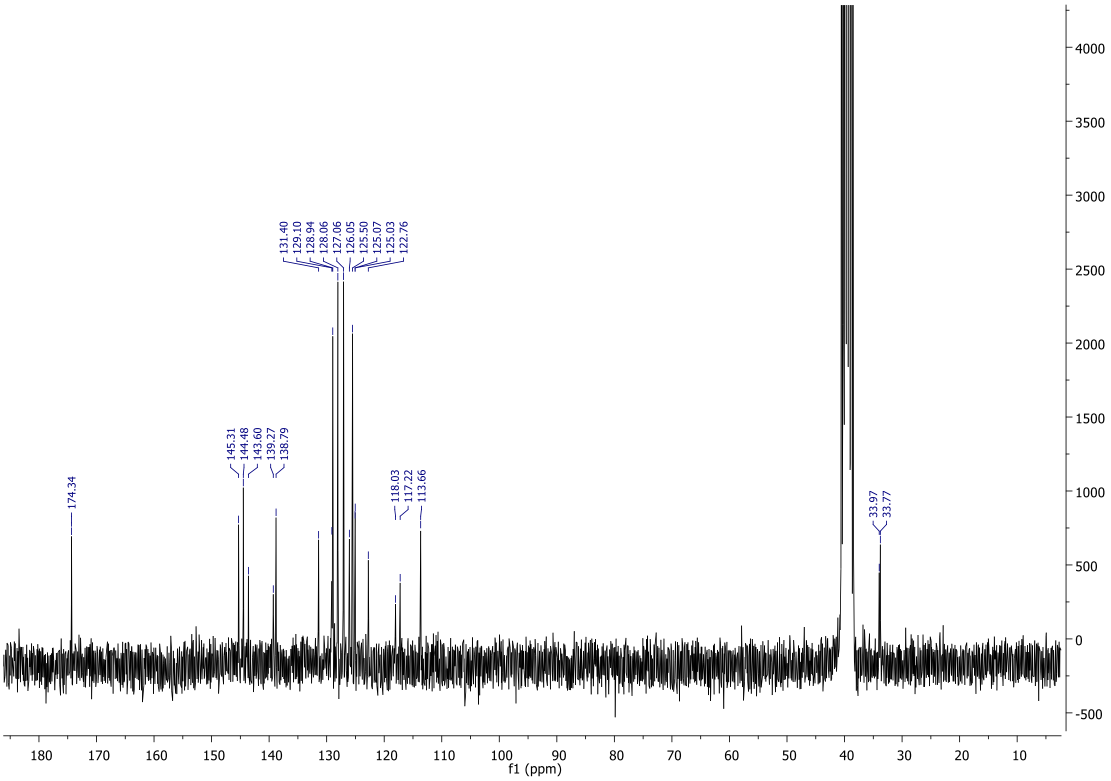

7-(dimethylamino)-1,3-diphenyl-1,2-dihydroacridin-9(10*H*)-one (2b)

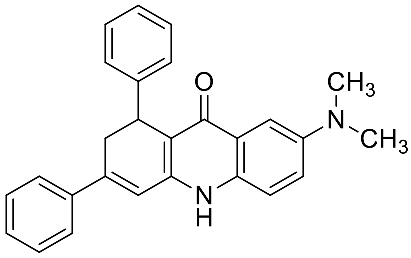

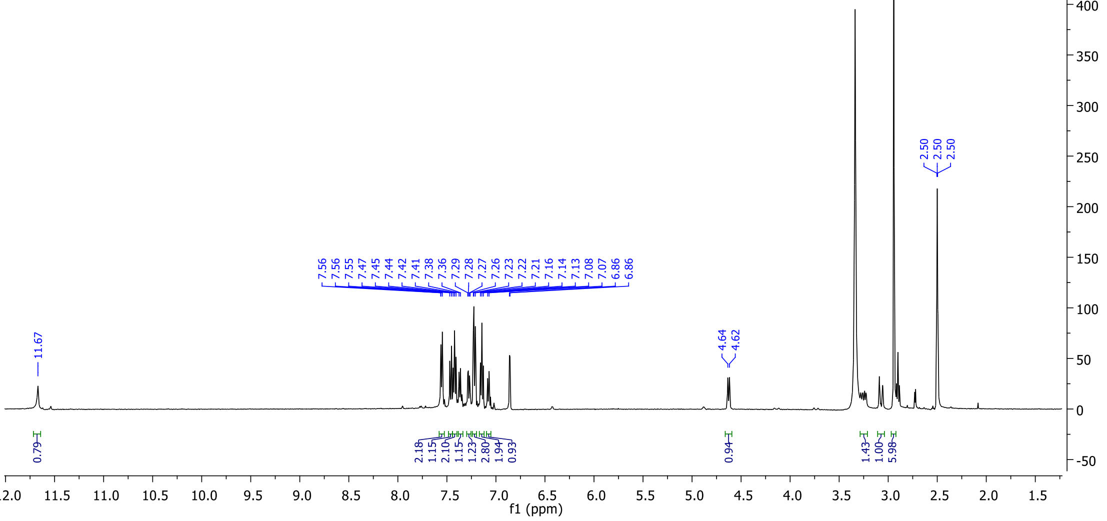

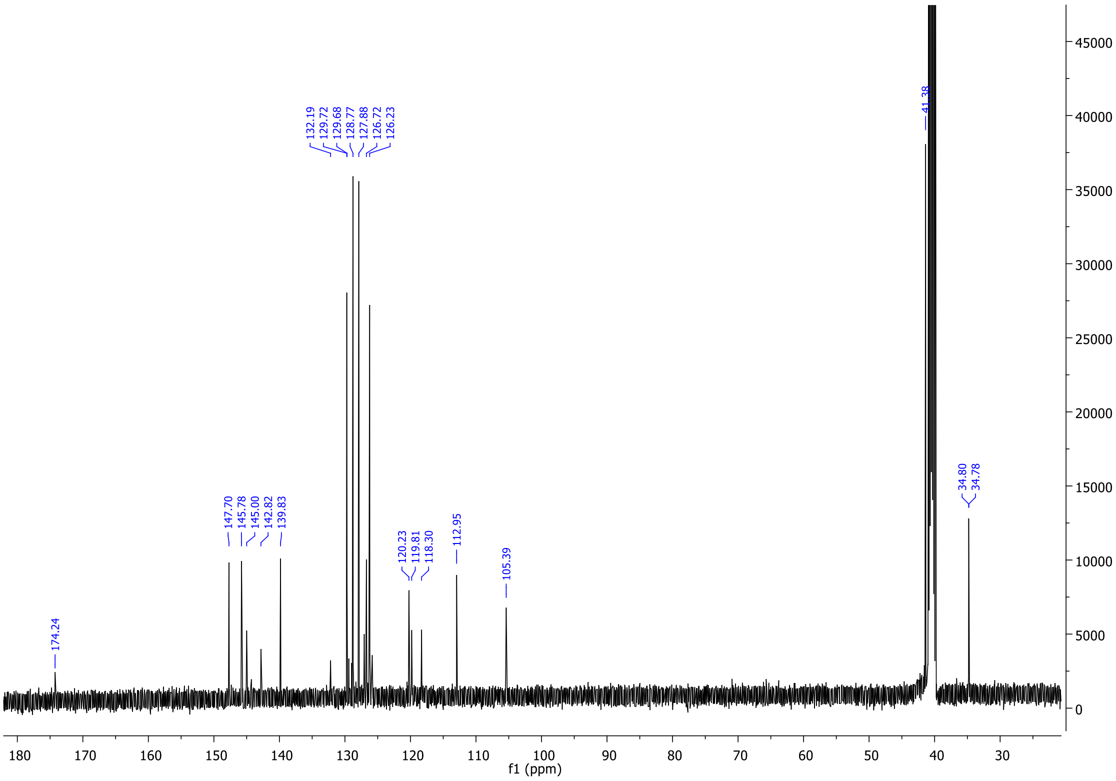

7-fluoro-1,3-diphenyl-1,2-dihydroacridin-9(10*H*)-one (2c)

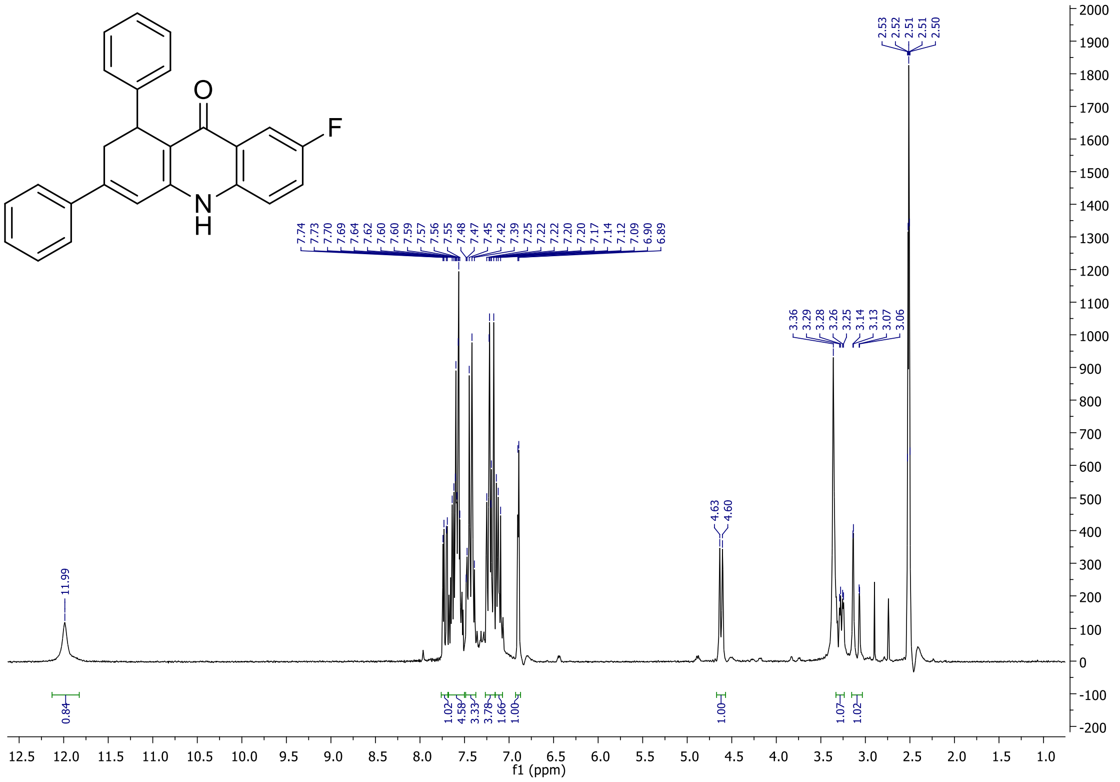

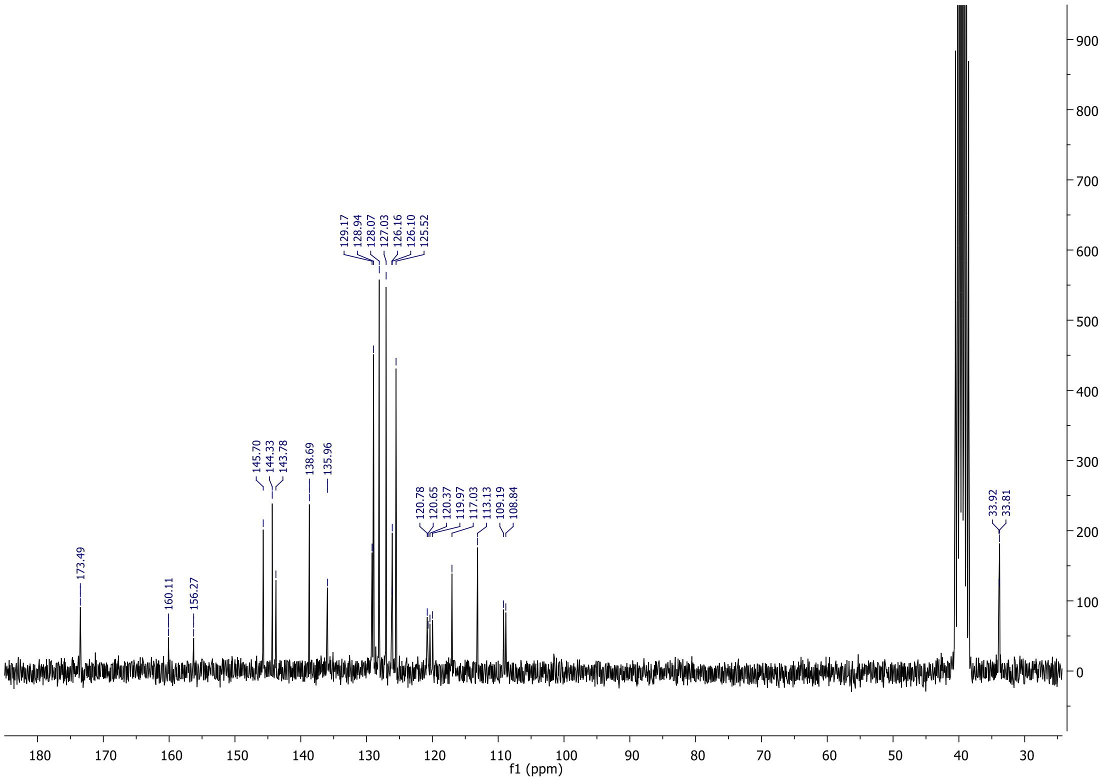

7-chloro-1,3-diphenyl-1,2-dihydroacridin-9(10*H*)-one (2d)

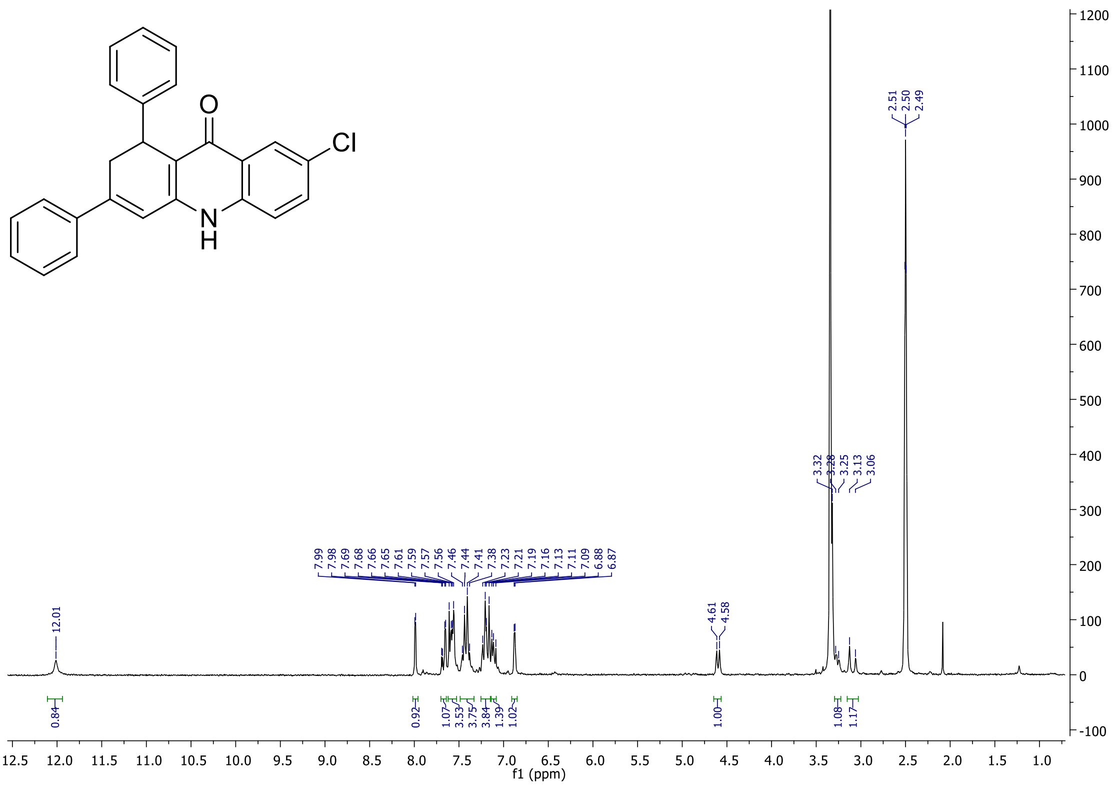

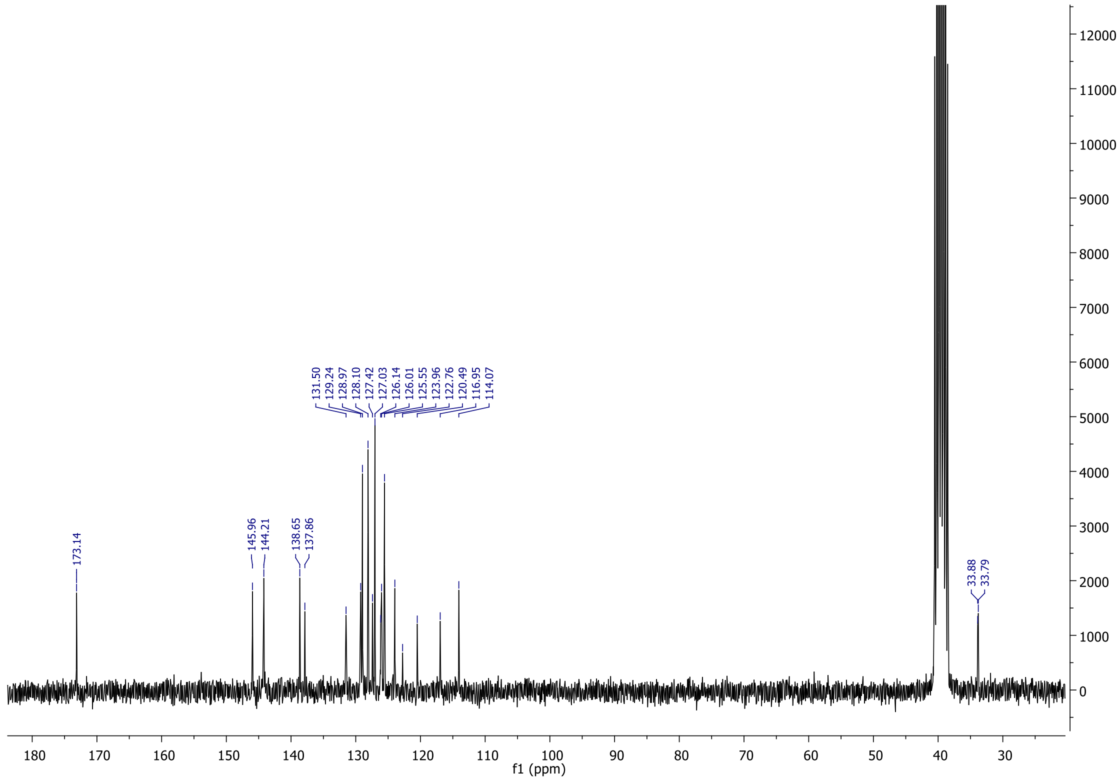

7-bromo-1,3-diphenyl-1,2-dihydroacridin-9(10*H*)-one (2e)

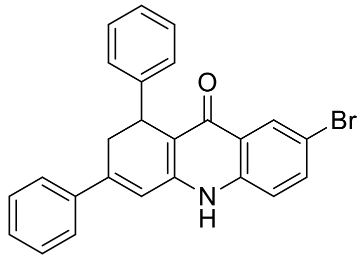

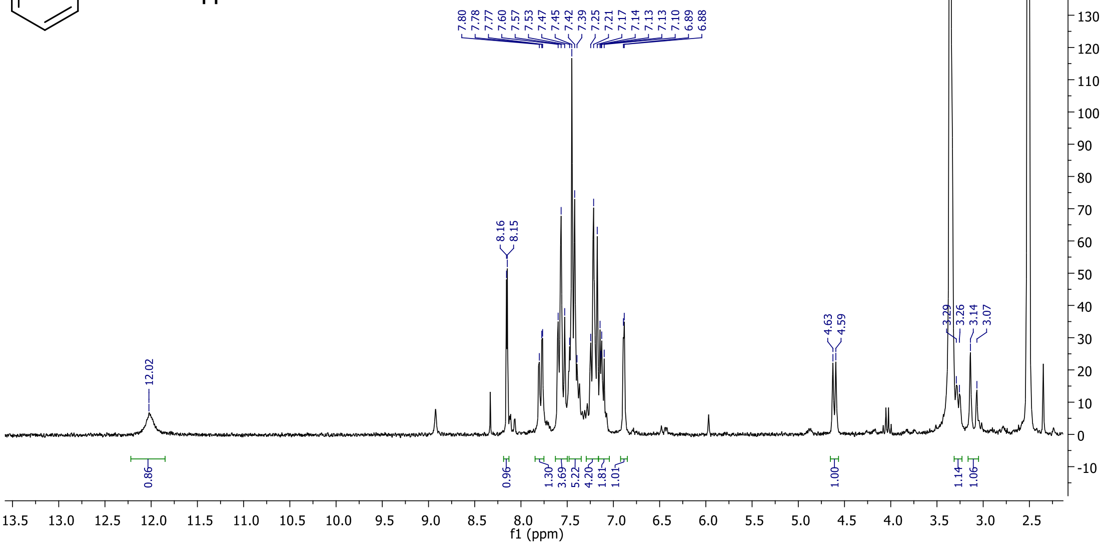

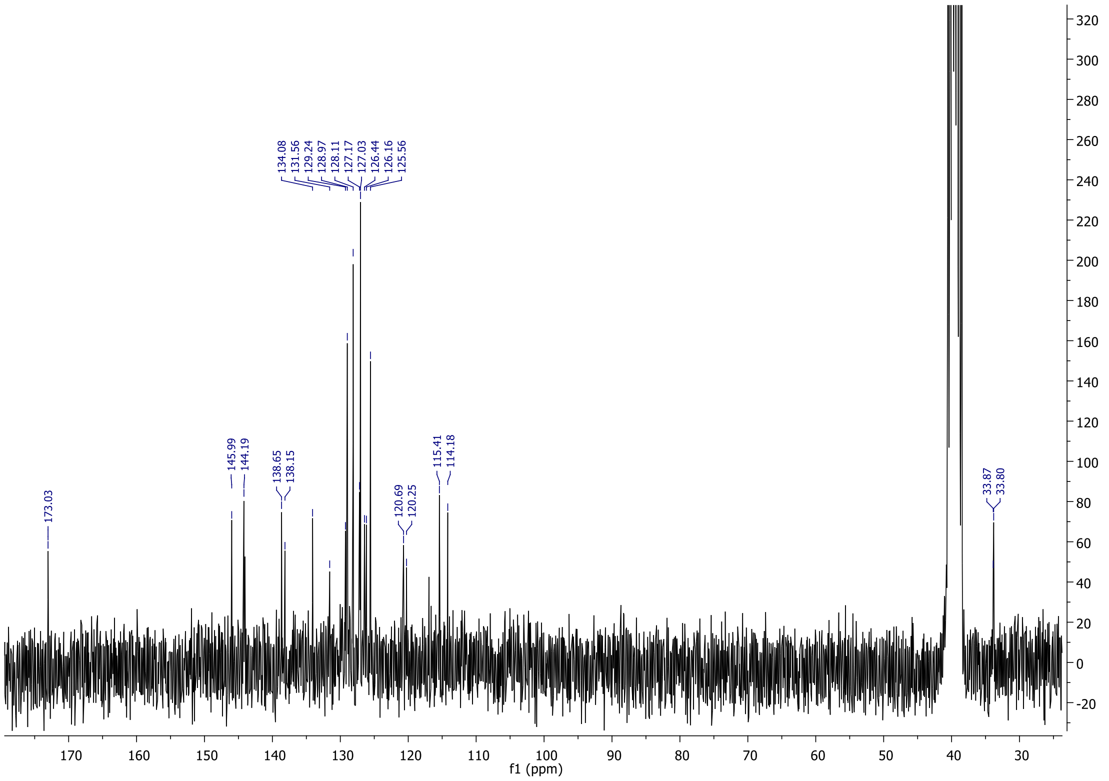

6,8-dichloro-1,3-diphenyl-1,2-dihydroacridin-9(10*H*)-one (2f)

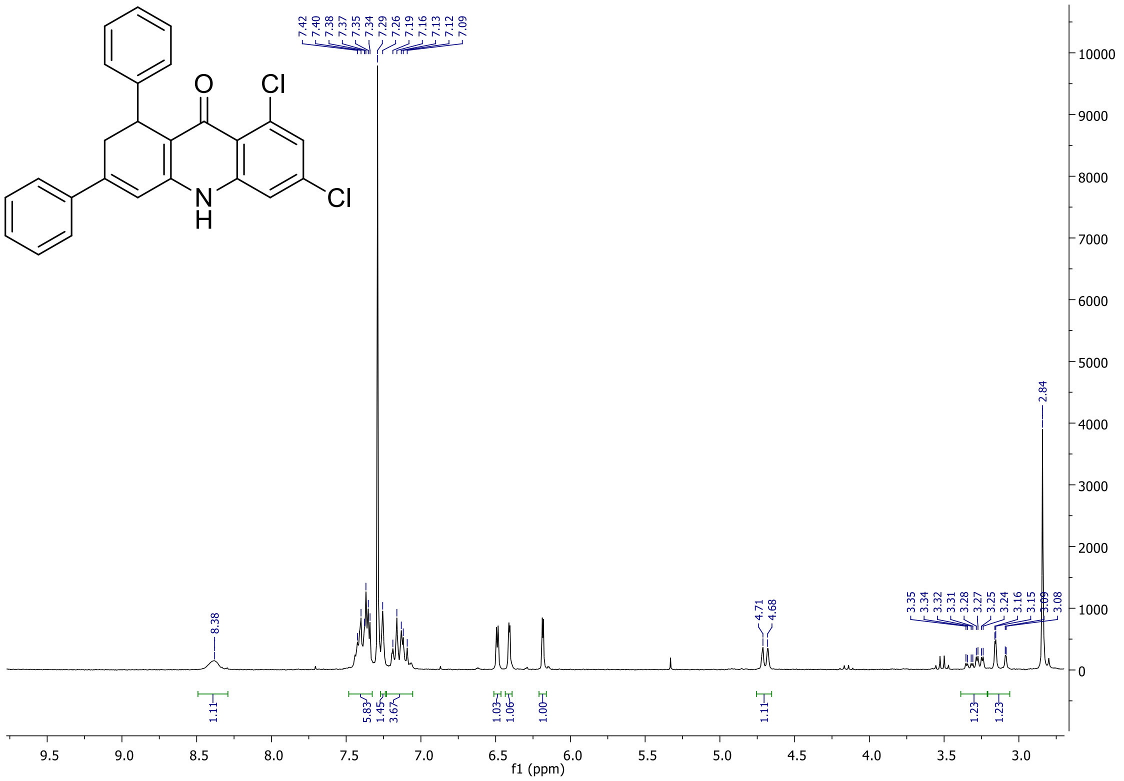

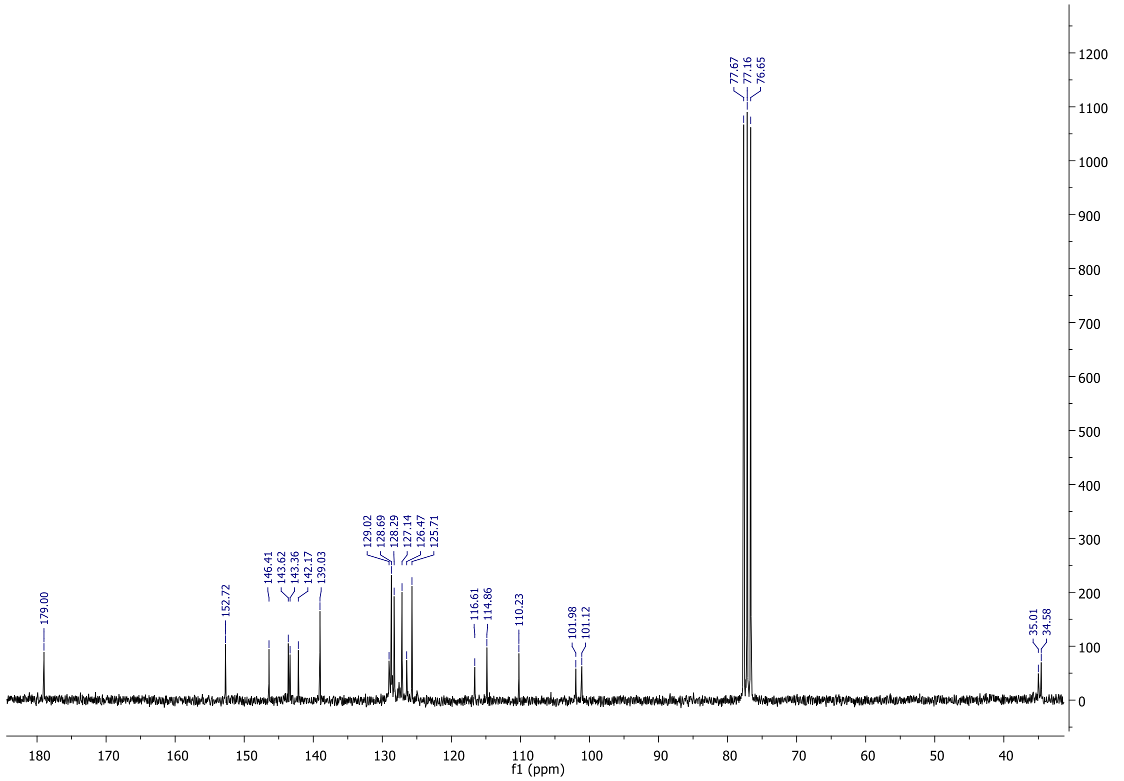

6,8-dimethyl-1,3-diphenyl-1,2-dihydroacridin-9(10*H*)-one (2g)

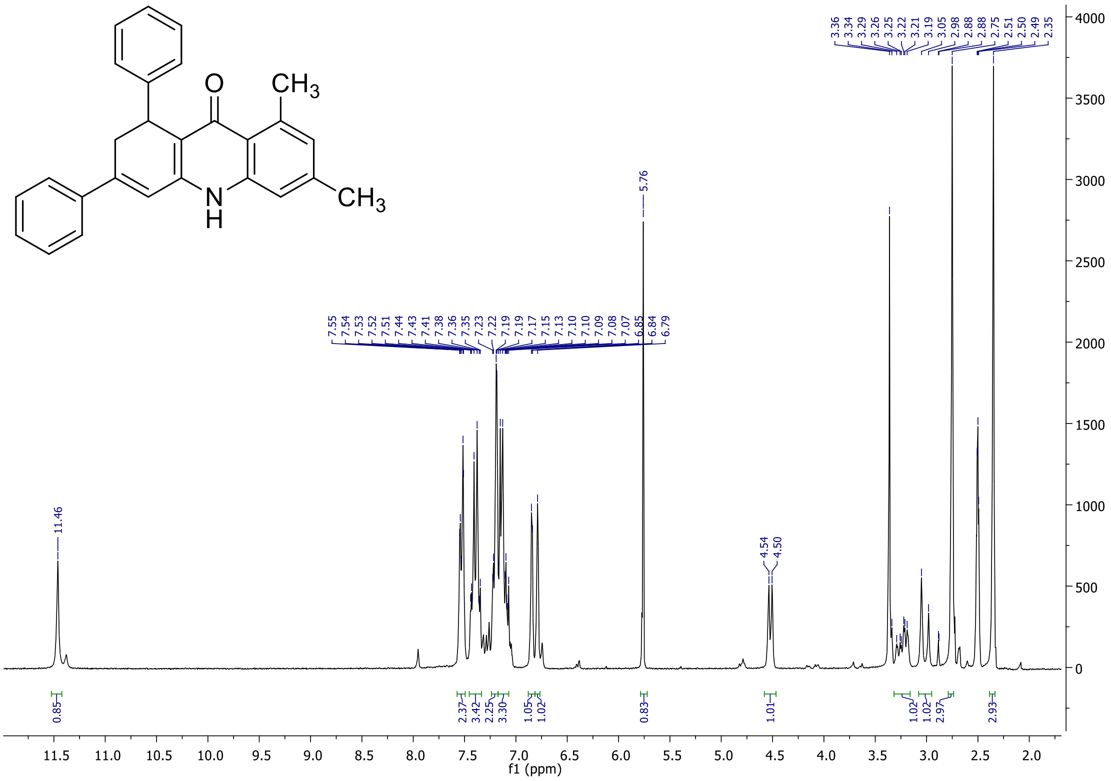

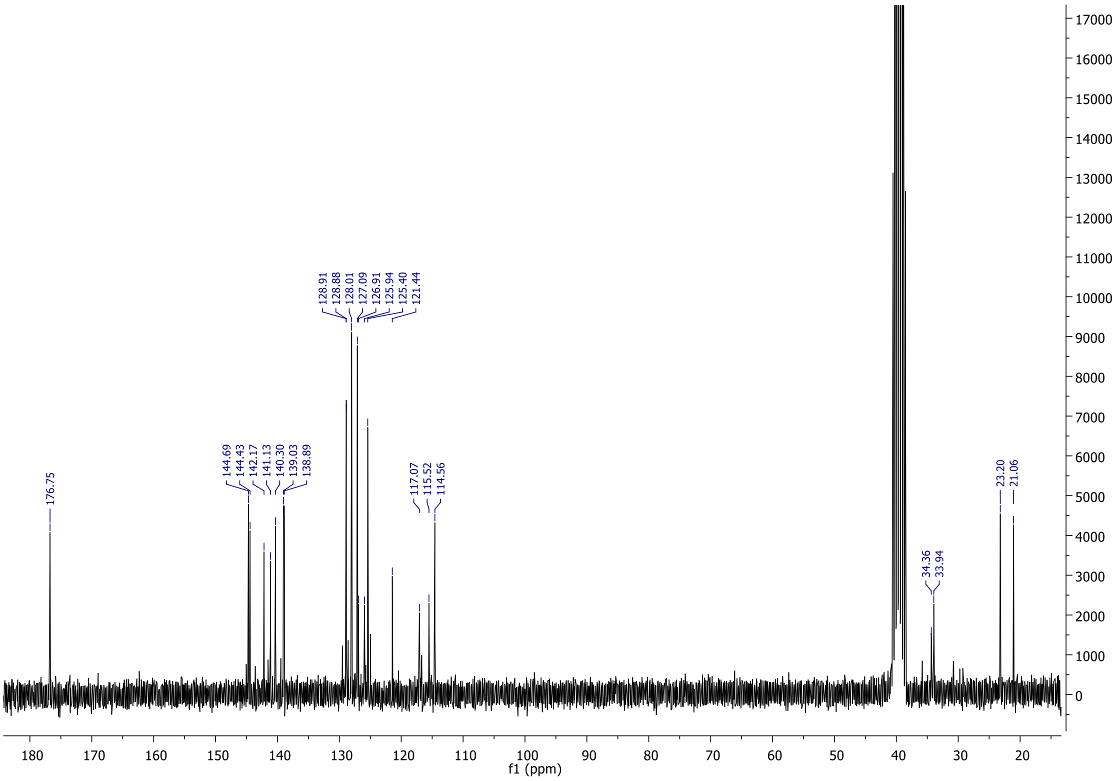

3-(4-bromophenyl)-1-phenyl-1,2-dihydroacridin-9(10*H*)-one (2i)

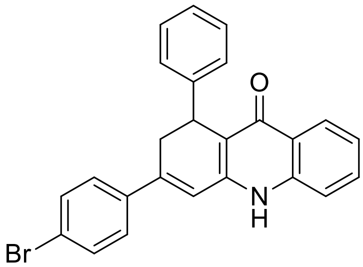

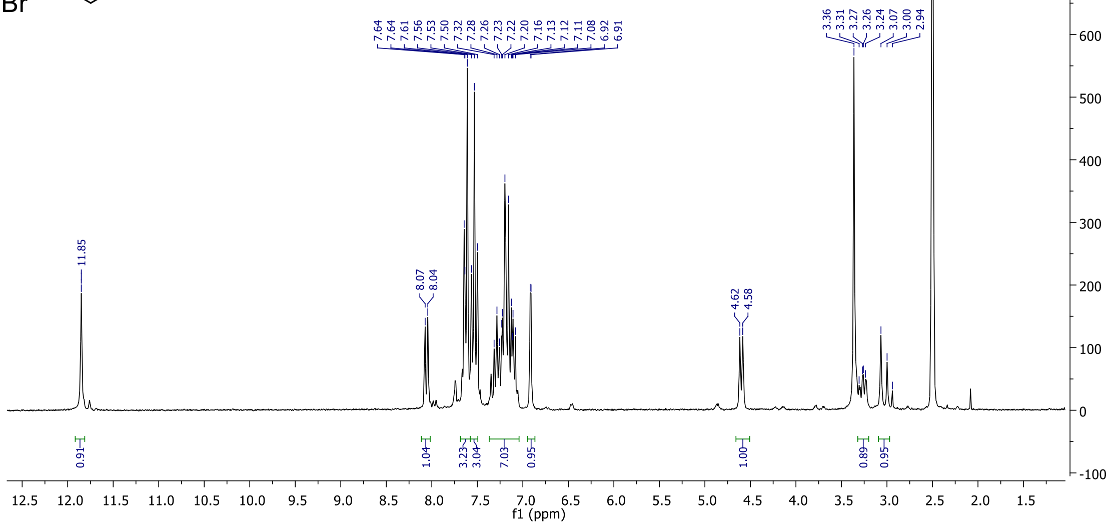

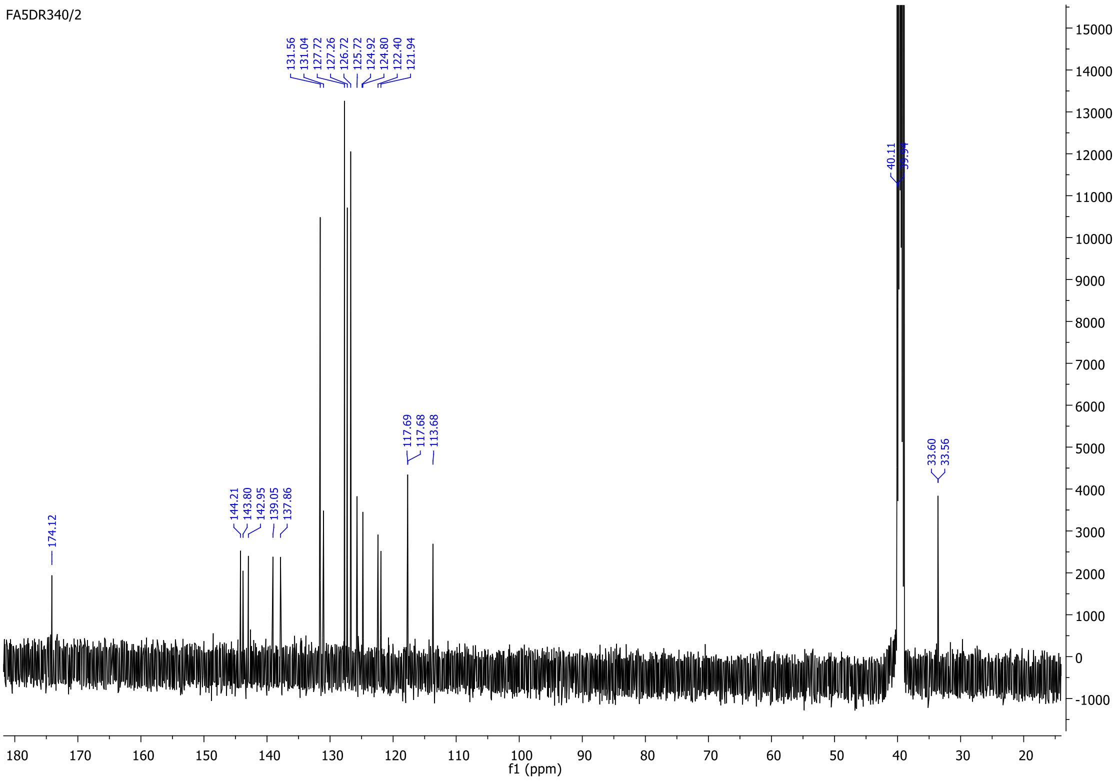

1-phenyl-3-(thiophen-2-yl)-1,2-dihydroacridin-9(10*H*)-one (2j)

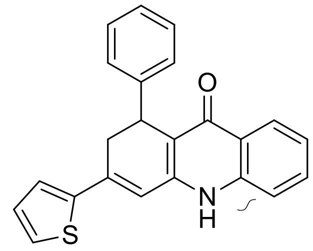

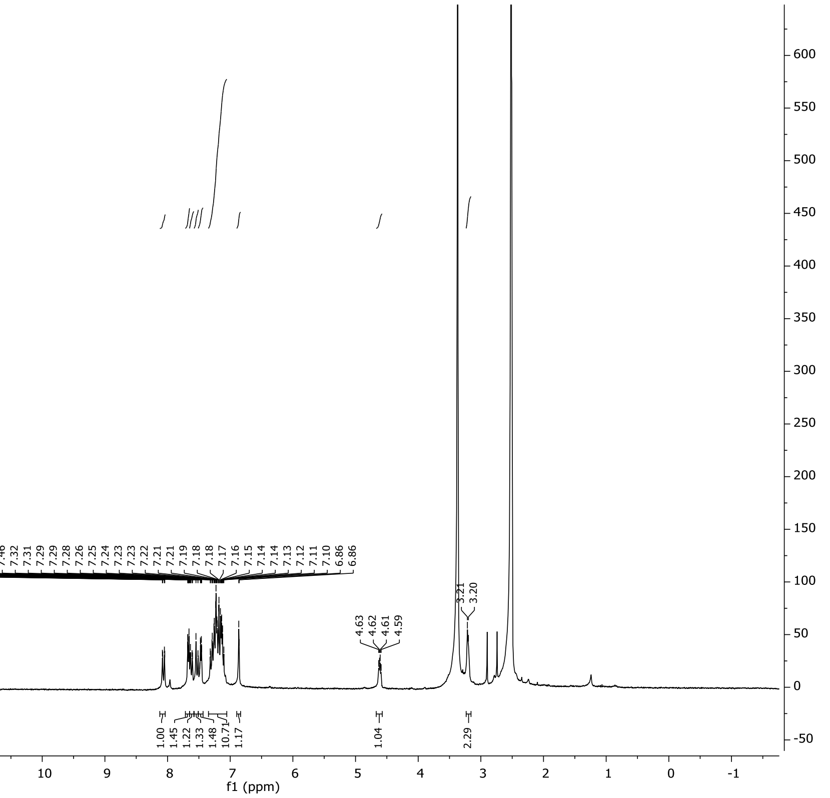

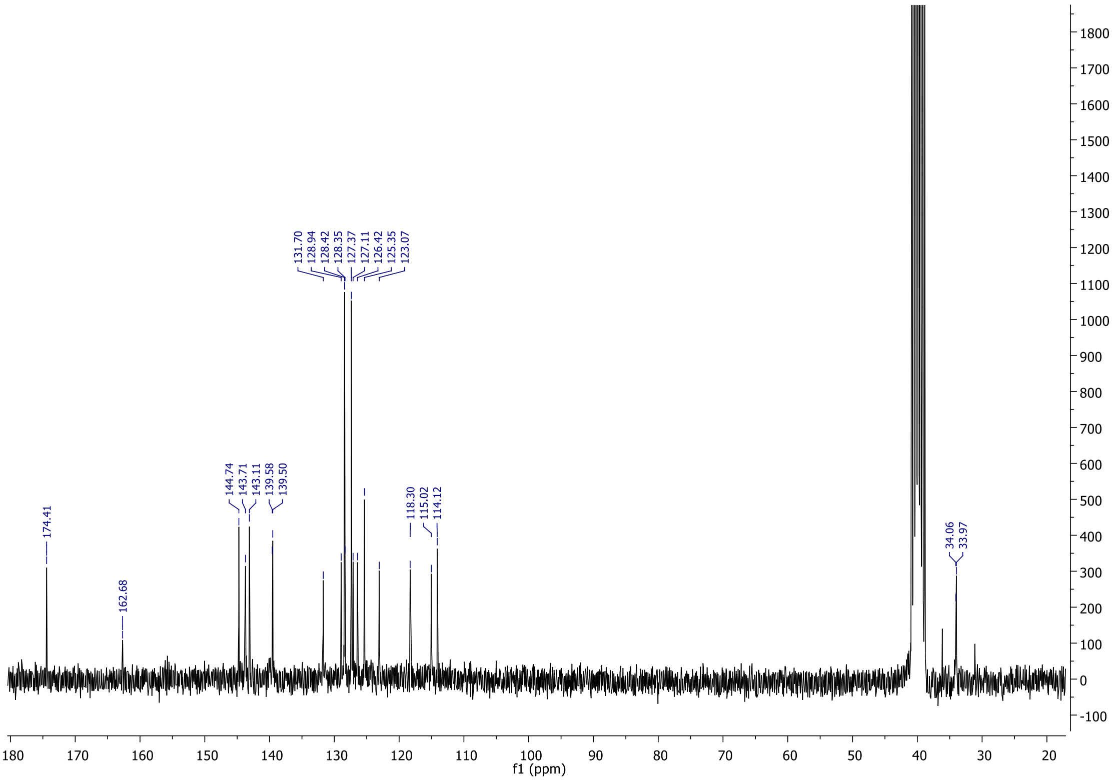

1,3-di(furan-2-yl)-1,2-dihydroacridin-9(10*H*)-one (2k)

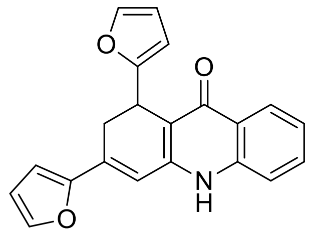

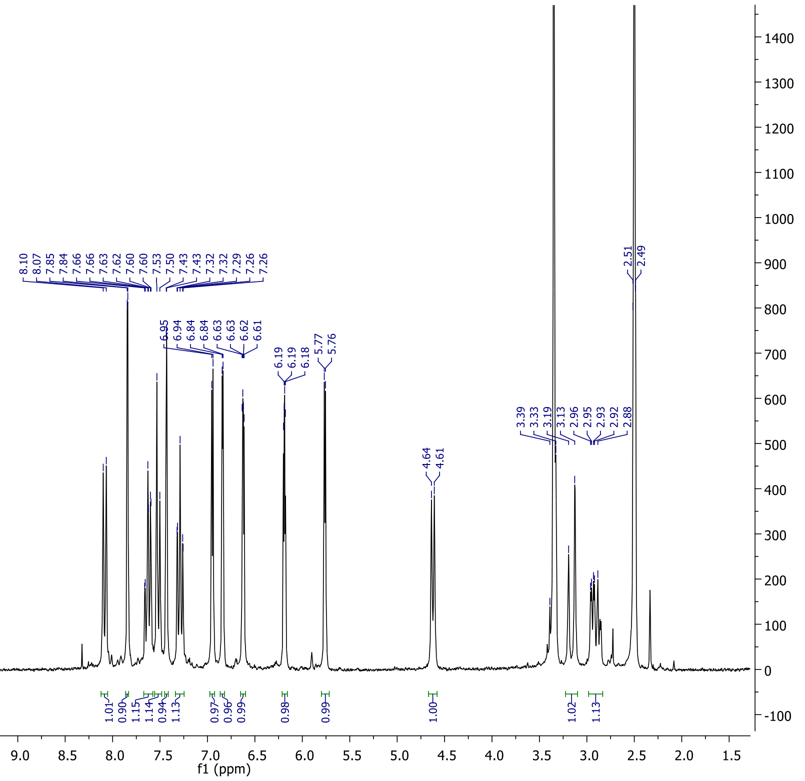

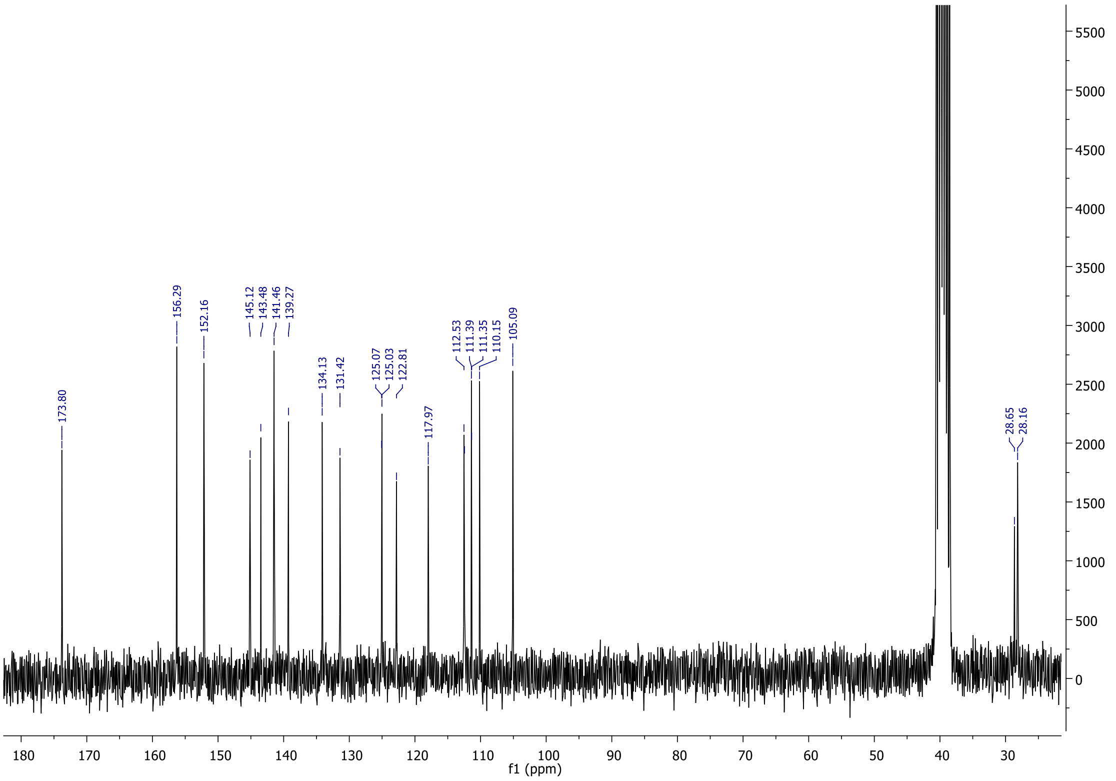

1-(4-methoxyphenyl)-3-phenyl-1,10-dihydroacridin-9(2H)-one (2l)

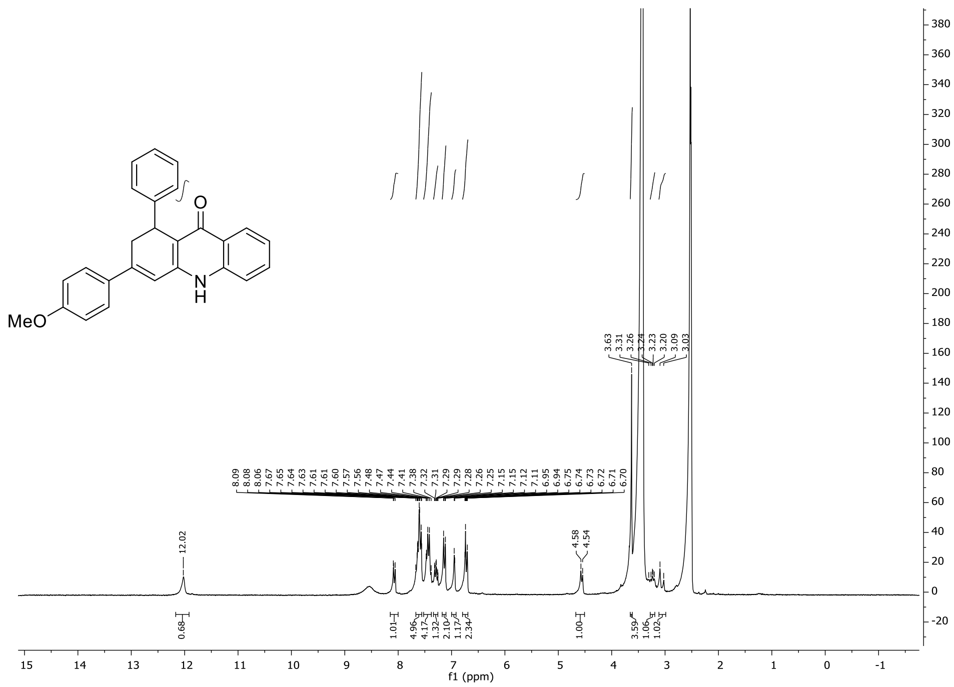

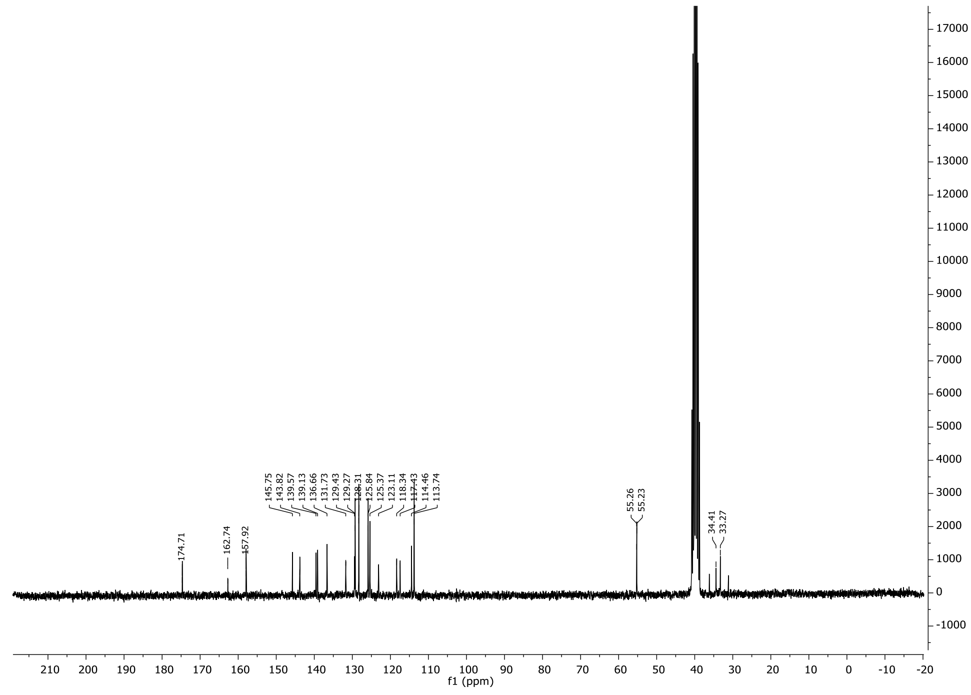

1-(4-chlorophenyl)-3-phenyl-1,10-dihydroacridin-9(2H)-one (2m)

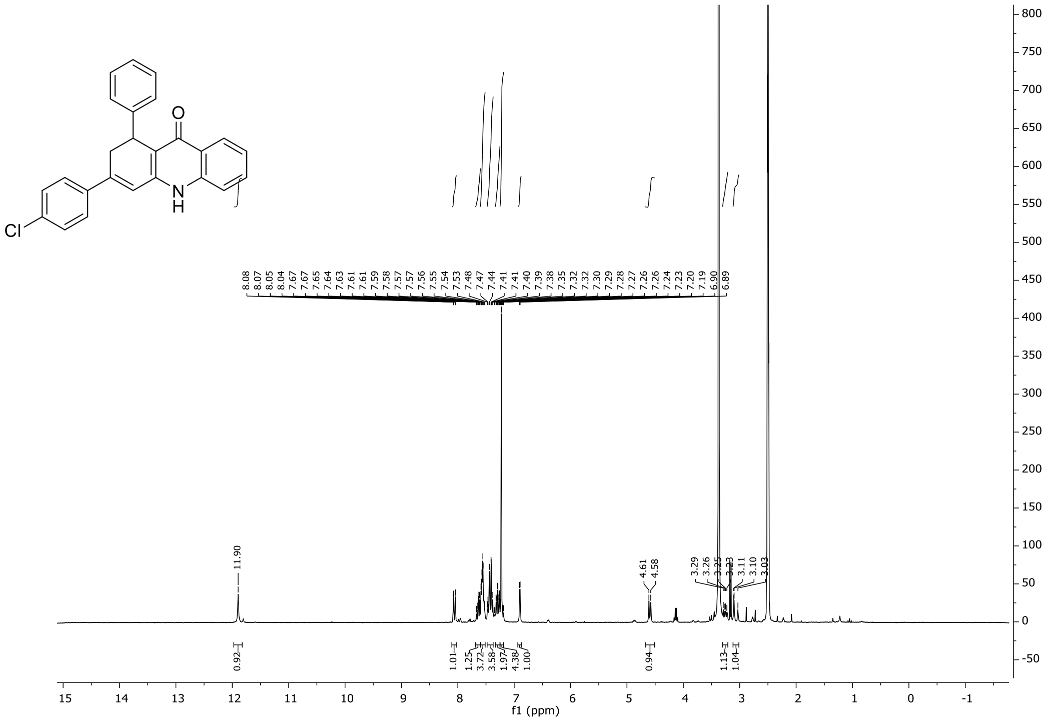

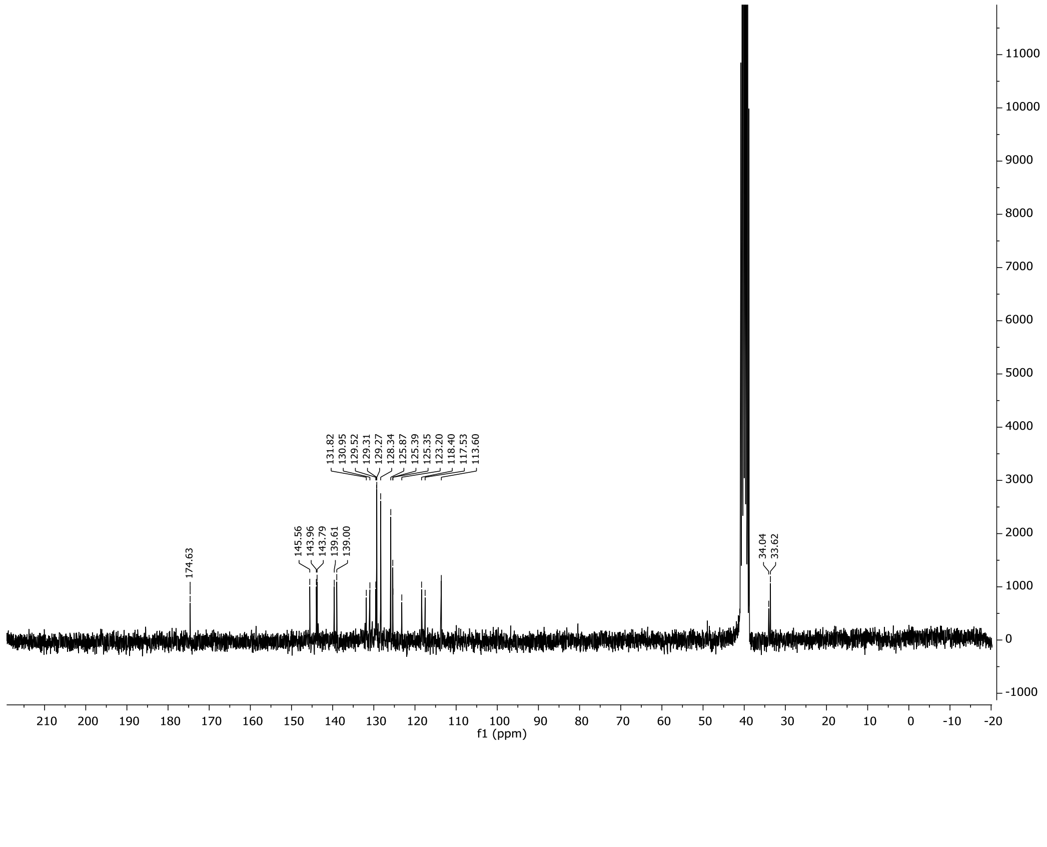

1-(2,4-dimethoxyphenyl)-3-(4-methoxyphenyl)-1,10-dihydroacridin-9(2H)-one (2n)

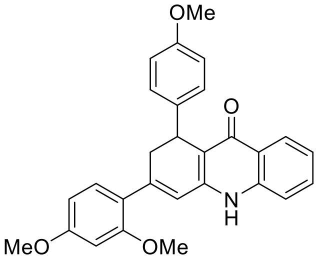

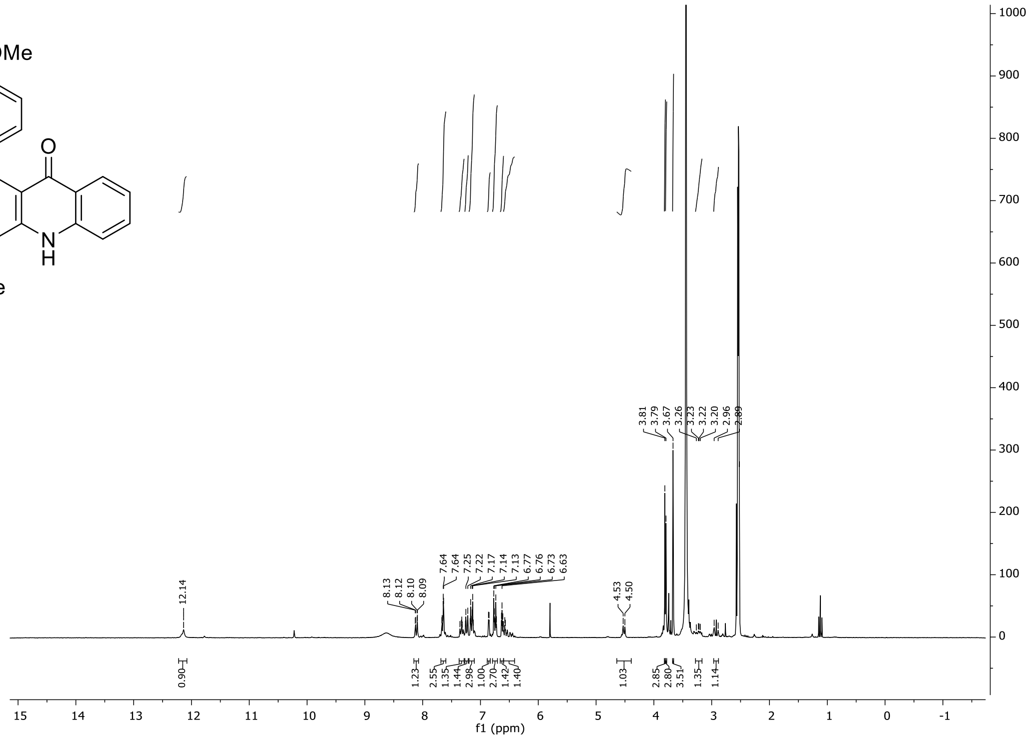

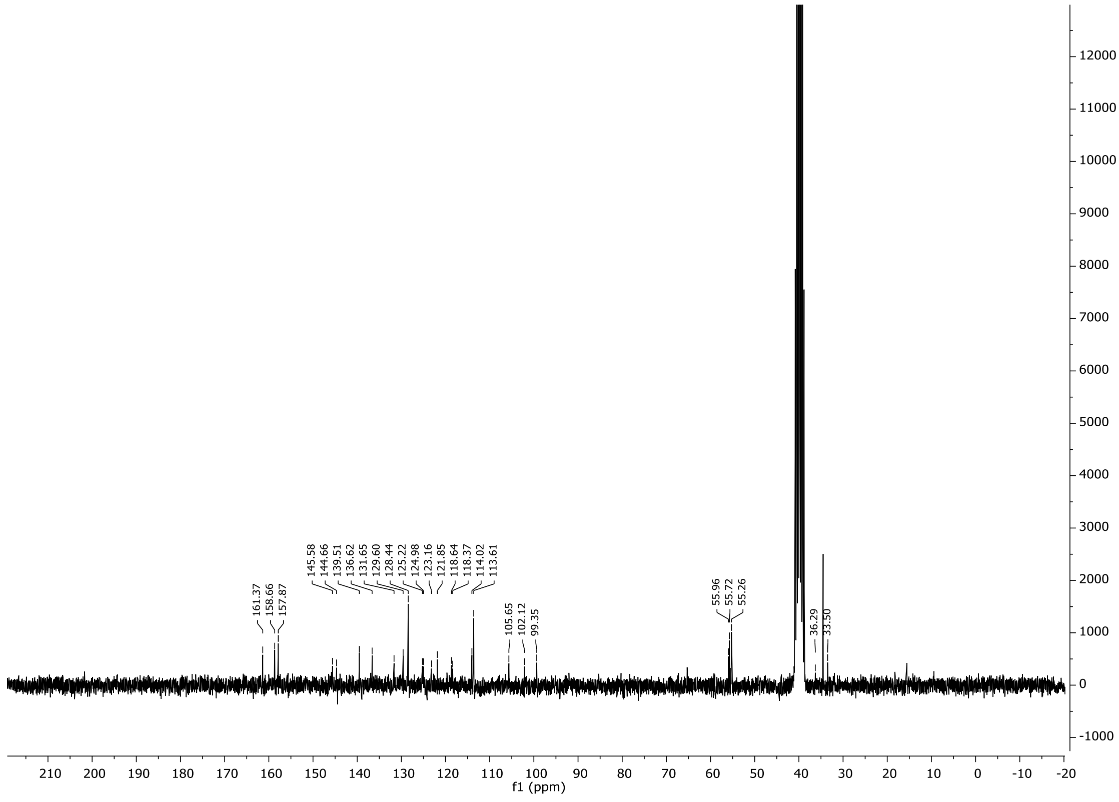

1,3-bis(4-chlorophenyl)-1,10-dihydroacridin-9(2H)-one (2o)

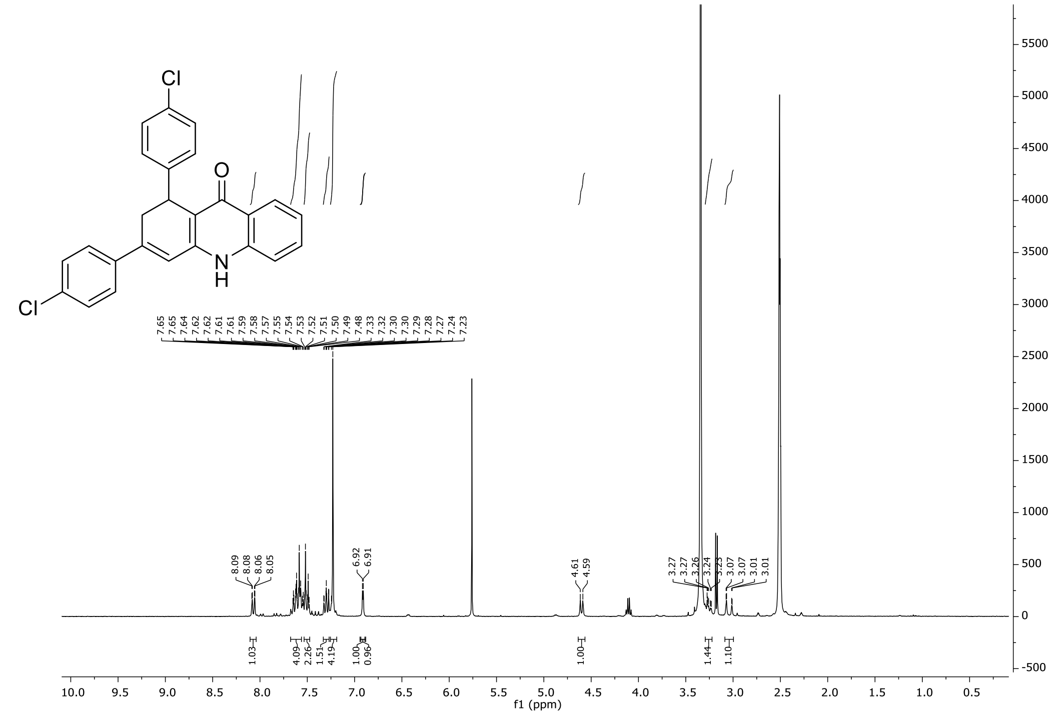

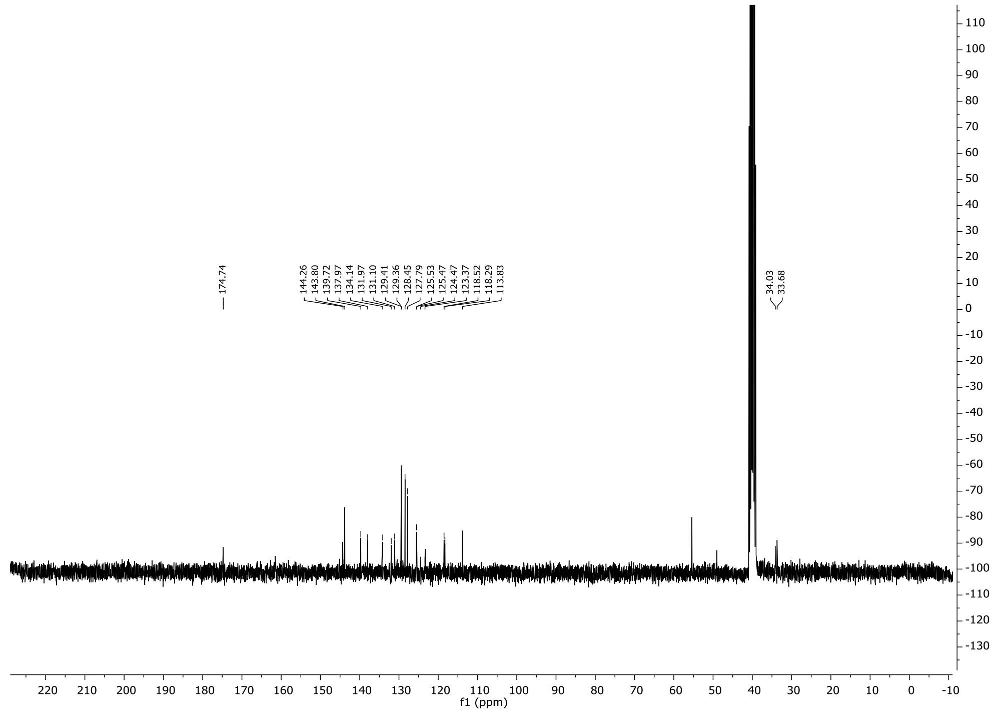

1,3-diphenylacridin-9(10*H*)-one (3a)

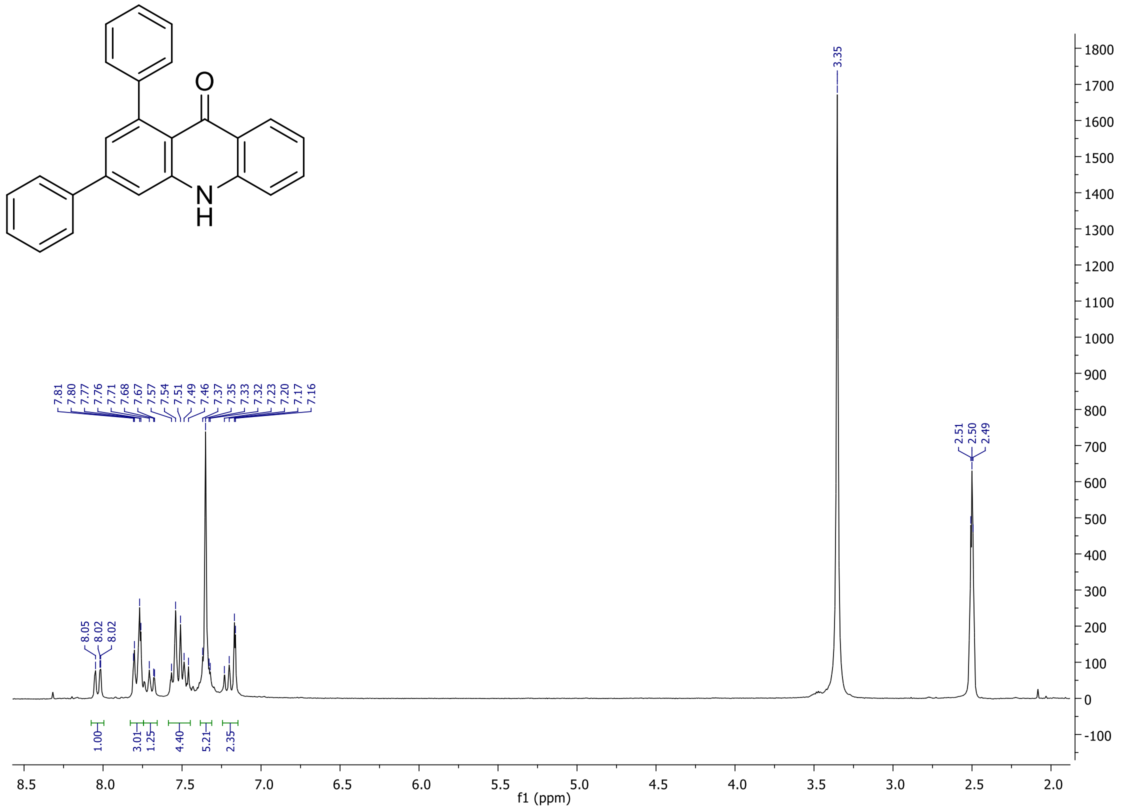

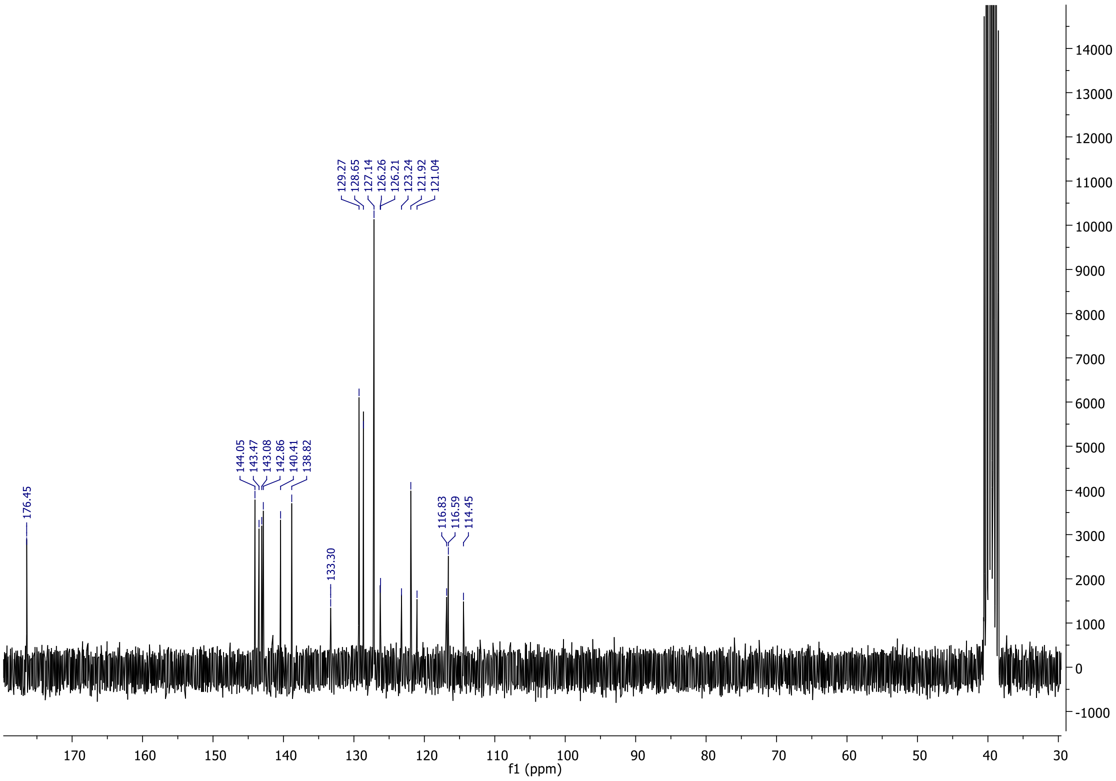

7-(dimethylamino)-1,3-diphenylacridin-9(10*H*)-one (3b)

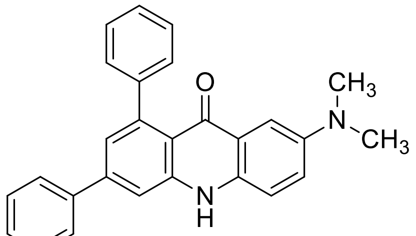

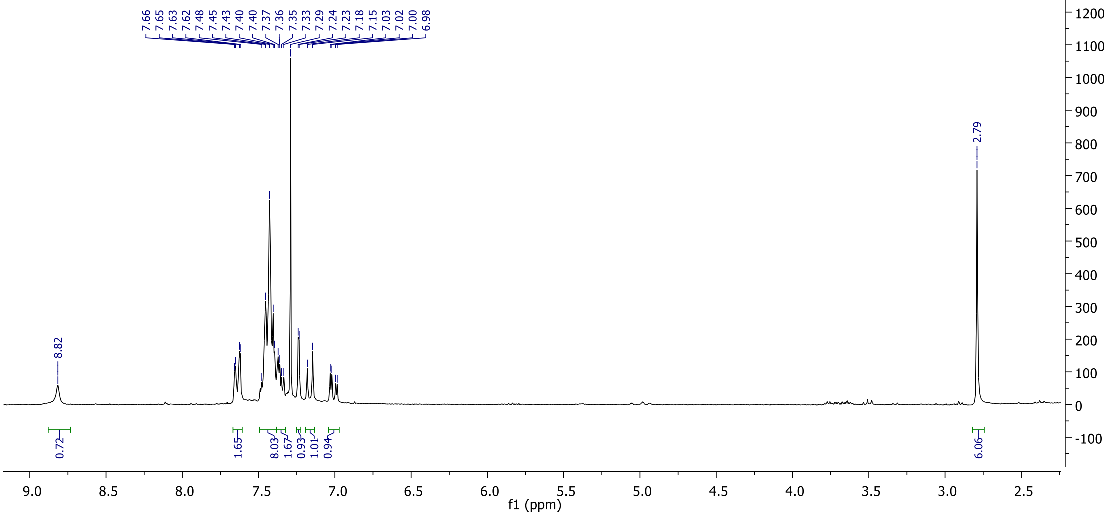

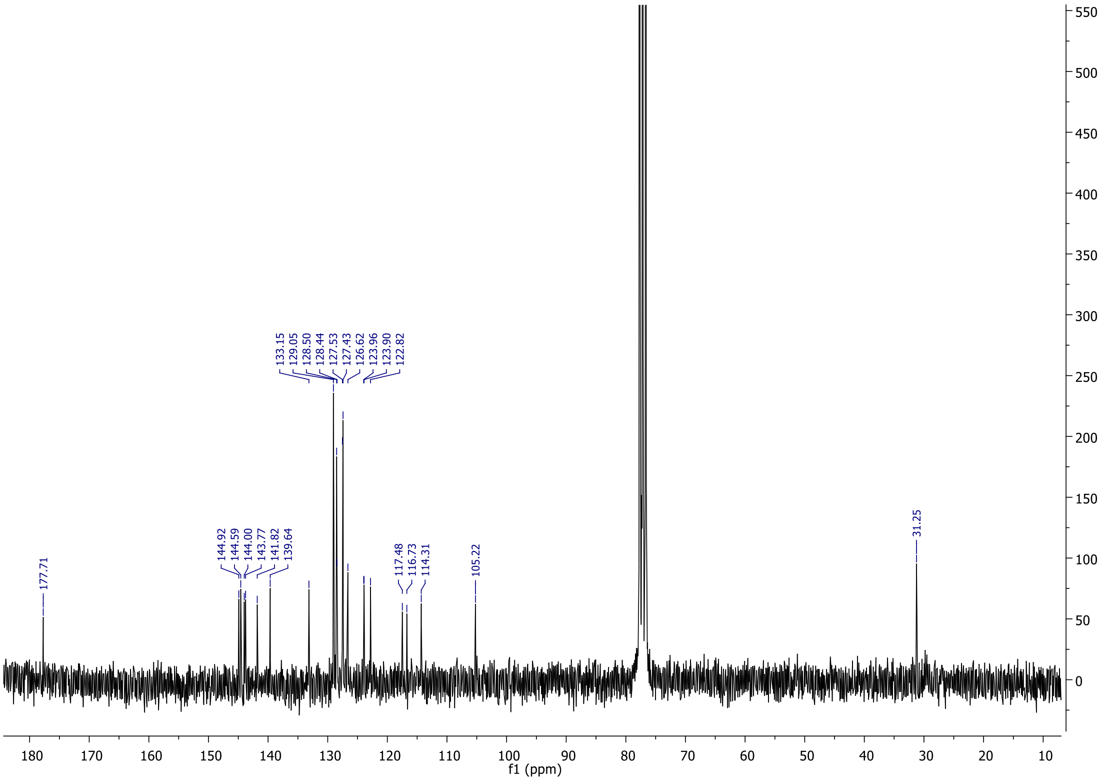

7-fluoro-1,3-diphenylacridin-9(10*H*)-one (3c)

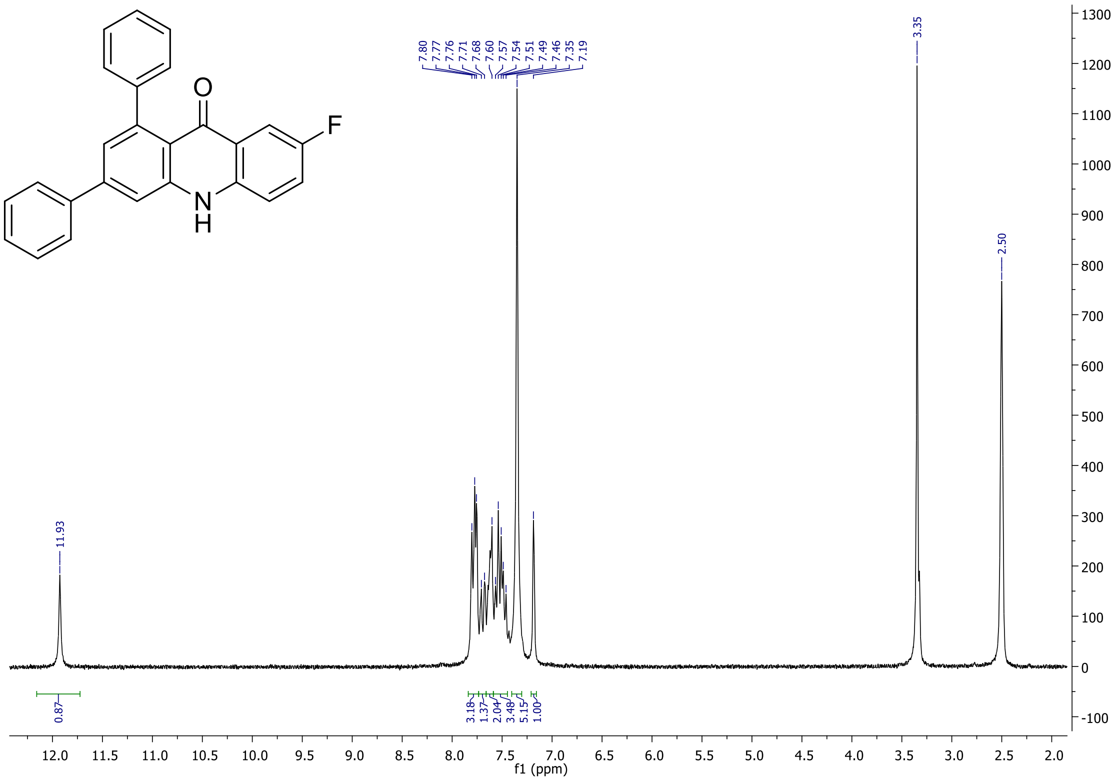

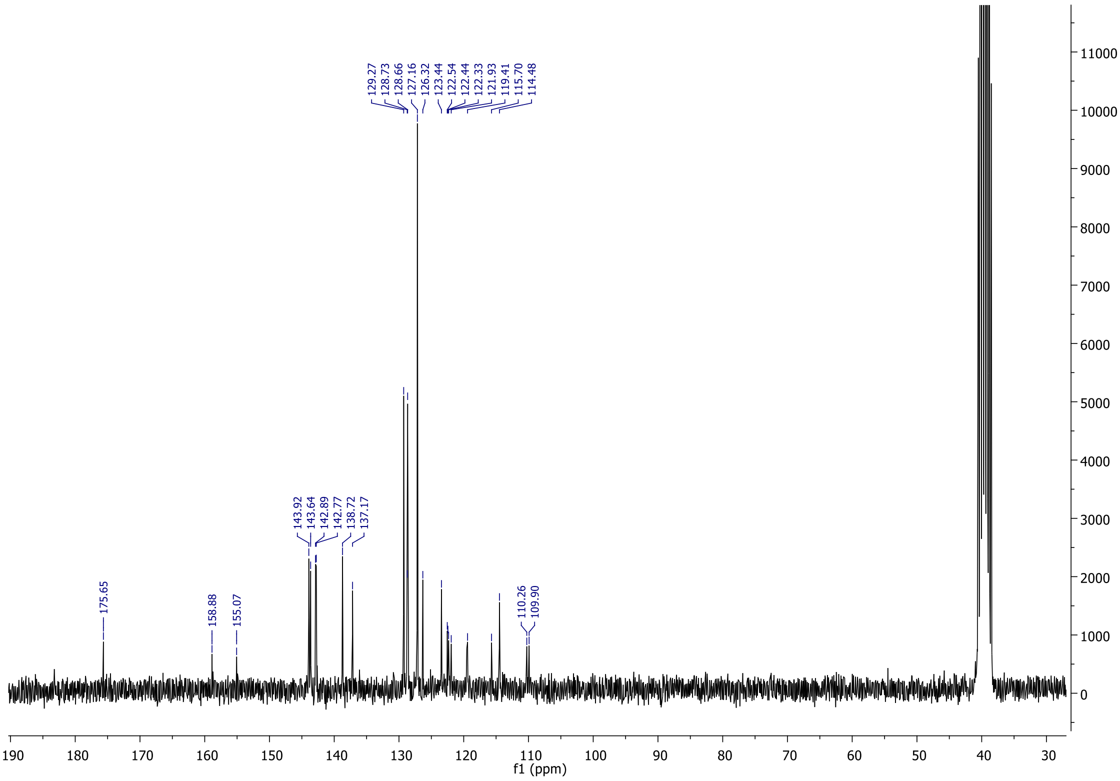

7-chloro-1,3-diphenylacridin-9(10*H*)-one (3d)

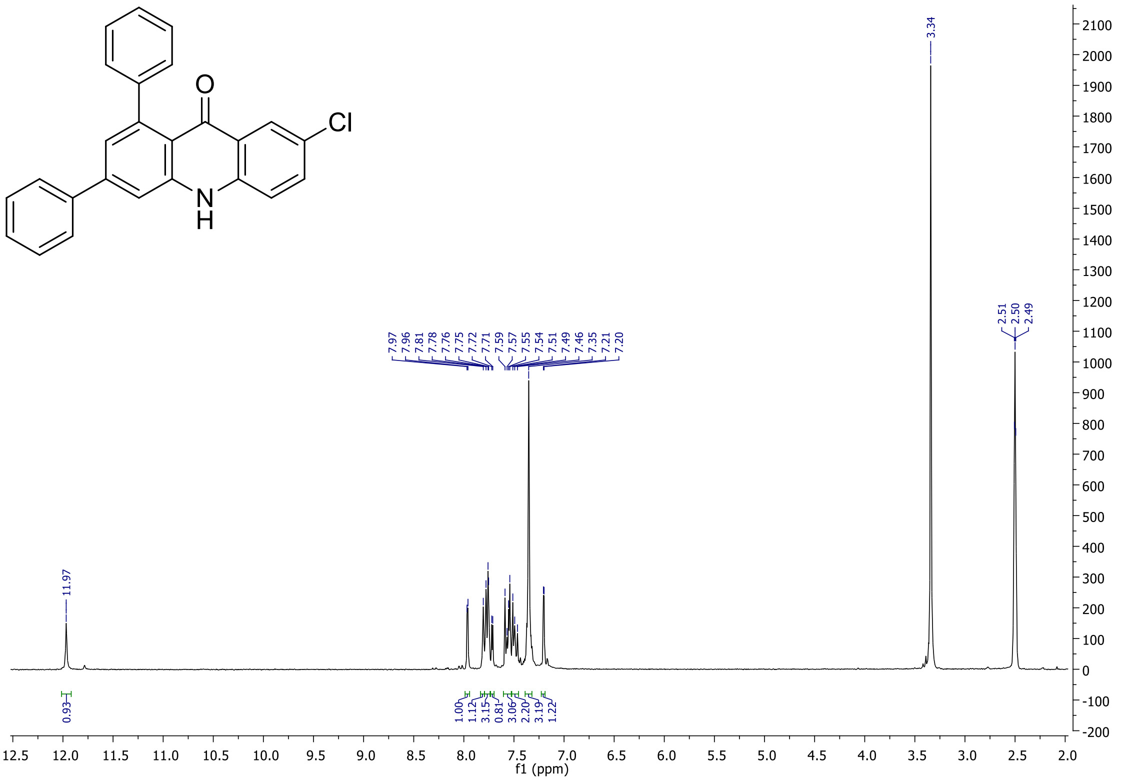

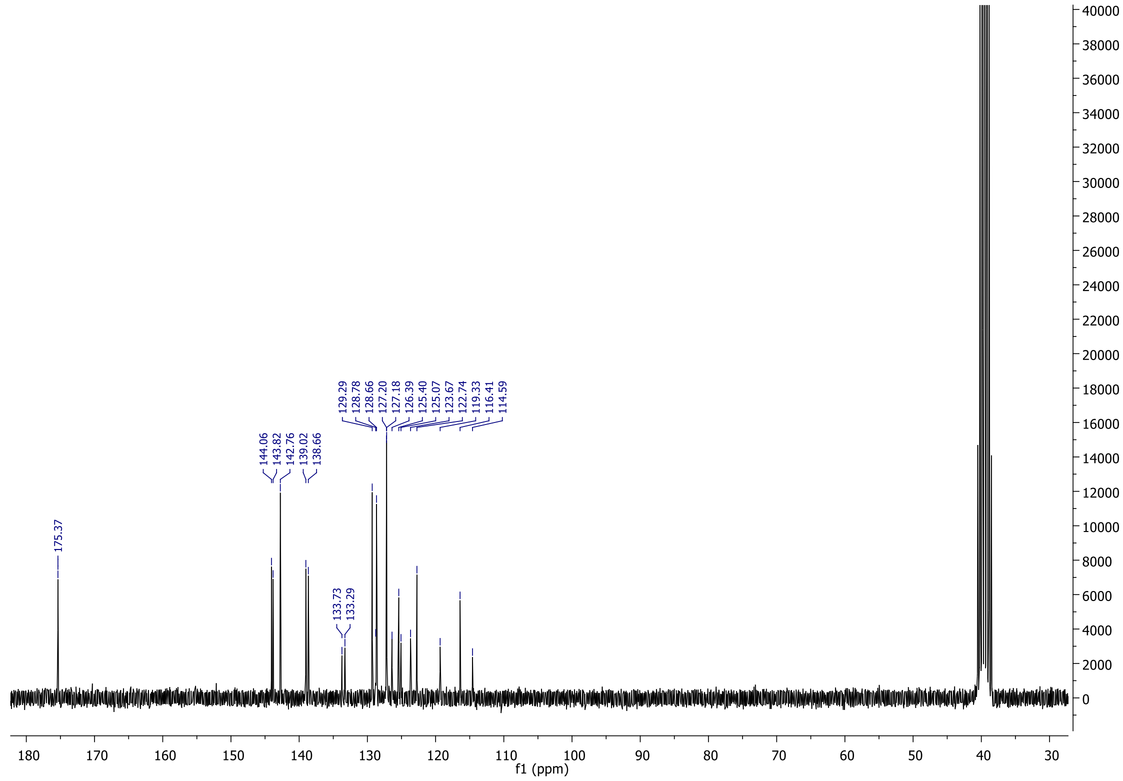

7-bromo-1,3-diphenylacridin-9(10*H*)-one (3e)

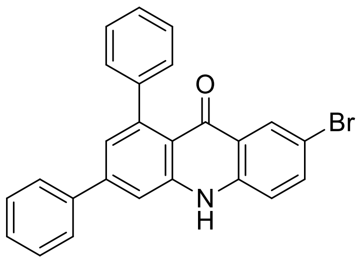

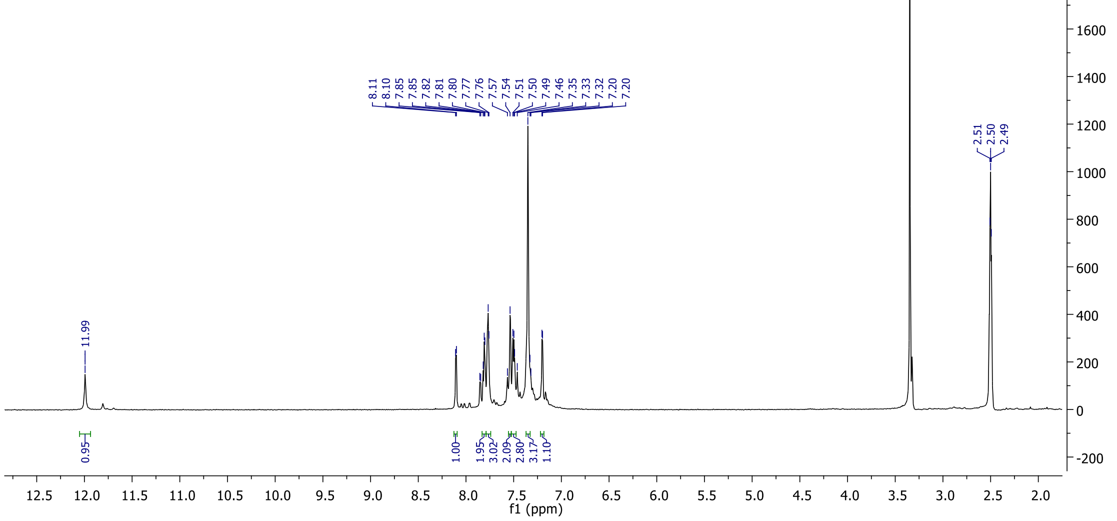

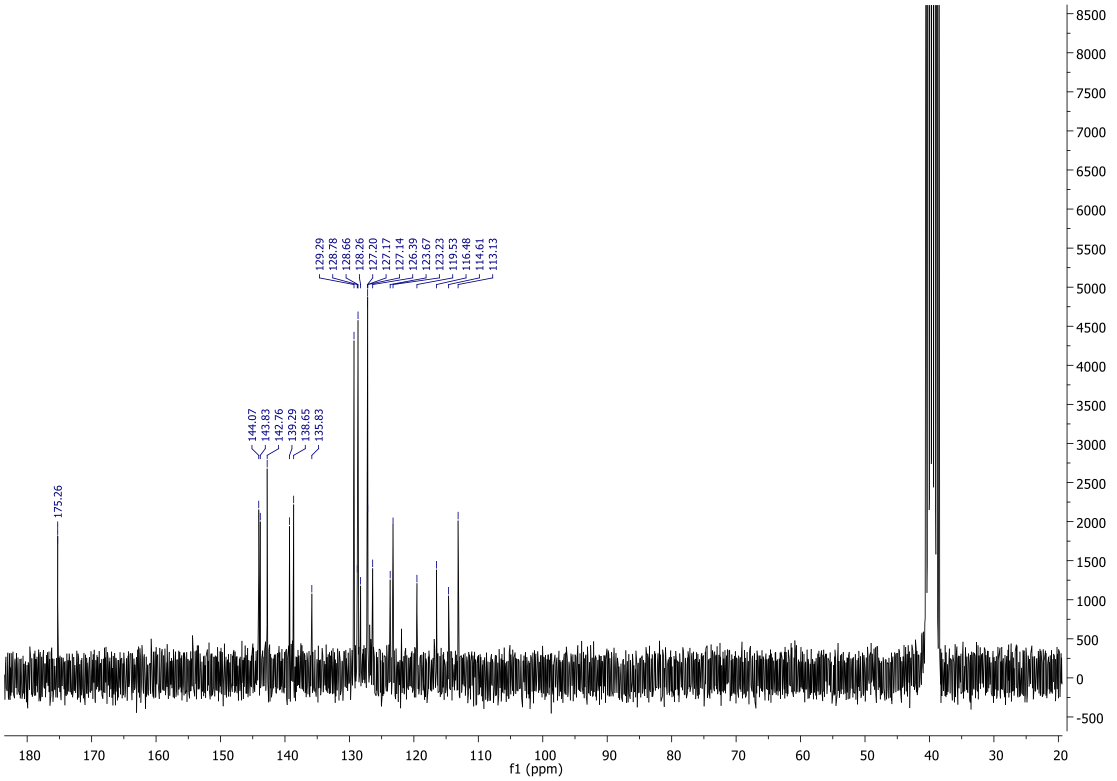

6,8-dichloro-1,3-diphenylacridin-9(10*H*)-one (3f)

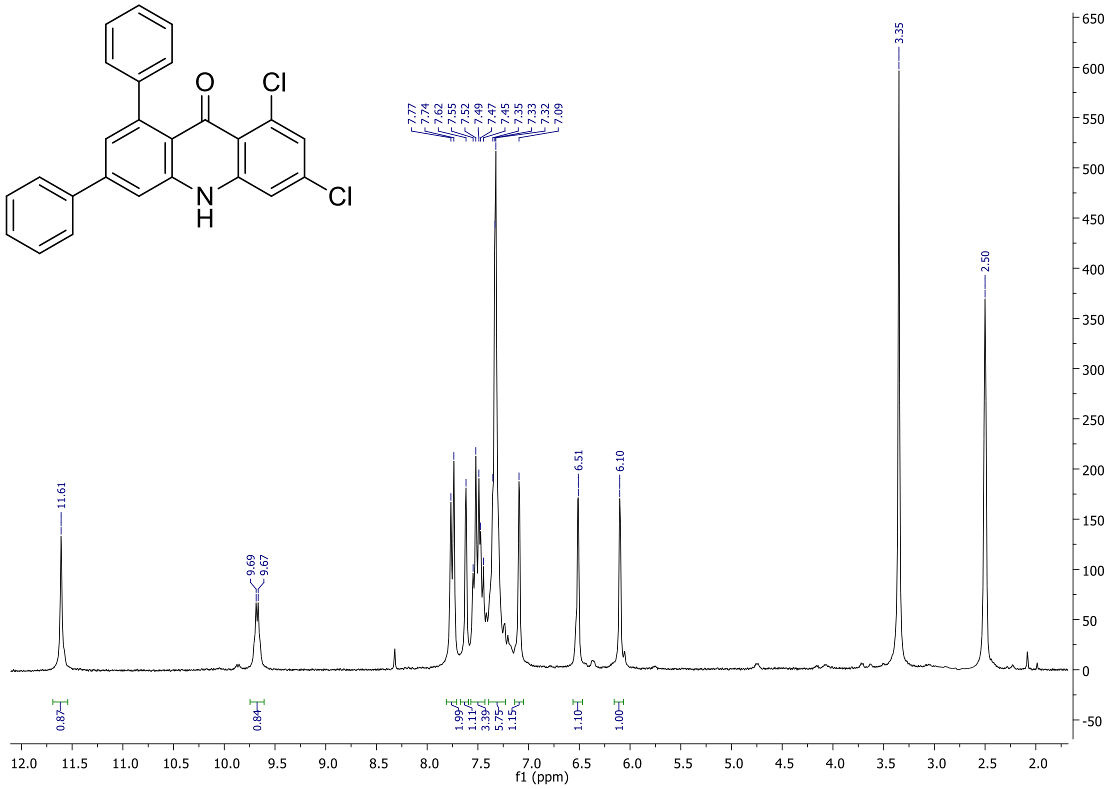

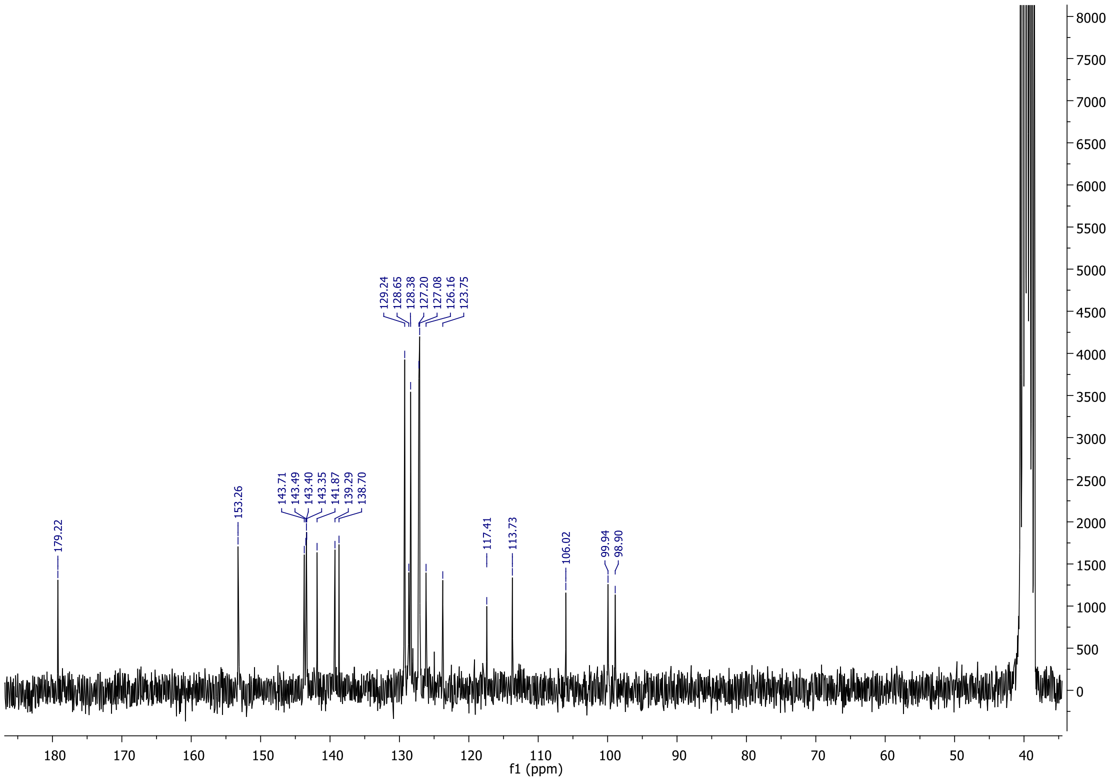

6,8-dimethyl-1,3-diphenylacridin-9(10*H*)-one (3g)

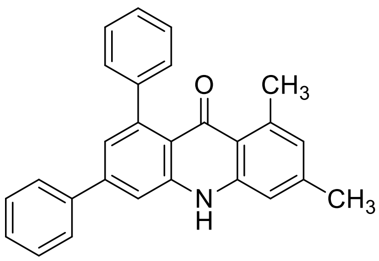

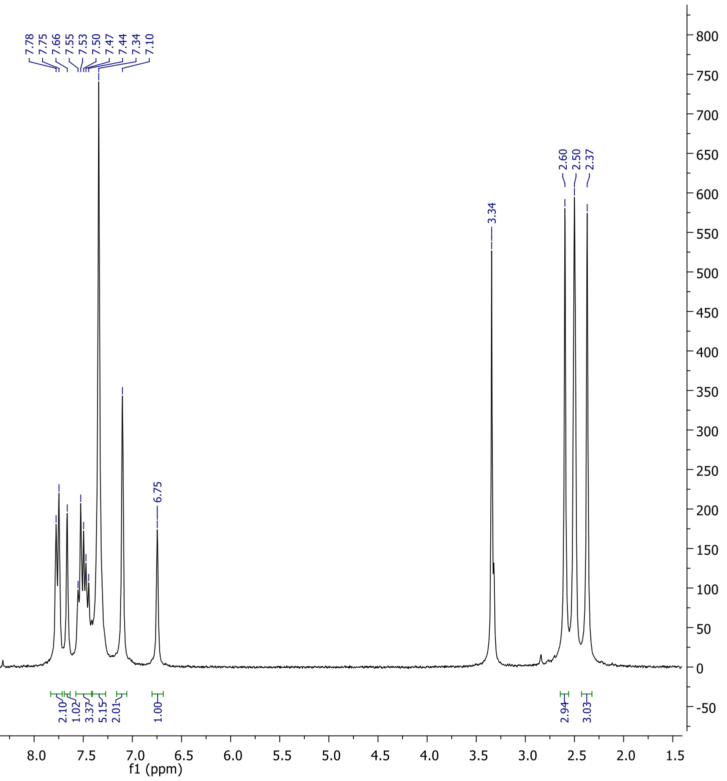

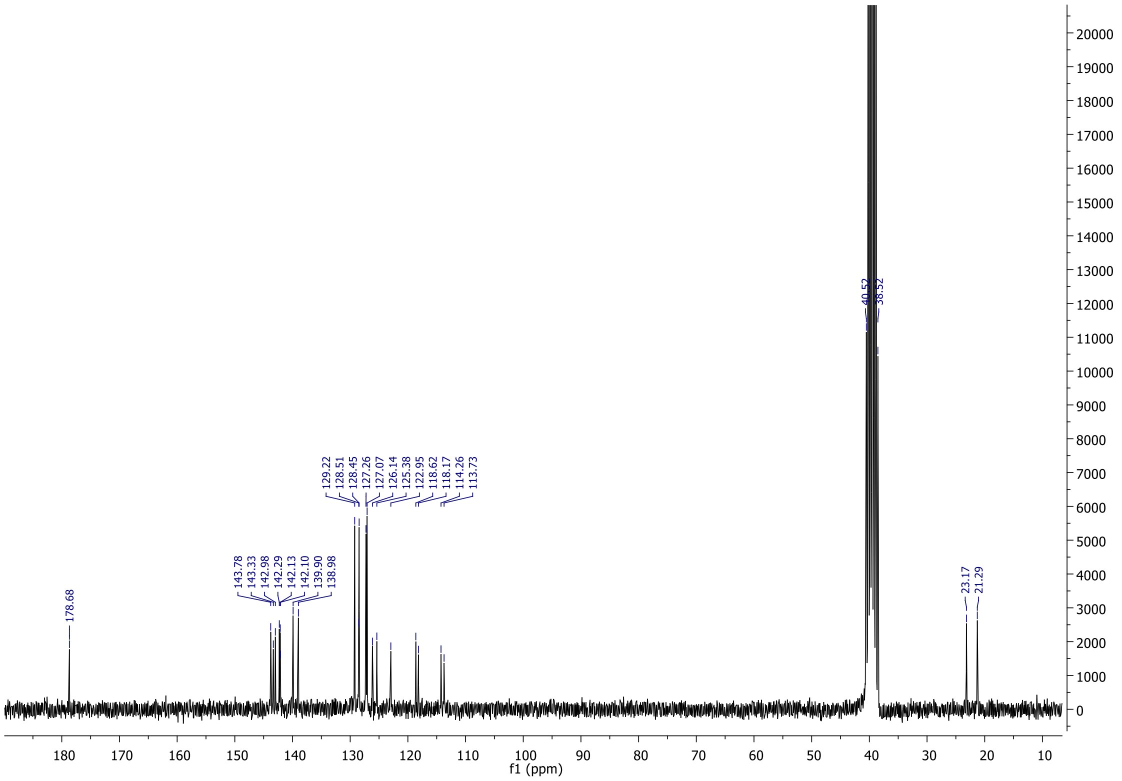

1-(4-nitrophenyl)-3-phenylacridin-9(10*H*)-one (3h)

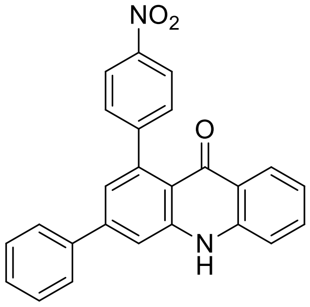

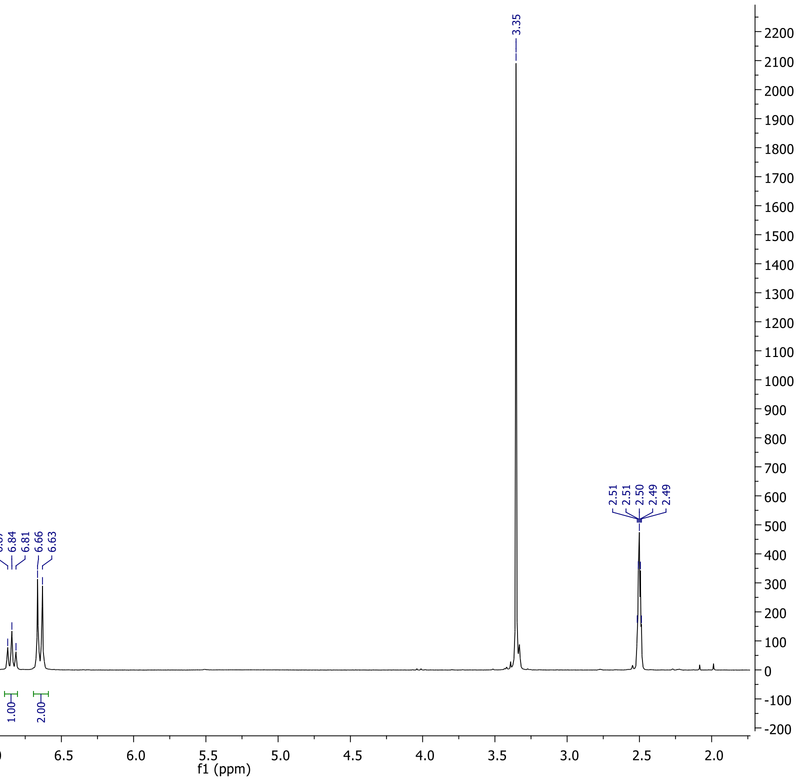

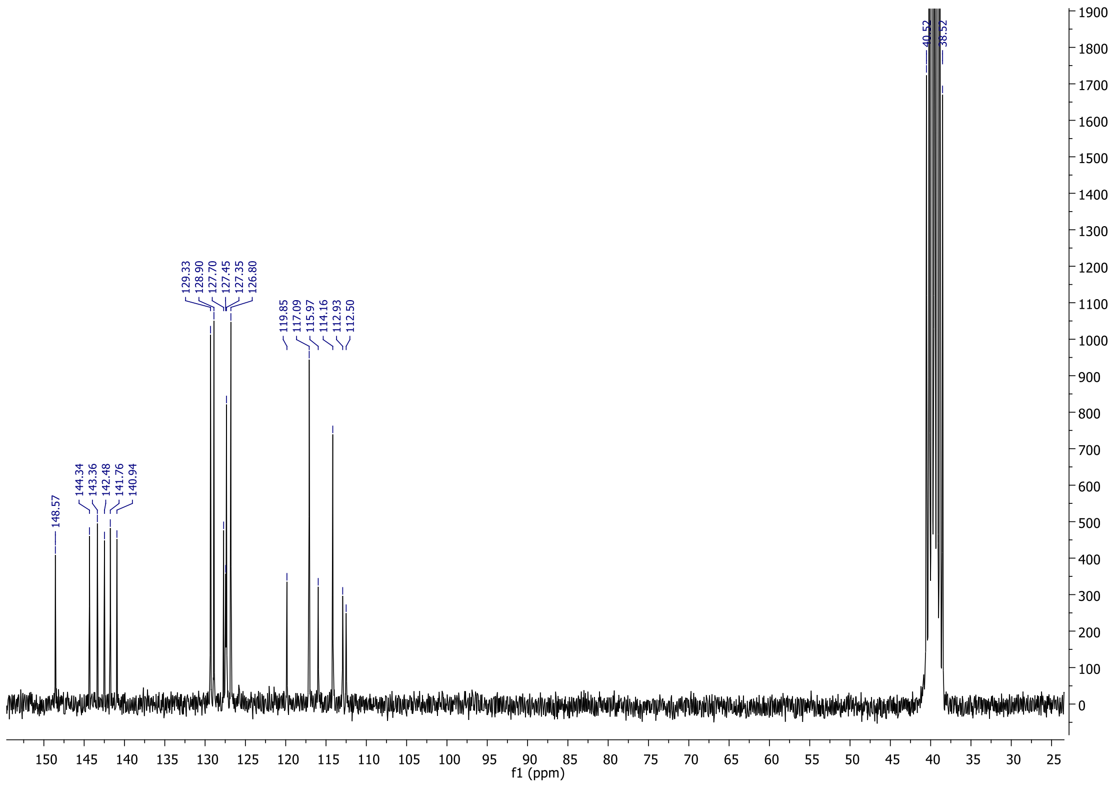

3-(4-bromophenyl)-1-phenylacridin-9(10*H*)-one (3i)

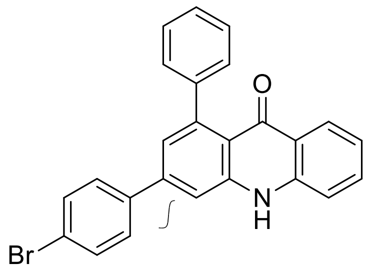

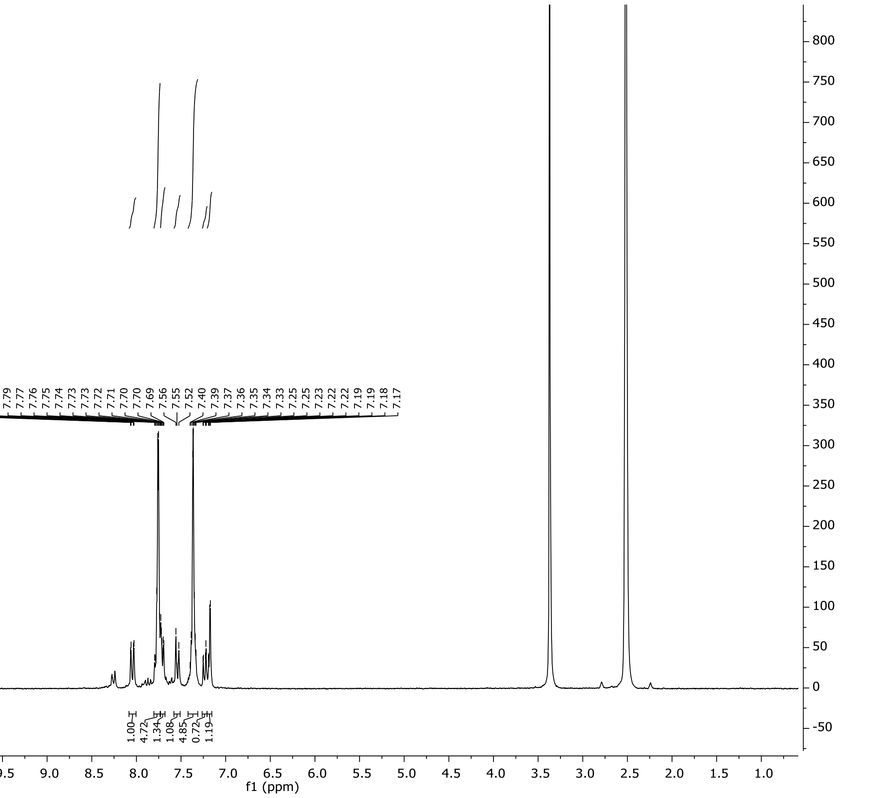

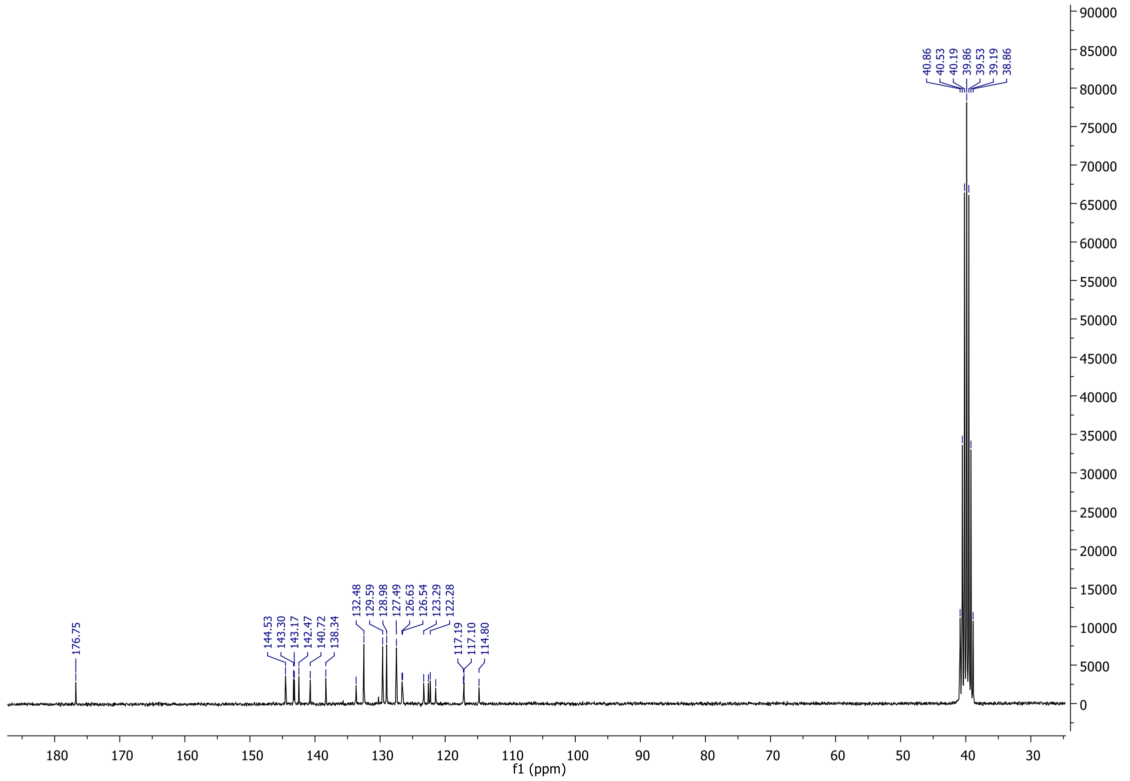

1-phenyl-3-(thiophen-2-yl)acridin-9(10*H*)-one (3j)

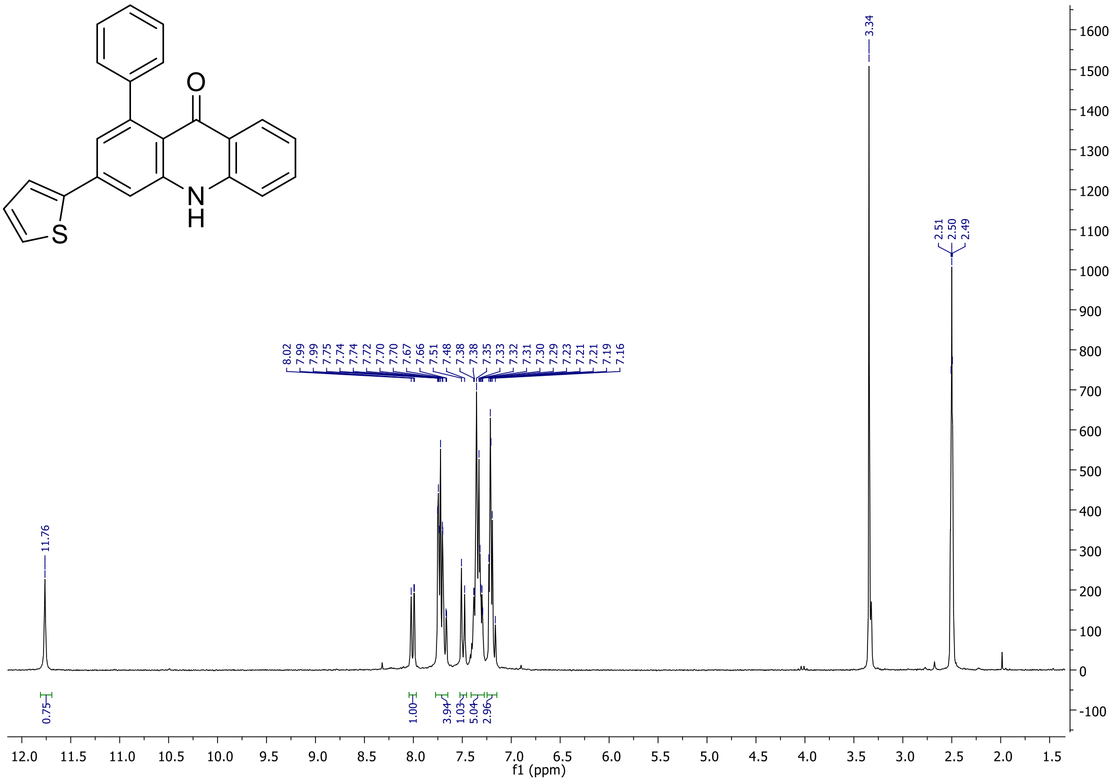

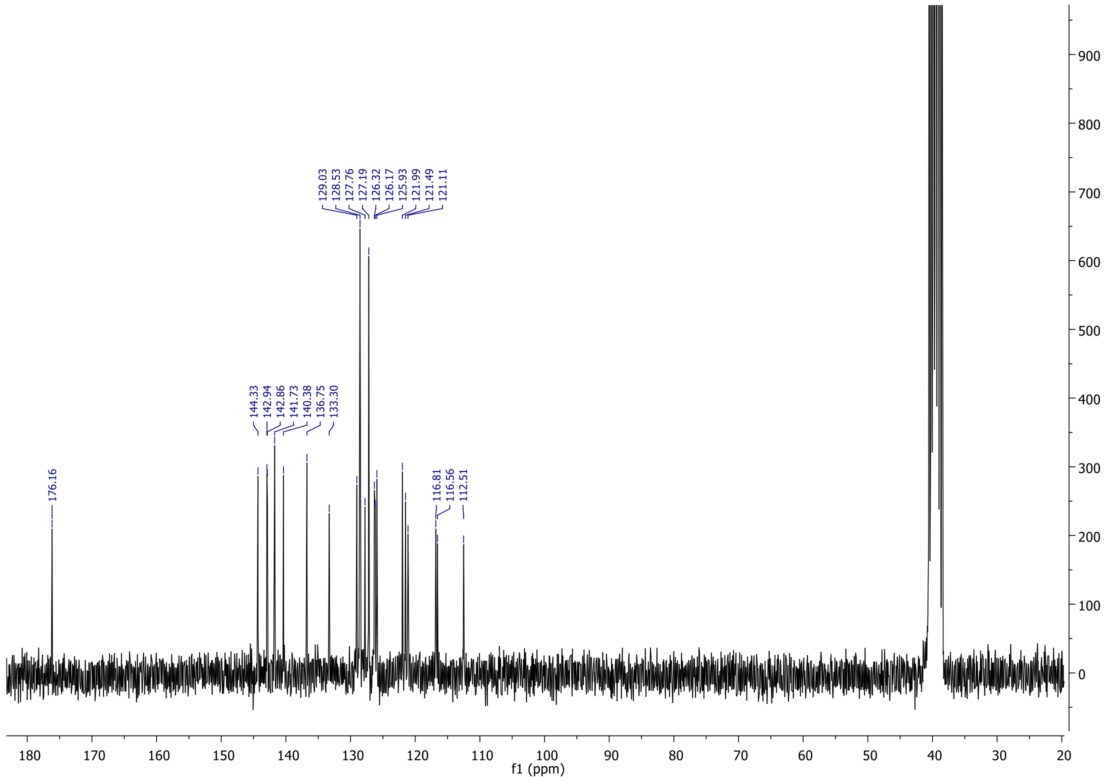

1,3-di(furan-2-yl)acridin-9(10*H*)-one (3k)

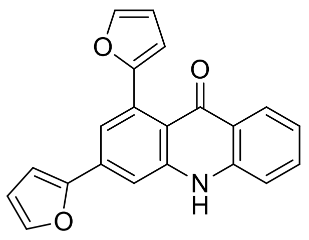

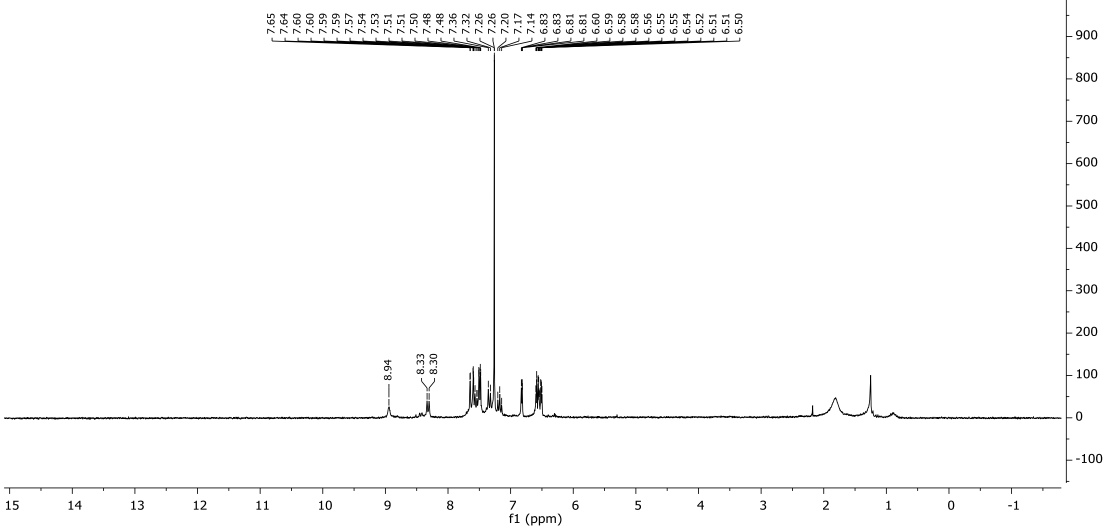

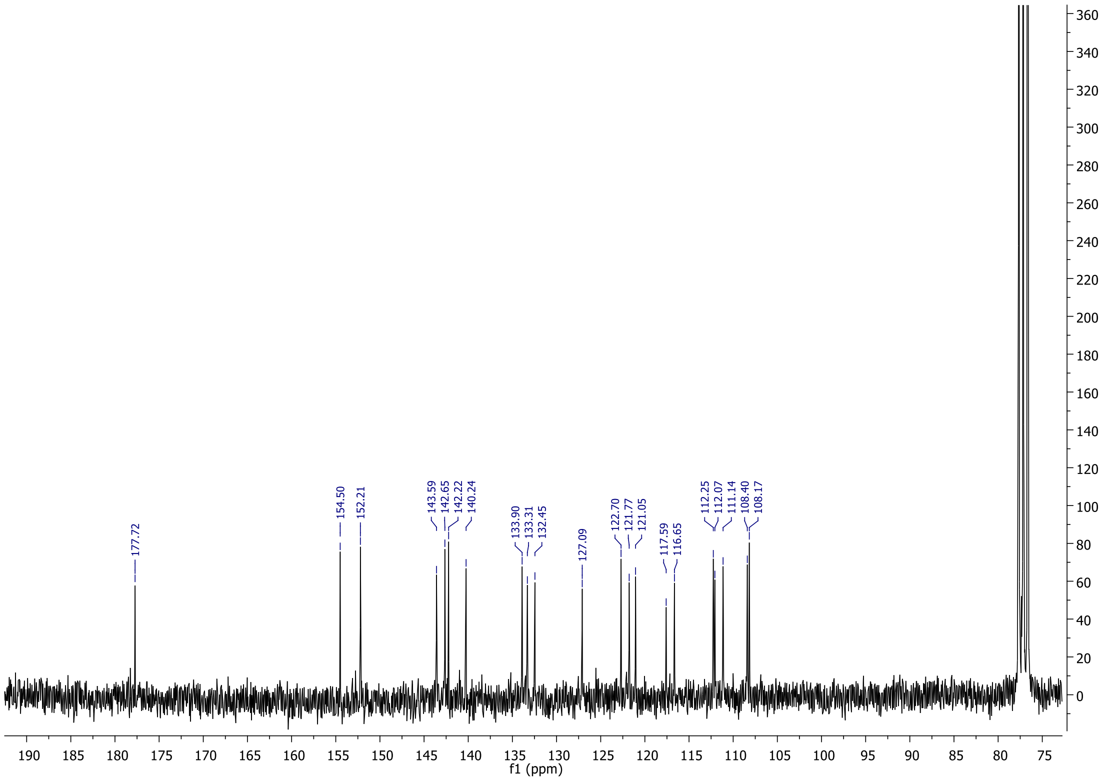

1-(4-methoxyphenyl)-3-phenylacridin-9(10H)-one (3l)

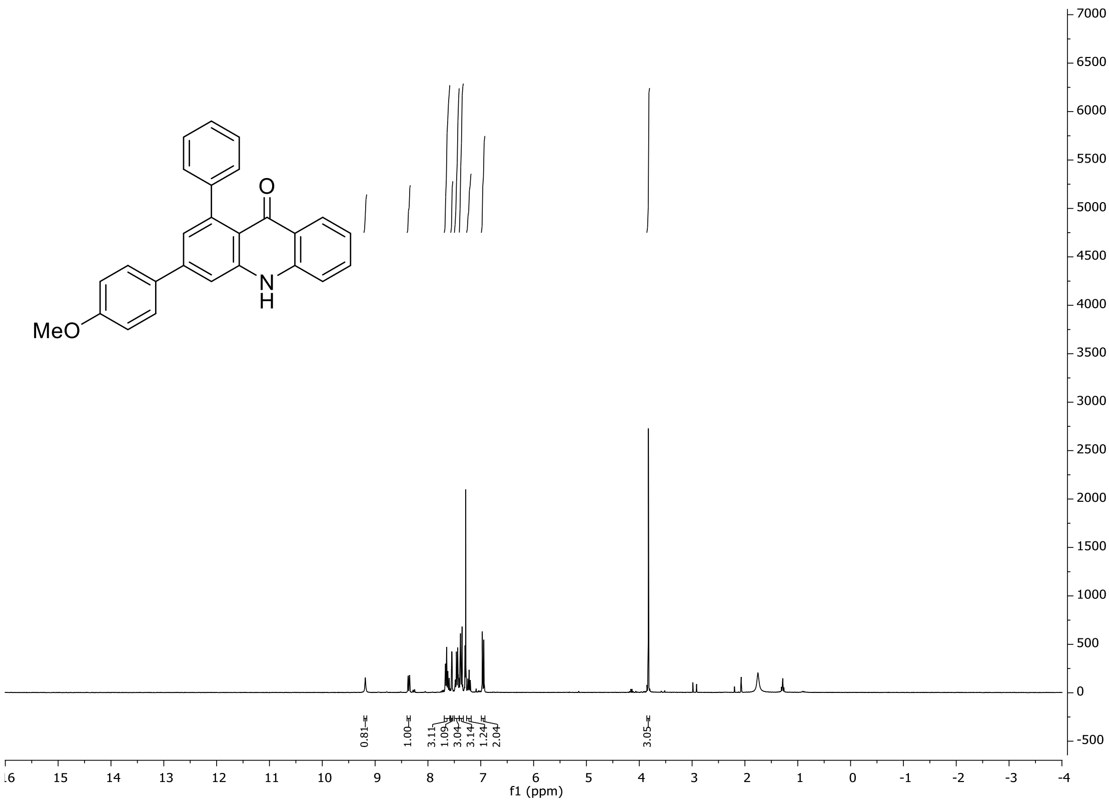

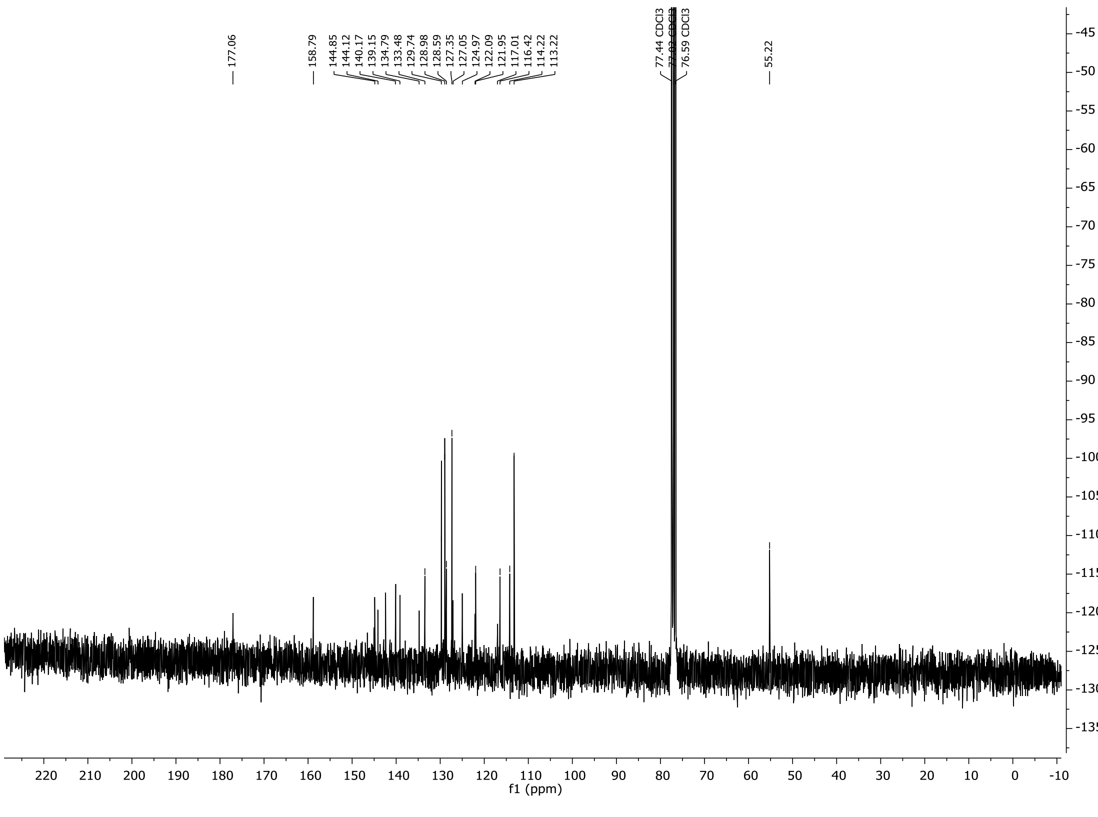

1-(4-chlorophenyl)-3-phenylacridin-9(10H)-one (3m)

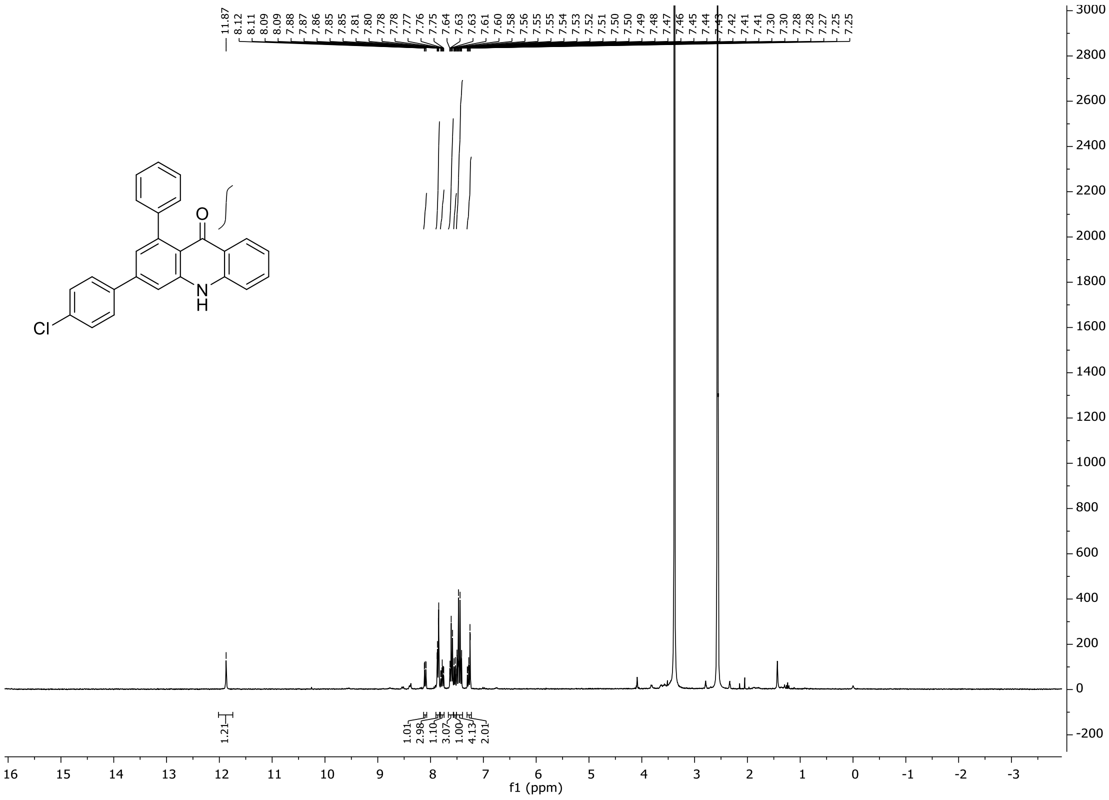

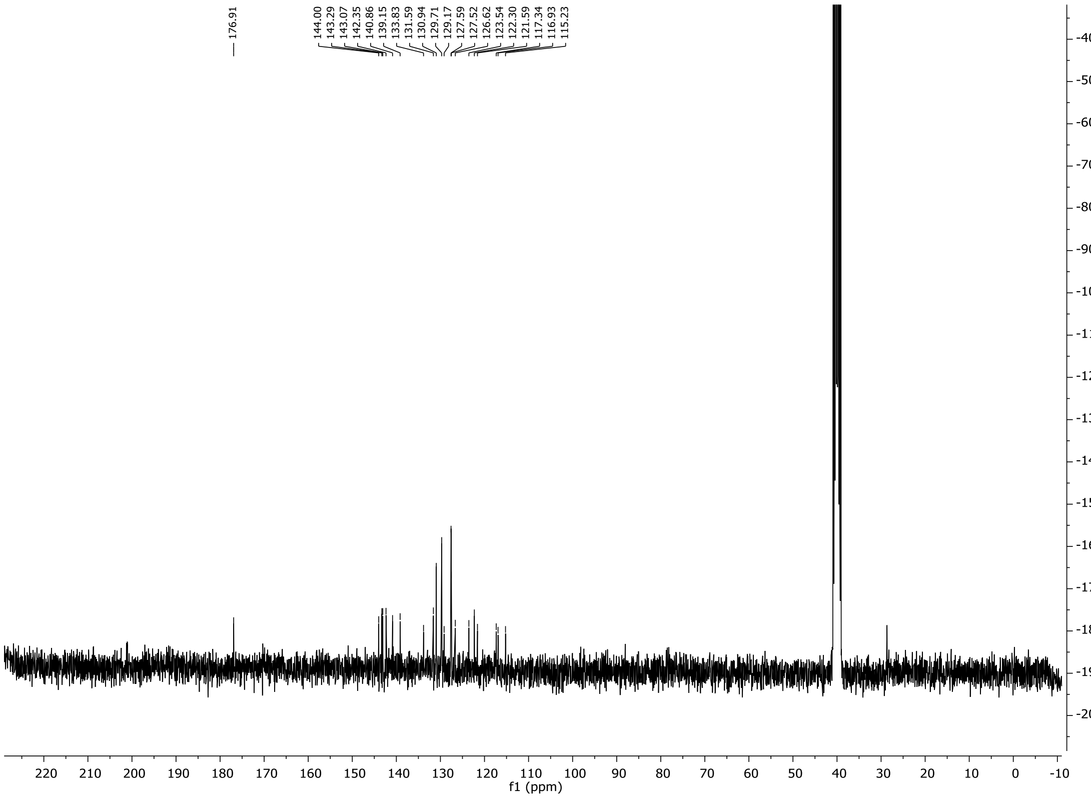

1-(2,4-dimethoxyphenyl)-3-(4-methoxyphenyl)-acridin-9(10H)-one (3n)

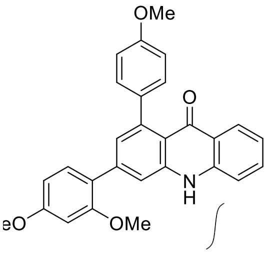

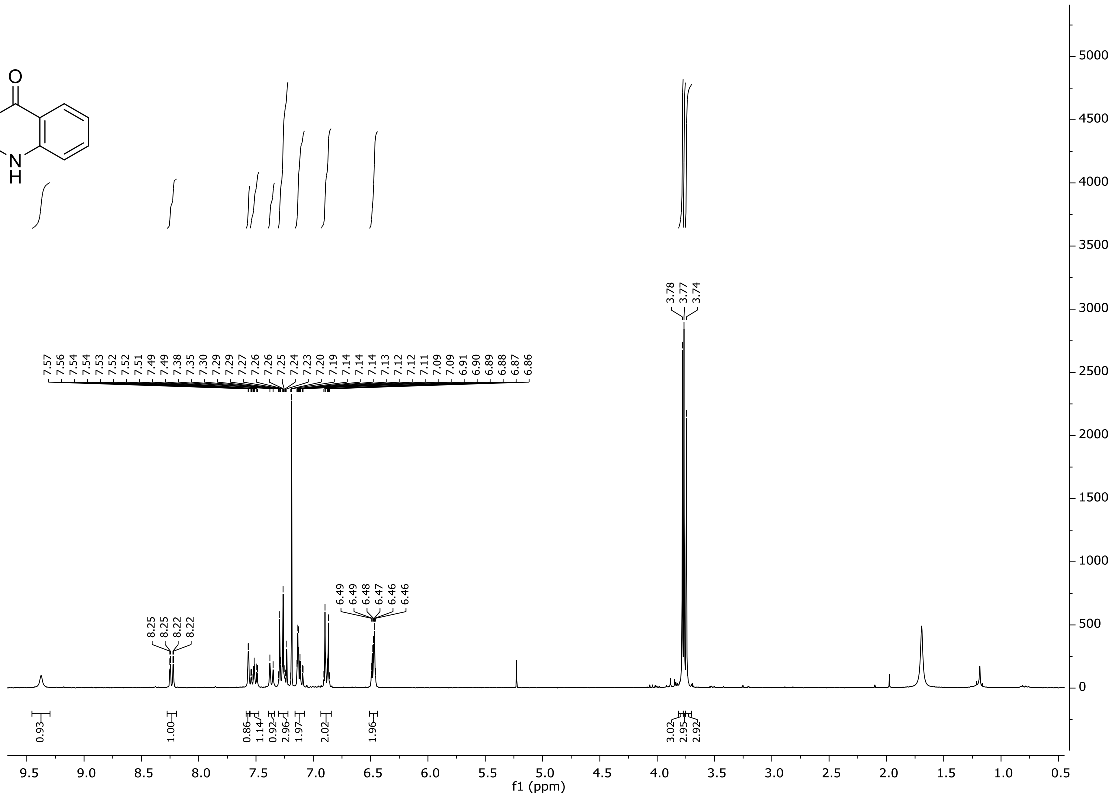

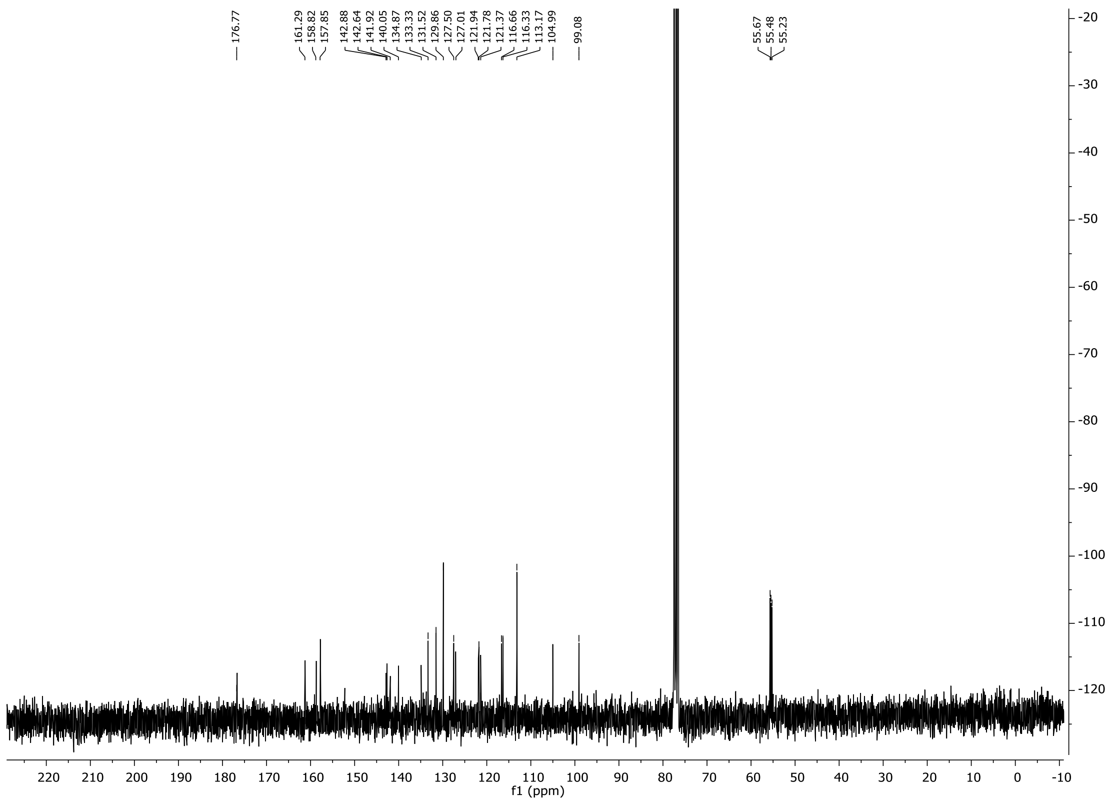

1,3-bis(4-chlorophenyl)acridin-9(10H)-one (3o)

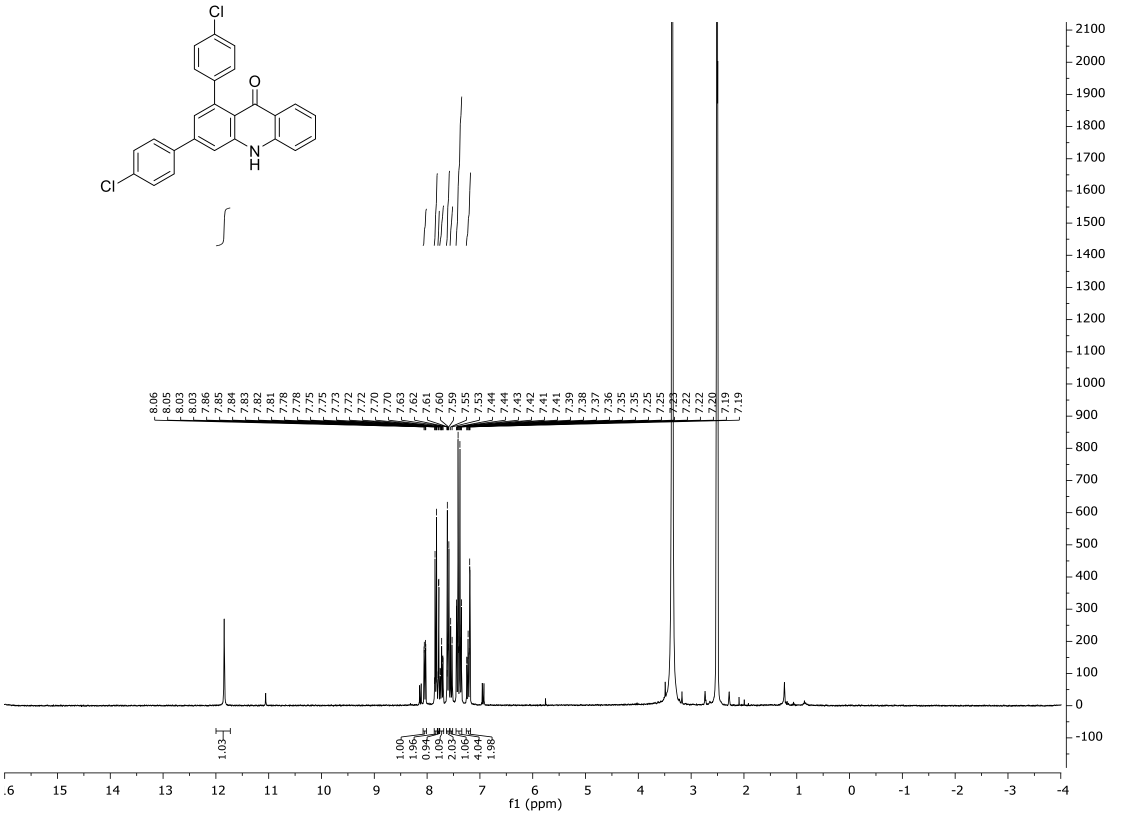

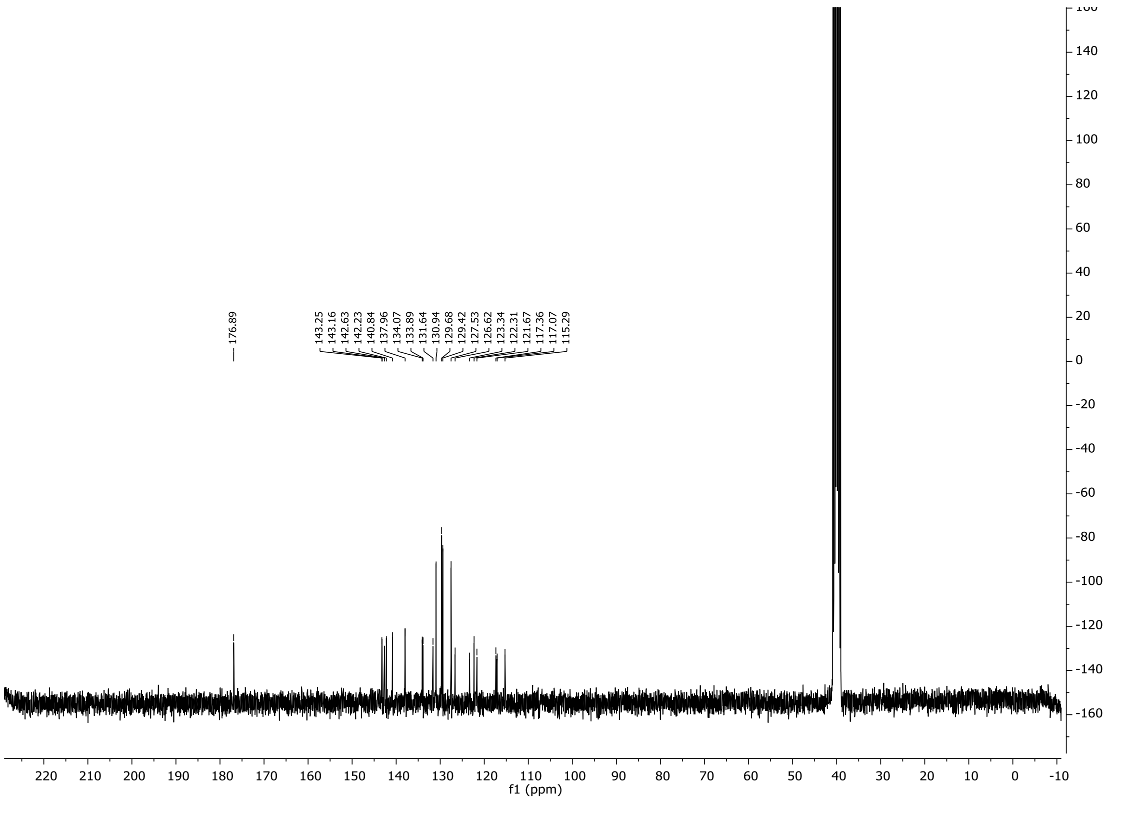

2. Structural study of compounds 2a y 3a.

2D-NMR study of compound 2a

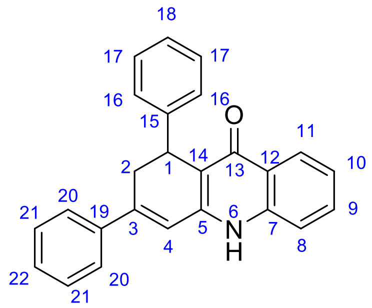

^{13}C chemical shift	HMQC	HMBC	Assignment
**33.8**	H-2	H-16	2
**34.0**	H-1	H-16	1
**113.7**		H-2	14
**117.2**	H-4		4
**118.0**	H-8	H-10	8
**122.8**	H-10	H-8	10
**125.1**	H-11	H-9	11
**125.5**	H-20		20
**126.1**	H-18	H-16	18
**127.1**	H-16	H-18	16
**128.6**	H-17		17
**129.0**	H-21		21
**129.1**	H-22		22
**131.4**	H-9	H-11	9
**138.8**		H-4, H20 and H-21	19
**139.3**		H-10, H-11	7 and 12
**143.6**			3
**144.5**			19
**145.4**		H-2	15
**174.4**		H-1	13

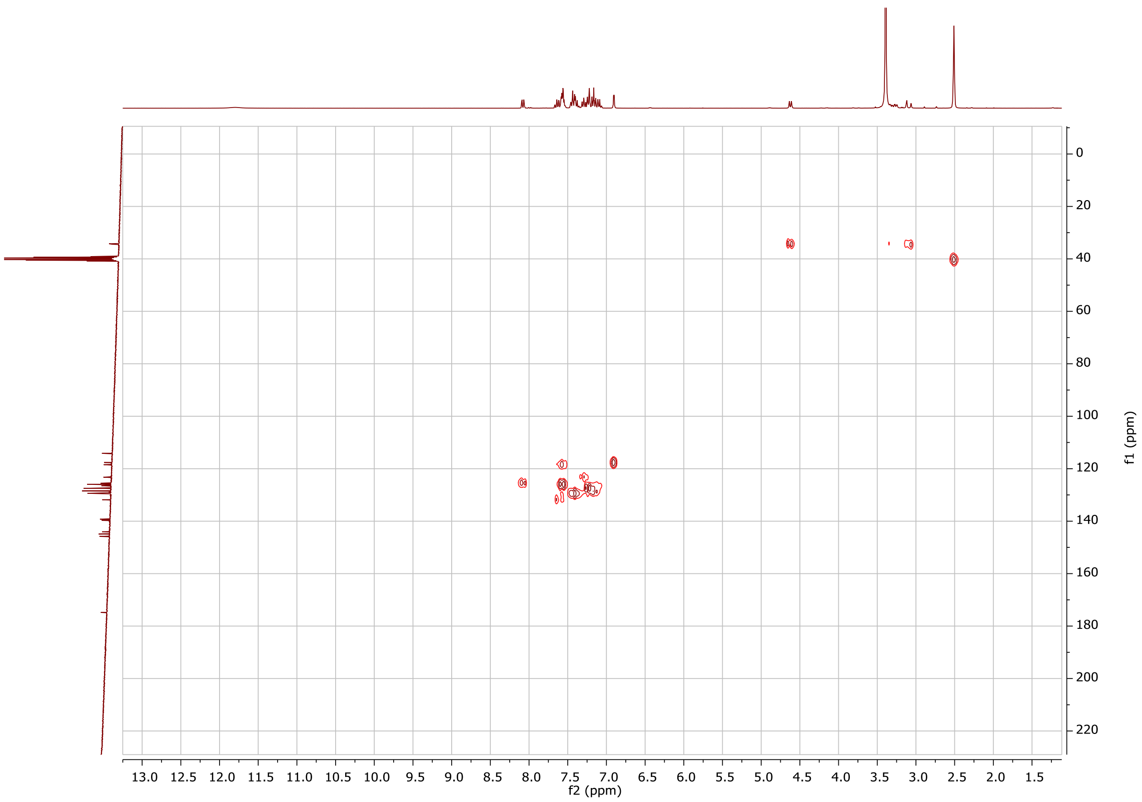
f2 (ppm)
f1 (ppm)

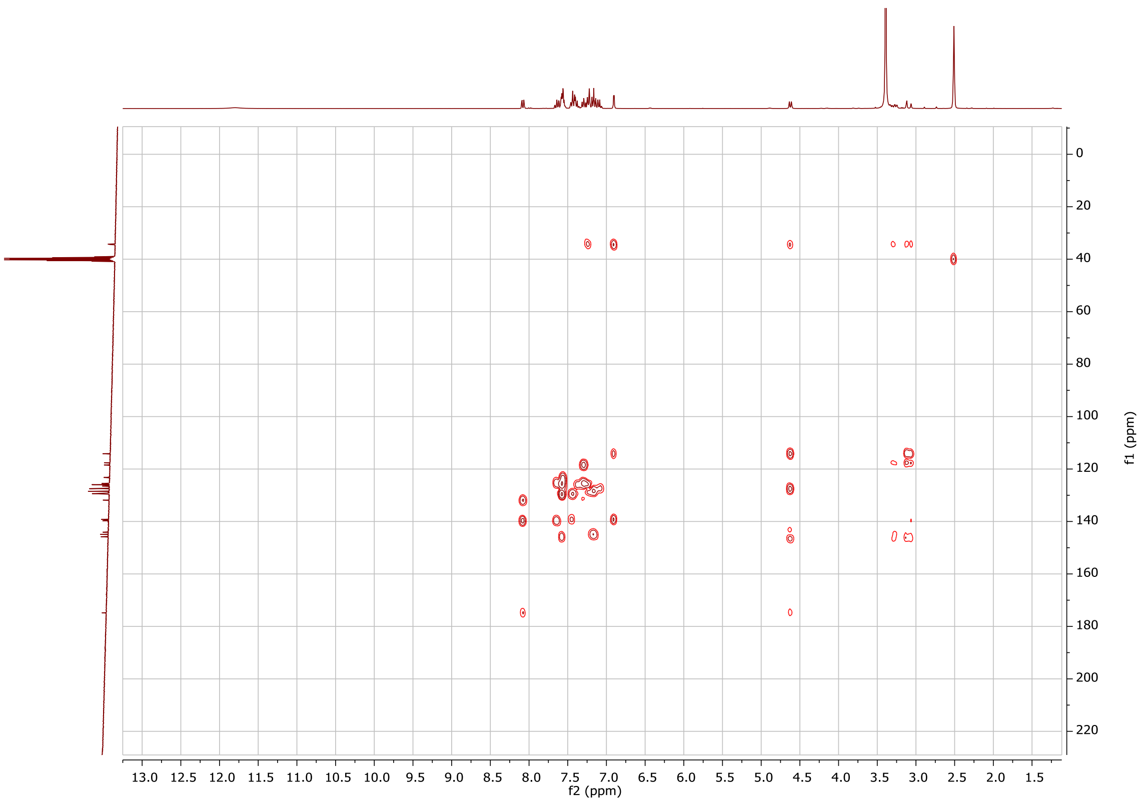
f2 (ppm)
f1 (ppm)

2D-NMR study of compound 3a

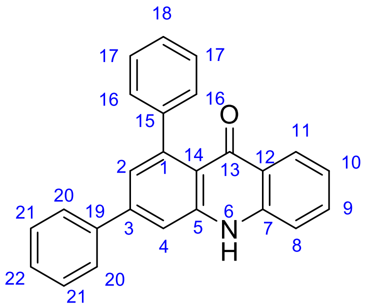

^{13}C chemical shift	HMQC	HMBC	Assignment
**114.4 (CH)**	H-4	H-2	4
**116.6 (C)**		H-6, H-10	12
**116.8 (CH)**	H-2	H-4	2
**121.0 (CH)**	H-10		10
**121.9 (C)**		H-6, H-2	14
**123.2 (CH)**	(H-16, 17, 20, 21 or 22	H-4, H-14, H-13	*
**126.2 (CH)**	H-11	H-9	11
**126.2 (CH)**	H-18		*
**127.1 (CH)**	H-8, H-21	H-3, H-5, H-7	*
**128.6 (CH)**	(H-16, 17, 20, 21 or 22)		*
**129.2 (CH)**	(H-16, 17, 20, 21 or 22)		*
**133.3 (CH)**	H-9	H-11	9
**138.1 (C)**			*
**140.4 (C)**		H-11, H-9	7
**142.9 (C)**			*
**143.1 (C)**			*
**143.5 (C)**			*
**144.0 (C)**			*
**176.5 (C=O)**			13

* The signal was not able to be uniquely assigned

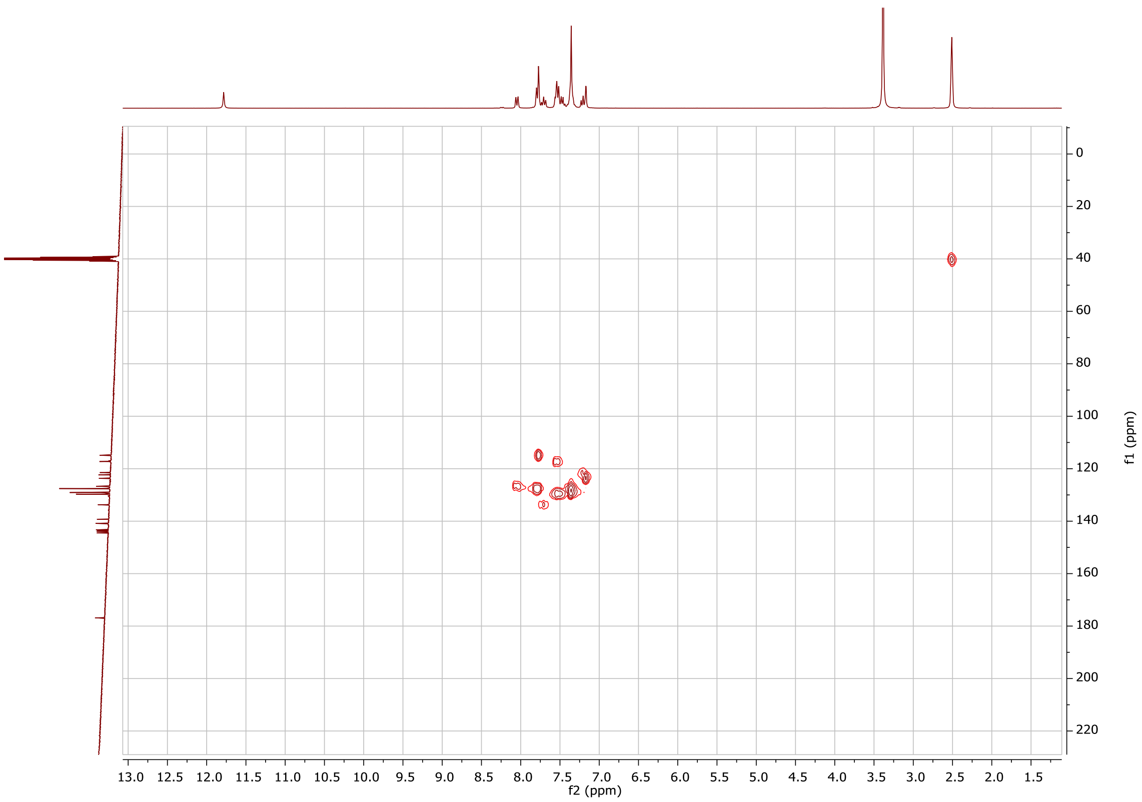

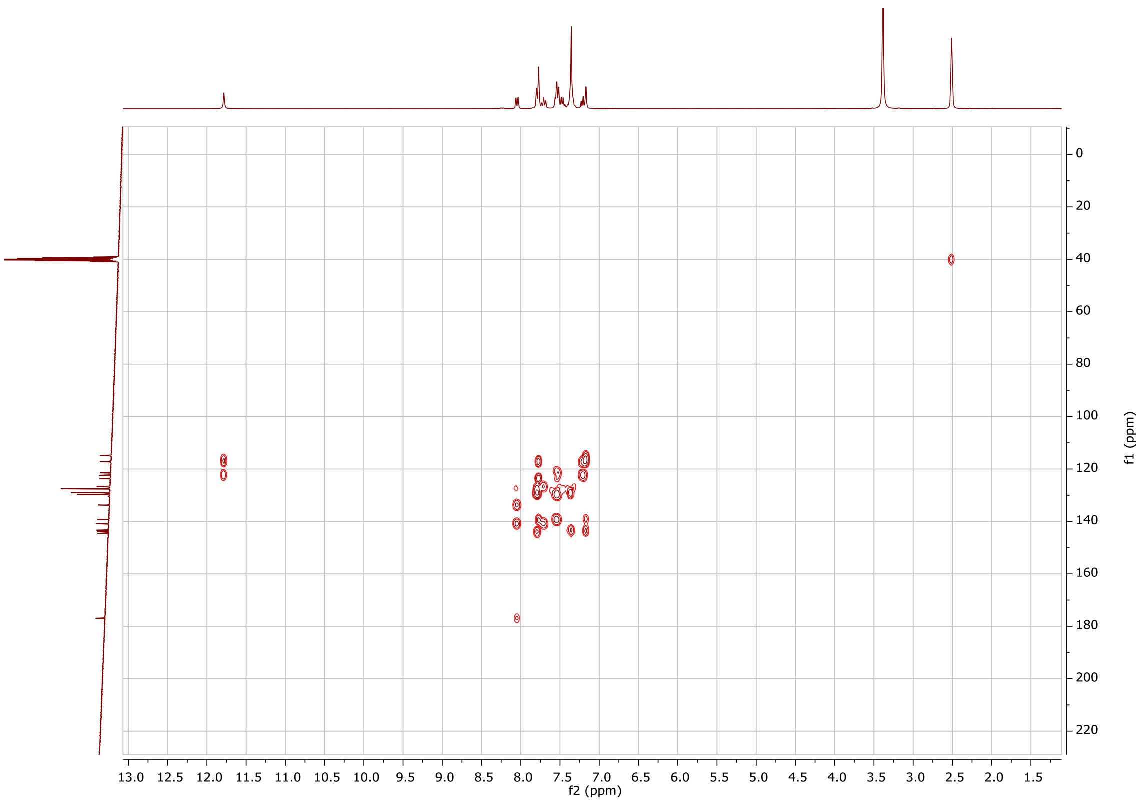

^{1}H NMR (250 MHz, Chloroform-*d*) δ 8.94 (s, 1H), 8.32 (d, *J* = 8.1 Hz, 1H), 7.65 (d, *J* = 1.7 Hz, 1H), 7.61 – 7.53 (m, 2H), 7.52 – 7.49 (m, 1H), 7.48 (d, *J* = 1.6 Hz, 1H), 7.34 (d, *J* = 8.3 Hz, 1H), 7.17 (m, 1H), 6.86 – 6.78 (d, *J* = 3.8 Hz, 1H), 6.59 (dd, *J* = 3.2, 0.9 Hz, 1H), 6.55 (dd, *J* = 3.3, 1.8 Hz, 1H), 6.51 (dd, *J* = 3.4, 1.8 Hz, 1H).
